# Copper-catalyzed stereospecific methoxyboration of styrenes enabled by oxygen umpolung with acetal-based peroxides

**DOI:** 10.1039/d5sc07347b

**Published:** 2025-10-10

**Authors:** Kyosuke Fujiwara, Shogo Nakamura, Koji Hirano

**Affiliations:** a Department of Applied Chemistry, Graduate School of Engineering, Osaka University Suita Osaka 565-0871 Japan k_hirano@chem.eng.osaka-u.ac.jp; b Innovative Catalysis Science Division, Institute for Open and Transdisciplinary Research Initiatives (ICS-OTRI), Osaka University Suita Osaka 565-0871 Japan

## Abstract

An oxygen-umpolung-enabled, regioselective and stereospecific copper-catalyzed methoxyboration of styrenes with diborons and acetal-based methyl peroxide has been developed. The use of designed peroxide enables the otherwise difficult two-electron redox event under the borylcopper catalysis, thus delivering the corresponding oxyborated products with high stereospecificity. Combined with the stereospecific post functionalizations of the boron moiety, the copper catalysis can provide facile access to the stereochemically defined, functionality-rich alkyl ether derivatives ubiquitously found in bioactive molecules and functional materials.

## Abstract 2

An oxygen-umpolung-enabled, stereospecific Cu-catalyzed methoxyboration of styrenes with B_2_pin_2_ and acetal-based methyl peroxide has been developed. The designed peroxide enables the otherwise difficult two-electron redox event under Cu catalysis.

## Graphical abstract

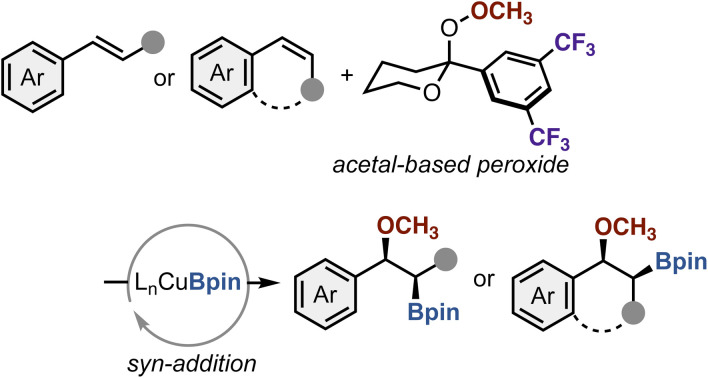

## Introduction

**Scheme 1:**
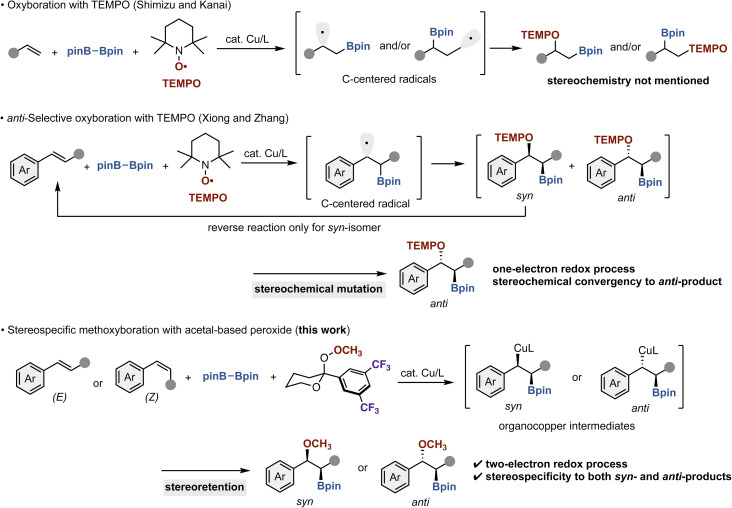
Stereochemistry in copper-catalyzed oxyboration of alkenes.

Organoboron compounds are ubiquitous and indispensable in modern organic synthetic chemistry because the C–B bonds can be selectively and stereospecifically converted to various C–C and C–heteroatom bonds under specific conditions, which are particularly useful for convergent synthesis of complex molecules such as bioactive molecules and natural products.^1^ Moreover, several organoboron compounds themselves are important targets with unique biological activities.^2^ Accordingly, the development of synthetic methods for functionality-rich and stereochemically defined organoboron compounds is one of long-standing research subjects in the synthetic community. Among numerous reported protocols, the borylative difunctionalizations of readily available and simple alkene substrates are highly attractive from the synthetic point of view. There are many successful reports of diboration, silaboration, carboboration, stannylboration, and aminoboration.^1*d*,3^ However, the catalytic simultaneous addition of oxygen and boron atoms, namely, oxyboration and alkoxyboration, still remains underdeveloped. In particular, the stereoselective and/or stereospecific variants are unmet challenges. In 2015, Shimizu, Kanai, and co-workers reported the copper-catalyzed oxyboration of terminal alkenes with bis(pinacolato)diboron (pinB–Bpin) and 2,2,6,6-tetramethylpiperidine 1-oxyl (TEMPO; Scheme 1a).^4^ While the regioselectivity was divergently controlled by the suitable choice of ancillary ligands, the reaction process involved the carbon radical intermediates and only employed terminal alkenes, thus not focusing the stereochemistry (diastereoselectivity) of the reaction. More recently, Xiong and Zhang developed the copper-catalyzed *anti*-selective oxyboration of β-substituted styrenes (Scheme 1b), where similar carbon-centered radical species are involved and a mixture of *syn*- and *anti*-diastereomers are initially formed, but stereochemical mutation process occurs *via* the selective reverse reaction of *syn*-isomer to the starting styrene.^5^ As a result, the corresponding *anti*-product was selectively formed in a stereoconvergent manner. Although this strategy allowed for the stereoselective preparation of oxyboration product with high *anti*-stereochemistry, the opposite isomer, *syn*-oxyboration product, cannot be obtained. Moreover, the overall process is stereoconvergent, and both (*E*)- and (*Z*)-styrenes are converted to the same *anti*-isomers. Thus, the stereospecific process, in which the (*E*)- and (*Z*)-styrenes are selectively converted to the corresponding *syn*- and *anti*-products, respectively (or *vice versa*), is not achieved yet but in high demand.

Meanwhile, our group recently focused on polarity inversion (umpolung) concept of oxygen^6^ and developed the highly stereospecific hydroalkoxylation of styrenes with hydrosilanes and designed acetal-based peroxides.^7^ The unique two-electron redox character of peroxides enabled the net hydroalkoxylation with perfect stereospecificity under copper hydride catalysis. During the continuing interest in this chemistry, we here report a copper-catalyzed methoxyboration of styrenes with pinB–Bpin and acetal-based methyl peroxide (Scheme 1c). The use of peroxide promotes the two-electron redox process rather than the previously observed one-electron redox process, thus leading to the high stereospecificity, which is difficult to achieve by other means.

## Results and discussion

Our optimization studies commenced with (*E*)-β-methylstyrene [(*E*)-1a], pinB–Bpin, and 3,5-(CF_3_)_2_C_6_H_4_-substituted acetal-based peroxide 2, which was optimal in the previous hydroalkoxylation,^7*a*^ to identify the suitable catalyst system (Table 1). In an early experiment, treatment of (*E*)-1a (0.10 mmol), pinB–Bpin (2.5 equiv.), and 2 (1.5 equiv.) in the presence of Cu(OAc)_2_/dppbz (10 mol%) and LiO*t*Bu (2.0 equiv.) in 1,4-dioxane at room temperature for 18 h afforded the desired methoxyborated product 3a in 11% yield (entry 1). The yield was low, but the exclusive *syn*-stereochemistry was observed, thus suggesting the operation of two-electron redox system. This intriguing result prompted us to examine the effects of bidentate dppbz ligands^8^ in more detail. In the cases using *para*-substituted dppbz derivatives, almost negligible effects on the reaction efficacy were observed, regardless of electronic nature of substituents (entries 2 and 3). On the other hand, the introduction of electron-withdrawing groups at the *meta*-position significantly improved the yield of 3a with maintenance of high *syn*-selectivity (entries 4 and 5), particularly with CF_3_-dppbz proving to be optimal, while less influences were observed with the electron-donating substituents (entries 6–8). In addition, the corresponding monodentate phosphine, P[3,5-(CF_3_)_2_C_6_H_3_]_3_, provided 3a in only 2% yield (entry 9), thus suggesting that both the strong electron-withdrawing and bidentate chelating nature of CF_3_-dppbz are essential for the reaction. Investigation of other reaction parameters such as copper catalyst precursor, solvent, and reaction temperature revealed that the yield of 3a further increased to 82% (78% isolated yield) with the Cu(CH_3_CN)_4_PF_6_ catalyst and 1,4-dioxane/toluene (1 : 1, v/v) mixed solvent system at 0 °C (entry 10). Moreover, the choice of substituents at the anomeric position of acetal-based peroxide 2 was also critical. The reaction proceeded to some extent when using methyl-, butyl-, and simple phenyl-substituted peroxides (entries 11–13). On the other hand, the introduction of electron-withdrawing CF_3_ on the phenyl ring accelerated the reaction (entry 14), whereas the electron-donating substituents such as *tert*-butyl and methoxy groups were detrimental (entries 15 and 16). Additionally noteworthy is the indispensable role of LiO*t*Bu (entry 17). More detailed results in optimization studies are involved in the SI.

**Table 1:** Optimization studies for copper-catalyzed stereospecific methoxyboration of (*E*)-1a with pinB–Bpin and acetal-based peroxides 2^a^

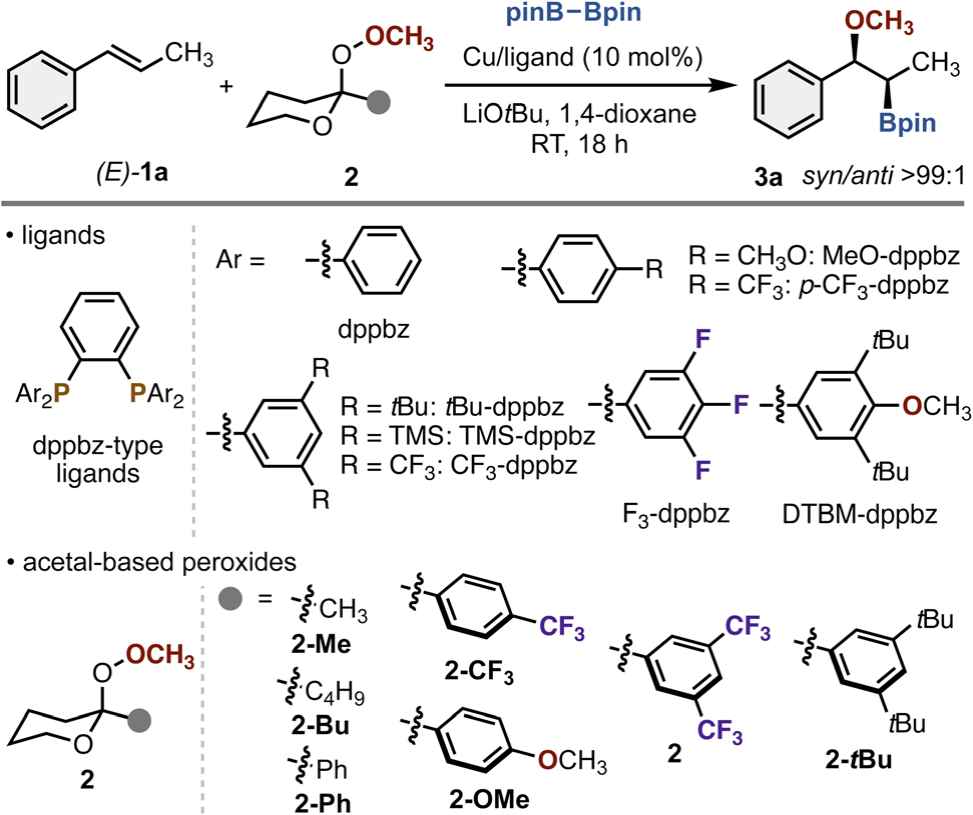			
Entry	2	Cu/ligand	Yield^b^ (%)
1	2	Cu(OAc)_2_/dppbz	11
2	2	Cu(OAc)_2_/MeO-dppbz	8
3	2	Cu(OAc)_2_/*p*-CF_3_-dppbz	8
4	2	Cu(OAc)_2_/CF_3_-dppbz	28
5	2	Cu(OAc)_2_/F_3_-dppbz	26
6	2	Cu(OAc)_2_/*t*Bu-dppbz	15
7	2	Cu(OAc)_2_/TMS-dppbz	4
8	2	Cu(OAc)_2_/DTBM-dppbz	9
9^c^	2	Cu(OAc)_2_/P[3,5-(CF_3_)_2_C_6_H_3_]_3_	2
10^d^	2	Cu(CH_3_CN)_4_PF_6_/CF_3_-dppbz	82 (78)
11^d^	2-Me	Cu(CH_3_CN)_4_PF_6_/CF_3_-dppbz	49
12^d^	2-Bu	Cu(CH_3_CN)_4_PF_6_/CF_3_-dppbz	53
13^d^	2-Ph	Cu(CH_3_CN)_4_PF_6_/CF_3_-dppbz	49
14^d^	2-CF_3_	Cu(CH_3_CN)_4_PF_6_/CF_3_-dppbz	63
15^d^	2-OMe	Cu(CH_3_CN)_4_PF_6_/CF_3_-dppbz	17
16^d^	2-*t*Bu	Cu(CH_3_CN)_4_PF_6_/CF_3_-dppbz	28
17^d^^,^^e^	2	Cu(CH_3_CN)_4_PF_6_/CF_3_-dppbz	0

a Conditions: (*E*)-1a (0.10 mmol), pinB–Bpin (0.25 mmol), 2 (0.15 mmol), Cu (0.010 mmol), ligand (0.010 mmol), LiO*t*Bu (0.20 mmol), 1,4-dioxane (0.80 mL), RT, 18 h, N_2_.

b Estimated by ^1^H NMR. Isolated yield is given in parentheses.

c With 0.020 mmol of ligand.

d In 1,4-dioxane (0.40 mL) and toluene (0.40 mL) at 0 °C.

e Without LiO*t*Bu.

**Scheme 2:**
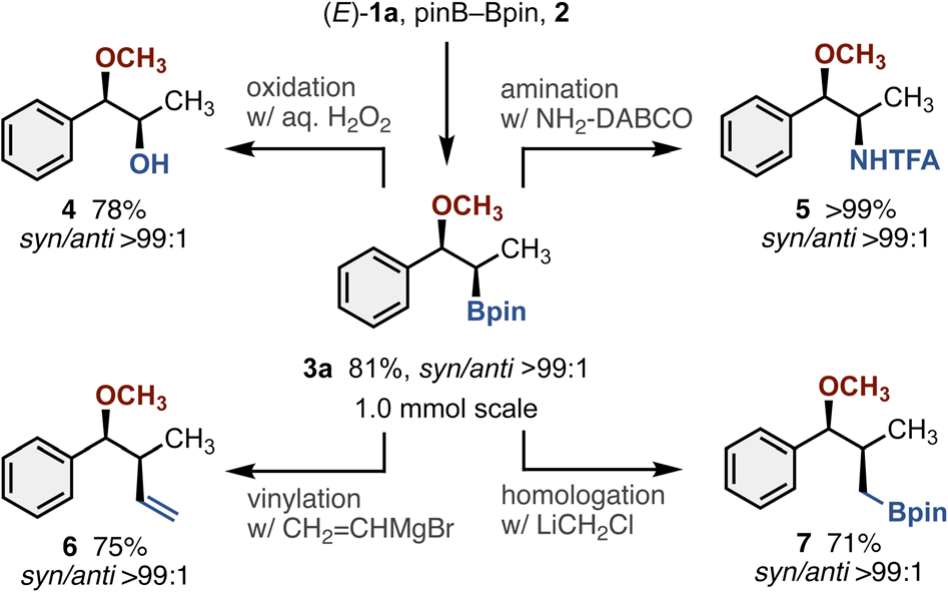
Stereospecific post functionalizations of Bpin moiety in 3a. See the SI for detailed conditions of each reaction.

The reaction of (*E*)-1a could also be performed on a 1.0 mmol scale without any difficulties, thus indicating good reliability and reproducibility of the reaction (Scheme 2). Moreover, the post functionalizations of Bpin moiety in 3a were successfully conducted under the established conditions: oxidation (4), amination (5),^9^ vinylation (6),^10^ and homologation (7)^11^ all proceeded smoothly to form the corresponding functionalized alkyl ethers with high stereochemical fidelity. Thus, the methoxyborated product 3a is a valuable building block for the stereochemically defined, functionality-rich alkyl ethers.

**Scheme 3:**
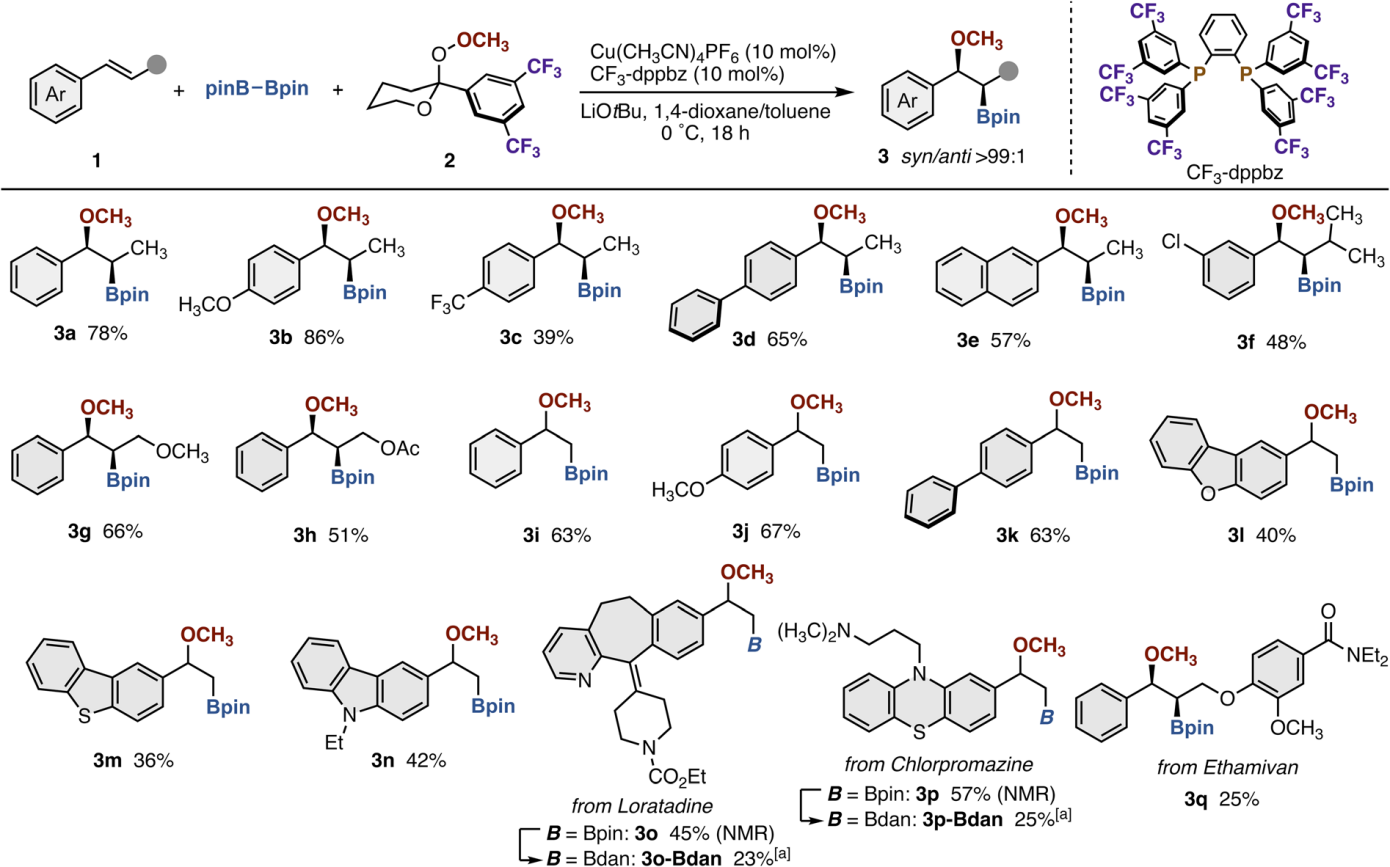
Scope of styrene derivatives in copper-catalyzed methoxyboration. Conditions: 1 (0.10–0.20 mmol), pinB–Bpin (2.5 equiv.), 2 (1.5 equiv.), Cu(CH_3_CN)_4_PF_6_ (10 mol%), CF_3_-dppbz (10 mol%), LiO*t*Bu (2.0 equiv.), 1,4-dioxane/toluene (1 : 1, v/v), 0 °C, 18 h, N_2_. Isolated yields are shown. ^*a*^Isolated in a Bdan form after transesterification of crude material with H_2_Bdan. See the SI for detailed conditions.

With conditions of entry 10 in Table 1, we next examined the scope of styrene derivatives (Scheme 3). In addition to the model substrate (*E*)-1a, both electron-rich and -deficient (*E*)-β-methylstyrenes participated in the reaction to form the corresponding 3b and 3c with high *syn*-selectivity, although the yield of 3c was relatively moderate because of poor stability under reaction conditions.^5^ More π-extended substrates such as biphenyl (3d) and naphthalene (3e) were also applicable. It should be noted that more sterically demanding β-isopropylstyrene (3f) as well as the cinnamyl ether (3g) and ester (3h) could also be used without erosion of stereospecificity. The reaction of nonsubstituted vinylarenes also proceeded well (3i–k). The copper catalysis was also compatible with heteroaromatics including dibenzofuran (3l), dibenzothiophene (3m), and carbazole (3n). Furthermore, the reaction accommodated the complex vinylarenes derived from the antihistamine medicine loratadine (3o) and the antipsychotics chlorpromazine (3p), where the methoxyboration itself proceeded with acceptable efficiency but the products were unstable in Bpin forms. Thus, purification was implemented after the transesterification into the Bdan form (dan = 1,8-diaminonaphthalene; 3o-Bdan and 3p-Bdan).^12^ The cinnamyl ether prepared from respiratory stimulant ethamivan could also be transformed to the corresponding methoxyborated product 3q with high *syn*-selectivity.

**Scheme 4:**
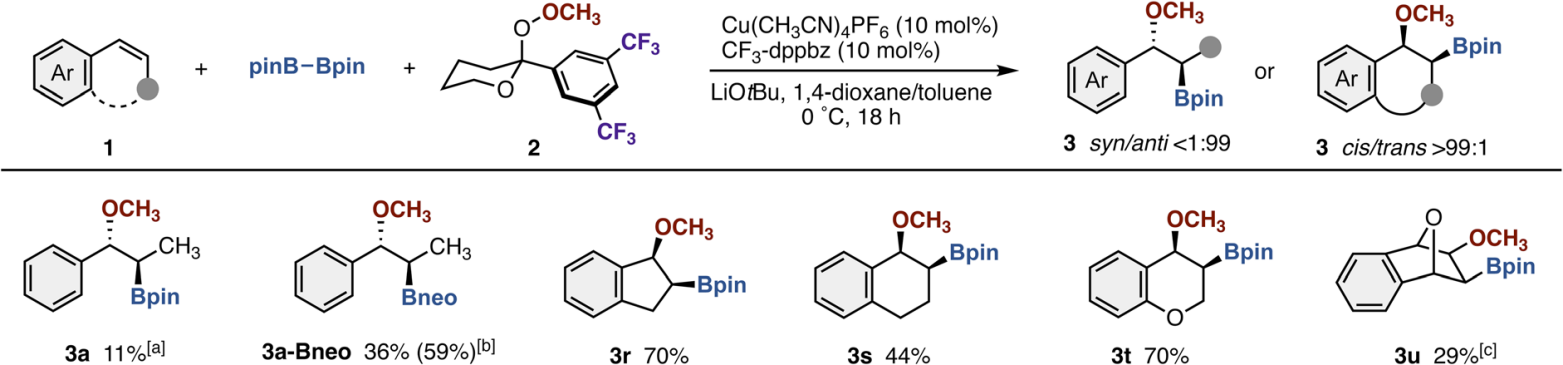
Scope of (*Z*)-styrene derivatives in copper-catalyzed methoxyboration. Conditions: 1 (0.10–0.20 mmol), pinB–Bpin (2.5 equiv.), 2 (1.5 equiv.), Cu(CH_3_CN)_4_PF_6_ (10 mol%), CF_3_-dppbz (10 mol%), LiO*t*Bu (2.0 equiv.), 1,4-dioxane/toluene (1 : 1, v/v), 0 °C, 18 h, N_2_. Isolated yields are shown. ^*a*1^H NMR yield. ^*b*^With neoB–Bneo (4.0 equiv.), Cu(CH_3_CN)_4_PF_6_ (20 mol%), CF_3_-dppbz (20 mol%), and LiO*t*Bu (4.0 equiv.). ^1^H NMR yield is in parentheses. ^*c*^In toluene.

To investigate the stereospecificity of the copper catalysis, we then attempted to apply (*Z*)-1a under otherwise identical conditions (Scheme 4). Gratifyingly, the corresponding *anti*-3a was formed exclusively albeit with a low yield (11% NMR yield). After additional optimization studies (see the SI for details), the use of bis(neopentylglycolato)diboron (neoB–Bneo) instead of pinB–Bpin increased the yield to 59% NMR yield with maintenance of high *anti*-stereochemistry (3a-Bneo; 36% isolated yield). The reaction of cyclic alkenes such as indene and 1,2-dihydronaphthalene also occurred stereospecifically to deliver the corresponding *cis*-mehoxyborated products 3r and 3s in 70% and 44% yields, respectively. 2*H*-Chromene was also viable, and the functionality-rich chromane 3t was obtained in a good yield with high *cis*-selectivity. Notably, the oxabicyclic alkene also took part in the reaction, and the corresponding *exo*-adduct 3u was exclusively produced. These outcome clearly indicates the *syn*-addition of methoxy and boryl groups across alkene π-bond under copper catalysis, which is strong support for the stereospecific two-electron redox process with the engineered acetal-based peroxide 2 rather than previous one-electron redox process with TEMPO.^4,5^

**Scheme 5:**
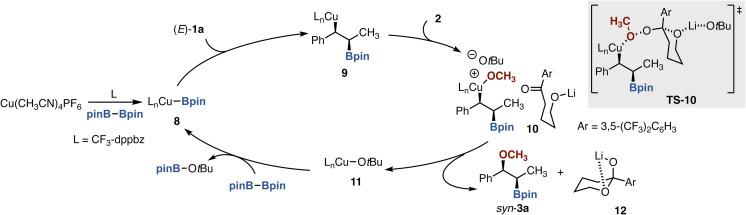
Plausible reaction mechanism.

On the basis of the aforementioned findings and literature information, we propose the reaction mechanism of the methoxyboration of (*E*)-1a with pinB–Bpin and 2 as follows (Scheme 5). The catalytically active L_*n*_Cu–Bpin species 8 is initially generated from Cu(CH_3_CN)_4_PF_6_, CF_3_-dppbz (L), and pinB–Bpin *via* ligand exchange and σ-bond metathesis. Subsequent regioselective and *syn*-selective insertion of (*E*)-1a into the Cu–B bond (8 to 9)^13^ is followed by oxidative addition with acetal-based peroxide 2, which is facilitated by the coordination of the acetal oxygen in 2 to Li cation of LiO*t*Bu (TS-10), to produce the Cu(iii) intermediate 10. Final stereoretentive reductive elimination furnishes 3a with observed *syn*-stereochemistry.^7*a*^ The concurrently formed L_*n*_Cu–O*t*Bu 11 enters the next catalytic cycle by the σ-bond metathesis with pinB–Bpin. The hemiacetal fragment and its ring-opening form arising from the released Li alkoxide 12 were actually recovered almost quantitatively (see the SI). The two-electron redox oxidative addition/reductive elimination pathway can reasonably explain the experimentally observed stereospecificity of the reaction.^14,15^

**Scheme 6:**
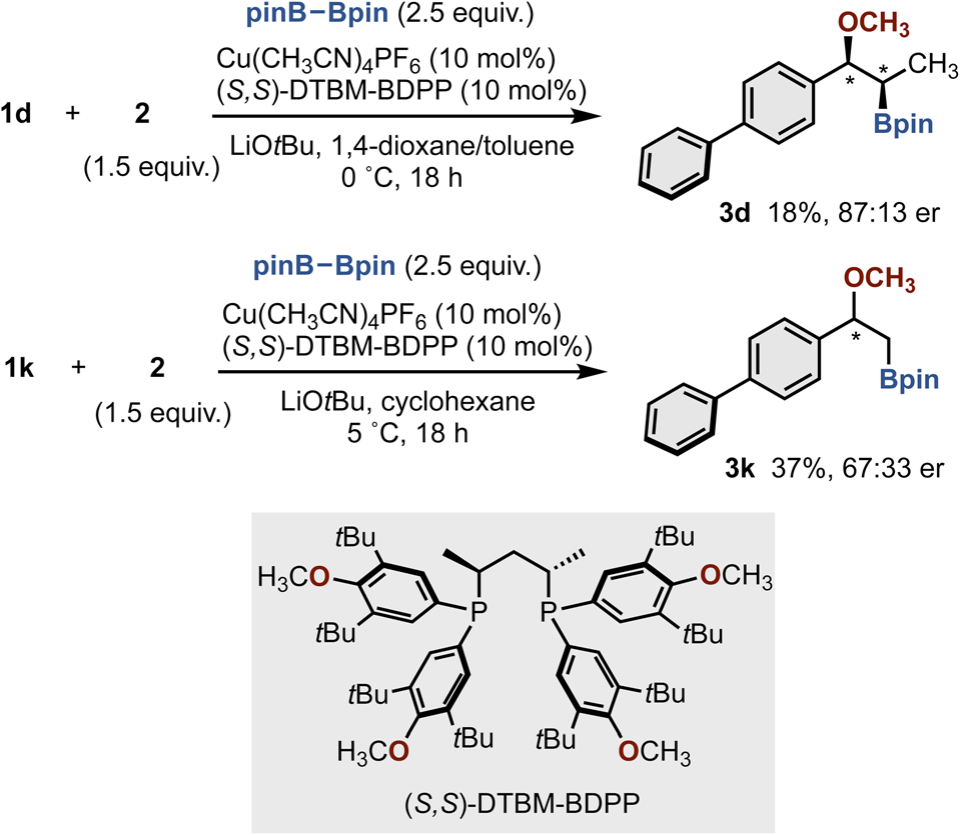
Preliminary results of catalytic asymmetric methoxyboration with (*S*,*S*)-DTBM-BDPP ligand.

Finally, we applied this protocol to the catalytic asymmetric synthesis by using an appropriate chiral ligand (Scheme 6). While still preliminary, the moderate enantioinduction (3d, 18%, 87 : 13 er) was observed in the presence of (*S*,*S*)-DTBM-BDPP. The reaction of terminal alkene 1k instead of 1d increased the yield but with lower enantioselectivity (3k, 37%, 67 : 33 er). Further attempts to improve both the yield and enantioselectivity are currently underway.

**Scheme 7:**
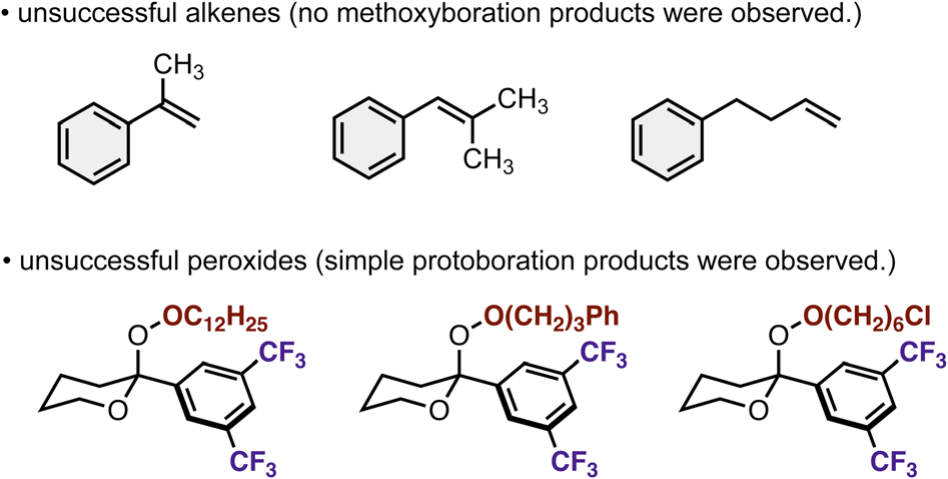
Unsuccessful alkenes and peroxides under current conditions.

Another current limitation is the narrow scope of alkenes and acetal-based peroxides (Scheme 7). Attempts to apply α-methylstyrene, β,β-dimethylstyrene, and simple aliphatic terminal alkene remained unsuccessful. We also tested alkyl peroxides other than methyl peroxide 2, but formal protoboration exclusively occurred because of increasing steric hinderances. Additional optimizations of catalysts and design of peroxides are necessary for overcoming the aforementioned issues.

## Conclusions

We have developed an oxygen-umpolung-enabled, copper-catalyzed regioselective and stereospecific methoxyboration of styrenes, delivering *syn*- and *anti*-oxyborated products from (*E*)- and (*Z*)-styrenes, respectively. The key to success lies with a CF_3_-substituted acetal-based peroxide, which has unique two-electron redox character rather than the previously dominant one-electron redox nature associated with TEMPO. The newly developed copper catalysis combined with the designed acetal-based peroxide accesses the stereochemically defined organoboron compounds with oxygenated functionality from readily available and relatively simple hydrocarbon materials. Moreover, the obtained methoxyborated products can be valuable platforms for more complicated alkyl ethers *via* stereospecific post-functionalizations of boron moiety. Given the ubiquity of ether in bioactive molecules, natural products, and functional materials, the present findings will find wide applications in synthetic organic chemistry. Extension of oxygen umpolung concept beyond coper catalysis is ongoing in our laboratory.

## Author contributions

K. H. conceived the idea. K. F. and S. N. performed all experiments. K. H. supervised the project and wrote the manuscript. All the authors discussed the results and commented on the manuscript.

## Conflicts of interest

There are no conflicts to declare.

## Supplementary Material

SC-016-D5SC07347B-s001

## Data Availability

All experimental procedures and spectroscopic data can be found in the supplementary information (SI). Supplementary information is available. See DOI: https://doi.org/10.1039/d5sc07347b.
