# Supplementary material for: Copper-catalyzed stereospecific methoxyboration of styrenes enabled by oxygen umpolung with acetal-based peroxides
Source: Chem Sci. 2025 Oct 10;16(45):21548–53. doi: 10.1039/d5sc07347b (PMC12529083; doi:10.1039/d5sc07347b)
Supplement: SC-016-D5SC07347B-s001 [file SC-016-D5SC07347B-s001.pdf]

Supplementary Information (SI) for Chemical Science.
This journal is © The Royal Society of Chemistry 2025

Supplementary Information

Copper-catalyzed stereospecific methoxyboration of styrenes enabled by oxygen umpolung with acetal-based peroxides

Kyosuke Fujiwara,[†] Shogo Nakamura,[†] and Koji Hirano*,[†,‡]

[†]*Department of Applied Chemistry, Graduate School of Engineering, Osaka University, Suita, Osaka 565-0871, Japan*

[‡]*Innovative Catalysis Science Division, Institute for Open and Transdisciplinary Research Initiatives (ICS-OTRI), Osaka University, Suita, Osaka 565-0871, Japan*

E-mail: k_hirano@chem.eng.osaka-u.ac.jp (K.H.)

Contents

**Instrumentation and Chemicals**	S2–S3
**Experimental Procedures and Characterization Data for Products**	S4–S23
**Stereochemical Assignment**	S24
**Detailed Optimization Studies**	S25–S28
**Recovery and Recycle of Hemiacetal Fragment**	S29
**Effect of Radical Trapping Reagent**	S30
**Unsuccessful Substrates**	S31
**Attempts of Asymmetric Catalysis**	S32
**Chiral HPLC Charts of Enantioenriched Product**	S33–S34
**Copies of NMR Spectra**	S35–S86
**References**	S87

Instrumentation and Chemicals

^{1}H, ^{13}C{^{1}H}, ^{19}F{^{1}H}. and ^{11}B NMR spectra were recorded at 400 MHz, 100 MHz, 376, and 128 MHz, respectively, for $CDCl_3$ or CD_2Cl_2 solutions. HRMS data were obtained by APCI and ESI using TOF. TLC analyses were performed on commercial glass plates bearing a 0.25 mm layer of Merck silica gel 60F$_{254}$. Silica gel (60 N, spherical neutral, Kanto Chemical Co.) was usually used for column chromatography. Gel permeation chromatography (GPC) was performed by LC-20AR (pump, SHIMADZU, 7.5 mL/min $CHCl_3$ or ethyl acetate) and SPD-20A (UV detector, SHIMADZU, 254 nm) with two in-line YMC-GPC T2000 (20 x 600 mm, particle size: 10 μm) (preparative columns, YMC). Unless otherwise noted, materials obtained from commercial suppliers were used without further purification. $Cu(CH_3CN)_2PF_6$, pinB–Bpin, and neoB–Bneo were obtained from TCI. Anhydrous 1,4-dioxane and toluene were purchased from FUJIFILM Wako Chemical Co. and directly used as received. CF_3-dppbz was synthesized according to the literature method.[S1] Styrene derivatives **1a**–**b**, **h**–**k**, **r**–**t** and oxabicyclic alkene **1u** were commercially available. *β*-Substituted styrenes **1c**–**e** and vinylheteroarenes **1l**–**n** were synthesized by the Witting reaction of the corresponding aldehydes. Cinnamyl methyl ether (**1g**) was prepared by the methylation of cinnamyl alcohol with NaH and MeI. The sterically hindered *i*-Pr-substituted **1f** was synthesized by the BF_3-mediated Aldol-Grob reaction.[S2] The complex styrenes **1o**–**q** were prepared according to the literature methods.[S3] All acetal-based peroxides **2** were prepared by our previously reported methods.[S4] Unless otherwise noted, all reactions were performed under nitrogen atmosphere.

Caution: All peroxides are potentially explosive. Although we did not observe any explosion and dangerous exothermic decomposition, all experiments should be performed with special cares and suitable safeguards. In addition, we confirmed that solid peroxide 2 was not detonated by a hammer blow, but contacts with heat and metallic materials (e.g. metallic spatulas) should also be avoided.

Experimental Procedures and Characterization Data for Products

Copper-Catalyzed Methoxyboration of Styrenes with Dboron and Acetal-Based Peroxide: General Procedure A

$Cu(CH_3CN)_4PF_6$ (10 mol%), CF_3-dppbz (10 mol%), and LiO*t*Bu (2.0 equiv) were placed in a 20 mL-Schlenk flask, which was filled with nitrogen by the standard Schlenk technique. 1,4-Dioxane and toluene (1/1, v/v) were added, and the solution was stirred at room temperature for 15 min. pinB–Bpin (2.5 equiv) was then added, and after additional 5 min, the solution was cooled to 0 ˚C (cooling block). Finally, styrene **1** (1.0 equiv) and 2-(3,5-bis(trifluoromethyl)phenyl)-2-(methylperoxy)tetrahydro-2*H*-pyran (**2**; 1.5 equiv) were added, and the resulting solution was stirred for 18 h at 0 ˚C. The solution was filtered through a short pad of Na_2SO_4 and activated alumina, and the filtrate was evaporated under reduced pressure. The residue was purified by column chromatography on silica gel and/or GPC to give the corresponding methoxyborated product **3**.

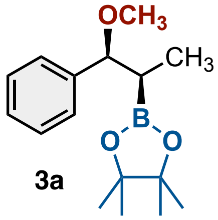

3a

2-((1*S,2*R**)-1-Methoxy-1-phenylpropan-2-yl)-4,4,5,5-tetramethyl-1,3,2-dioxaborolane (3a)**

The reaction was performed with $Cu(CH_3CN)_4PF_6$ (3.7 mg, 0.010 mmol, 10 mol%), CF_3-dppbz (9.9 mg, 0.010 mmol, 10 mol%), LiO*t*Bu (16 mg, 0.20 mmol, 2.0 equiv), (*E*)-**1a** (12 mg, 0.10 mmol, 1.0 equiv), **2** (52 mg, 0.15 mmol, 1.5 equiv), pinB–Bpin (64 mg, 0.25 mmol, 2.5 equiv), 1,4-dioxane (0.40 mL), and toluene (0.40 mL) according to **General Procedure A**. It was purified by silica gel column chromatography with hexane/ethyl acetate (10/1, v/v): 21 mg (78%, 0.1 mmol scale); 224 mg (81%, 1.0 mmol scale) ; colorless oil; ^1H NMR ($CDCl_3$, 400 MHz): δ 7.35-7.24 (m, 5H), 4.05 (d, *J* = 10.2 Hz, 1H), 3.15 (s, 3H), 1.46 (dq, *J* = 10.2, 7.4 Hz, 1H), 1.30 (s, 6H), 1.27 (s, 6H), 0.73 (d, *J* = 7.4 Hz, 3H); $^{13}C\{^1H\}$ NMR ($CDCl_3$, 100 MHz): δ 141.6, 128.1, 127.5, 127.4, 87.4, 83.1, 56.5, 24.9, 24.4, 11.3 (The carbon signal bound to boron was not observed because of quadrupolar relaxation.); ^{11}B NMR ($CDCl_3$, 128 MHz): δ 34.33; HRMS (ESI) m/z $(M+Na)^+$ calcd for $C_{16}H_{25}BNaO_3$: 299.1789, found: 299.1779.

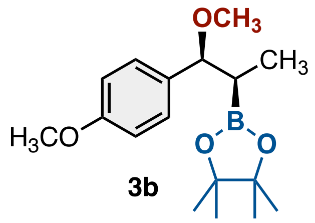

3b

2-((1*S,2*R**)-1-Methoxy-1-(4-methoxyphenyl)propan-2-yl)-4,4,5,5-tetramethyl-1,3,2-dioxaborolane (3b)**

The reaction was performed with $Cu(CH_3CN)_4PF_6$ (3.7 mg, 0.010 mmol, 10 mol%), CF_3-dppbz (9.9 mg, 0.010 mmol, 10 mol%), LiO*t*Bu (16 mg, 0.20 mmol, 2.0 equiv), **1b** (15 mg, 0.10 mmol, 1.0 equiv), **2** (52 mg, 0.15 mmol, 1.5 equiv), pinB–Bpin (64 mg, 0.25 mmol, 2.5 equiv), 1,4-dioxane (0.40 mL), and toluene (0.40 mL) according to **General Procedure A**. It was purified by silica gel column chromatography with hexane/ethyl acetate (10/1, v/v): 26 mg (86%, 0.1 mmol scale); colorless oil; ^{1}H NMR ($CDCl_3$, 400 MHz): δ 7.22 (d, *J* = 8.6 Hz, 2H), 6.87 (d, *J* = 8.7 Hz, 2H), 4.00 (d, *J* = 10.2 Hz, 1H), 3.81 (s, 3H), 3.12 (s, 3H), 1.44 (dq, *J* = 10.2, 7.4 Hz, 1H), 1.29 (s, 6H), 1.27 (s, 6H), 0.72 (d, *J* = 7.4 Hz, 3H); $^{13}C\{^1H\}$ NMR ($CDCl_3$, 100 MHz): δ 159.0 135.6, 128.6, 113.5, 86.9, 83.1, 56.3, 55.2, 24.9, 24.4, 11.3 (The carbon signal bound to boron was not observed because of quadrupolar relaxation.); ^{11}B NMR ($CDCl_3$, 128 MHz): δ 34.24; HRMS (ESI) m/z $(M+Na)^+$ calcd for $C_{17}H_{27}BNaO_4$: 329.1895, found: 329.1885.

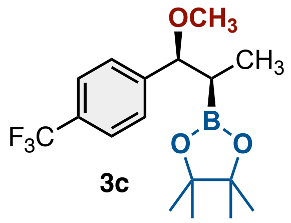

3c

2-((1*S,2*R**)-1-Methoxy-1-(4-(trifluoromethyl)phenyl)propan-2-yl)-4,4,5,5-tetramethyl-1,3,2-dioxaborolane (3c)**

The reaction was performed with $Cu(CH_3CN)_4PF_6$ (3.7 mg, 0.010 mmol, 10 mol%), CF_3-dppbz (9.9 mg, 0.010 mmol, 10 mol%), LiO*t*Bu (16 mg, 0.20 mmol, 2.0 equiv), **1c** (19 mg, 0.10 mmol, 1.0 equiv), **2** (52 mg, 0.15 mmol, 1.5 equiv), pinB–Bpin (64 mg, 0.25 mmol, 2.5 equiv), 1,4-dioxane (0.40 mL), and toluene (0.40 mL) according to **General Procedure A**. It was purified by silica gel column chromatography with hexane/ethyl acetate (10/1, v/v): 13 mg (39%, 0.1 mmol scale); colorless oil; ^{1}H NMR ($CDCl_3$, 400 MHz): δ 7.59 (d, *J* = 8.0 Hz, 2H), 7.42 (d, *J* = 8.7 Hz, 2H), 4.12 (d, *J* = 9.9 Hz, 1H),

3.16 (s, 3H), 1.44 (dq, J = 9.9, 7.5 Hz, 1H), 1.29 (s, 6H), 1.27 (s, 6H), 0.74 (d, J = 7.4 Hz, 3H); $^{13}C\{^1H\}$ NMR ($CDCl_3$, 100 MHz): δ 145.9, 129.7 (q, J = 31.9 Hz), 127.6, 125.1 (q, J = 3.7 Hz), 124.2 (q, J = 270.2 Hz), 86.7, 83.3, 56.8, 24.9, 24.4, 11.1 (The carbon signal bound to boron was not observed because of quadrupolar relaxation.); $^{19}F\{^1H\}$ NMR ($CDCl_3$, 376 MHz): δ -62.40; ^{11}B NMR ($CDCl_3$, 128 MHz): δ 34.33; HRMS (ESI) m/z $(M+Na)^+$ calcd for $C_{17}H_{24}BF_3NaO_3$: 367.1663, found: 367.1646.

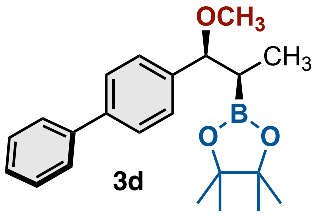

2-((1*S,2*R**)-1-([1,1'-Biphenyl]-4-yl)-1-methoxypropan-2-yl)-4,4,5,5-tetramethyl-1,3,2-dioxaborolane (3d)**

The reaction was performed with $Cu(CH_3CN)_4PF_6$ (3.7 mg, 0.010 mmol, 10 mol%), CF_3-dppbz (9.9 mg, 0.010 mmol, 10 mol%), LiO*t*Bu (16 mg, 0.20 mmol, 2.0 equiv), **1d** (19 mg, 0.10 mmol, 1.0 equiv), **2** (52 mg, 0.15 mmol, 1.5 equiv), pinB–Bpin (64 mg, 0.25 mmol, 2.5 equiv), 1,4-dioxane (0.40 mL), and toluene (0.40 mL) according to **General Procedure A**. It was purified by silica gel column chromatography with hexane/ethyl acetate (10/1, v/v): 22 mg (63%, 0.1 mmol scale); colorless oil; ^1H NMR ($CDCl_3$, 400 MHz): δ 7.62-7.55 (m, 4H), 7.46-7.31 (m, 5H), 4.11 (d, J = 10.1 Hz, 1H), 3.19 (s, 3H), 1.50 (dq, J = 10.1, 7.4 Hz, 1H), 1.31 (s, 6H), 1.28 (s, 6H), 0.78 (d, J = 7.4 Hz, 3H); $^{13}C\{^1H\}$ NMR ($CDCl_3$, 100 MHz): δ 141.0, 140.7, 140.3, 128.7, 127.8, 127.2, 127.1, 126.9, 87.1, 83.1, 56.6, 24.9, 24.5, 11.4 (The carbon signal bound to boron was not observed because of quadrupolar relaxation.); ^{11}B NMR ($CDCl_3$, 128 MHz): δ 34.89; HRMS (ESI) m/z $(M+Na)^+$ calcd for $C_{22}H_{29}BNaO_3$: 375.2102, found: 375.2096

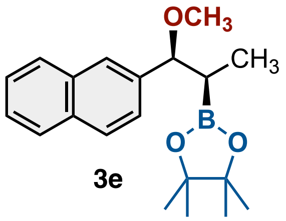

2-((1*S,2*R**)-1-Methoxy-1-(naphthalen-2-yl)propan-2-yl)-4,4,5,5-tetramethyl-1,3,2-dioxaborolane (3e)**

The reaction was performed with $Cu(CH_3CN)_4PF_6$ (3.7 mg, 0.010 mmol, 10 mol%), CF_3-dppbz (9.9 mg, 0.010 mmol, 10 mol%), LiO*t*Bu (16 mg, 0.20 mmol, 2.0 equiv), **1e** (17 mg, 0.10 mmol, 1.0 equiv), **2** (52 mg, 0.15 mmol, 1.5 equiv), pinB–Bpin (64 mg, 0.25 mmol, 2.5 equiv), 1,4-dioxane (0.40 mL), and toluene (0.40 mL) according to **General Procedure A**. It was purified by silica gel column chromatography with hexane/ethyl acetate (10/1, v/v): 19 mg (57%, 0.1 mmol scale); colorless oil; ^{1}H NMR ($CDCl_3$, 400 MHz): δ 7.84-7.81 (m, 3H), 7.71 (s, 1H), 7.51-7.44 (m, 3H), 4.23 (d, *J* = 10.1 Hz, 1H), 3.18 (s, 3H), 1.61-1.53 (m, 1H), 1.31 (s, 6H), 1.29 (s, 6H), 0.75 (d, *J* = 7.4 Hz, 3H); ^{13}C{^{1}H} NMR ($CDCl_3$, 100 MHz): δ 139.0, 133.2, 133.0, 128.2, 127.9, 127.7, 127.0, 125.9, 125.7, 124.8, 87.4, 83.1, 56.5, 24.9, 24.4, 11.3 (The carbon signal bound to boron was not observed because of quadrupolar relaxation.); ^{11}B NMR ($CDCl_3$, 128 MHz): δ 33.90; HRMS (ESI) m/z $(M+H)^+$ calcd for $C_{20}H_{27}BNaO_3$: 349.1946, found: 349.1922.

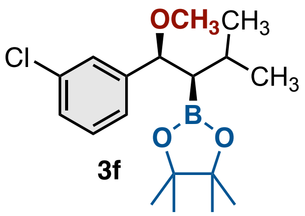

3f

2-((1*S,2*R**)-1-(3-Chlorophenyl)-1-methoxy-3-methylbutan-2-yl)-4,4,5,5-tetramethyl-1,3,2-dioxaborolane (3f)**

The reaction was performed with $Cu(CH_3CN)_4PF_6$ (3.7 mg, 0.010 mmol, 10 mol%), CF_3-dppbz (9.9 mg, 0.010 mmol, 10 mol%), LiO*t*Bu (16 mg, 0.20 mmol, 2.0 equiv), **1f** (18 mg, 0.10 mmol, 1.0 equiv), **2** (52 mg, 0.15 mmol, 1.5 equiv), pinB–Bpin (64 mg, 0.25 mmol, 2.5 equiv), 1,4-dioxane (0.40 mL), and toluene (0.40 mL) according to **General Procedure A**. It was purified by silica gel column chromatography with hexane/ethyl acetate (10/1, v/v) and GPC ($CHCl_3$): 16 mg (48%, 0.1 mmol scale); white solid; mp 54.6-53.7 ℃; ^{1}H NMR ($CDCl_3$, 400 MHz): δ 7.32 (s, 1H), 7.28-7.17 (m, 3H), 4.19 (d, *J* = 10.2 Hz, 1H), 3.13 (s, 3H), 1.42-1.304 (m, 2H), 1.281 (s, 6H), 1.278 (s, 6H), 0.94 (d, *J* = 6.7 Hz, 3H), 0.83 (d, *J* = 6.7 Hz, 3H); ^{13}C{^{1}H} NMR ($CDCl_3$, 100 MHz): δ 144.5 134.2, 129.5, 127.7, 127.3, 125.5, 84.3, 83.2, 56.6, 26.1, 25.1, 24.5, 23.7, 20.2 (The carbon signal bound to boron was not observed because of quadrupolar relaxation.); ^{11}B NMR ($CDCl_3$, 128 MHz): δ 34.06; HRMS (ESI) m/z $(M+H)^+$ calcd for $C_{18}H_{28}BClNaO_3$: 361.1712, found: 361.1700.

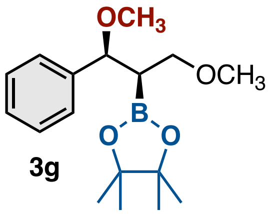

3g

2-((1*S,2*R**)-1,3-Dimethoxy-1-phenylpropan-2-yl)-4,4,5,5-tetramethyl-1,3,2-dioxaborolane (3g)**

The reaction was performed with $Cu(CH_3CN)_4PF_6$ (3.7 mg, 0.010 mmol, 10 mol%), CF_3-dppbz (9.9 mg, 0.010 mmol, 10 mol%), LiO*t*Bu (16 mg, 0.20 mmol, 2.0 equiv), **1g** (15 mg, 0.10 mmol, 1.0 equiv), **2** (52 mg, 0.15 mmol, 1.5 equiv), pinB–Bpin (64 mg, 0.25 mmol, 2.5 equiv), 1,4-dioxane (0.40 mL), and toluene (0.40 mL) according to **General Procedure A**. It was purified by silica gel column chromatography with hexane/ethyl acetate (10/1, v/v): 20 mg (66%, 0.1 mmol scale); colorless oil; ^{1}H NMR ($CDCl_3$, 400 MHz): δ 7.36-7.25 (m, 5H), 4.25 (d, J = 10.0 Hz, 1H), 3.23 (dd, J = 8.6, 8.6 Hz, 1H), 3.20 (s, 3H), 3.16 (s, 3H), 3.14 (dd, J = 8.7, 6.1 Hz, 1H), 1.83-1.77 (m, 1H), 1.29 (s, 6H), 1.28 (s, 6H); $^{13}C\{^{1}H\}$ NMR ($CDCl_3$, 100 MHz): δ 141.8, 128.3, 127.6, 127.0, 83.34, 83.32, 71.3, 58.7, 56.6, 24.8, 24.4 (The carbon signal bound to boron was not observed because of quadrupolar relaxation.); ^{11}B NMR ($CDCl_3$, 128 MHz): δ 33.45; HRMS (ESI) m/z $(M+Na)^+$ calcd for $C_{17}H_{27}BNaO_4$: 329.1895, found: 329.1884.

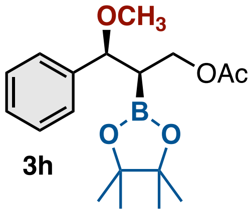

3h

(2*R,3*S**)-3-Methoxy-3-phenyl-2-(4,4,5,5-tetramethyl-1,3,2-dioxaborolan-2-yl)propyl acetate (3h)**

The reaction was performed with $Cu(CH_3CN)_4PF_6$ (3.7 mg, 0.010 mmol, 10 mol%), CF_3-dppbz (9.9 mg, 0.010 mmol, 10 mol%), LiO*t*Bu (16 mg, 0.20 mmol, 2.0 equiv), **1h** (18 mg, 0.10 mmol, 1.0 equiv), **2** (52 mg, 0.15 mmol, 1.5 equiv), pinB–Bpin (64 mg, 0.25 mmol, 2.5 equiv), 1,4-dioxane (0.40 mL), and toluene (0.40 mL) according to **General Procedure A**. It was purified by silica gel column chromatography with hexane/ethyl acetate (10/1, v/v): 17 mg (51%, 0.1 mmol scale); colorless oil; ^{1}H NMR ($CDCl_3$, 400 MHz): δ 7.36-7.25 (m, 5H), 4.24 (d, J = 9.8 Hz, 1H), 3.99 (dd, J = 10.8, 6.7 Hz, 1H), 3.90 (dd, J = 10.7, 7.9 Hz, 1H), 3.15 (s, 3H), 1.92 (s, 3H), 1.90-1.85 (m, 1H), 1.280 (s, 6H), 1.276 (s, 6H); $^{13}C\{^{1}H\}$ NMR ($CDCl_3$, 100 MHz): δ 170.9, 141.1, 128.4, 127.9, 127.1, 83.53, 83.46, 63.1, 56.5,

24.8, 24.5, 20.9 (The carbon signal bound to boron was not observed because of quadrupolar relaxation.); ^{11}B NMR ($CDCl_3$, 128 MHz): δ 33.47; HRMS (ESI) m/z $(M+H)^+$ calcd for $C_{18}H_{27}BNaO_5$: 357.1844, found: 357.1818.

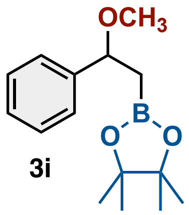

3i

2-(2-Methoxy-2-phenylethyl)-4,4,5,5-tetramethyl-1,3,2-dioxaborolane (3i)

The reaction was performed with $Cu(CH_3CN)_4PF_6$ (7.4 mg, 0.020 mmol, 10 mol%), CF_3-dppbz (20 mg, 0.020 mmol, 10 mol%), LiO*t*Bu (32 mg, 0.40 mmol, 2.0 equiv), **1i** (21 mg, 0.20 mmol, 1.0 equiv), **2** (104 mg, 0.30 mmol, 1.5 equiv), pinB–Bpin (128 mg, 0.50 mmol, 2.5 equiv), 1,4-dioxane (0.80 mL), and toluene (0.80 mL) according to **General Procedure A**. It was purified by silica gel column chromatography with hexane/ethyl acetate (40/1, v/v) then GPC ($CHCl_3$): 32 mg (63%, 0.20 mmol scale); pale yellow oil; ^{1}H NMR (400 MHz, $CDCl_3$): δ 7.35-7.29 (m, 4H), δ 7.25-7.22 (m, 1H), 4.40 (t, J = 7.7 Hz, 1H), 3.18 (s, 3H), 1.48 (dd, J = 14.9, 7.2 Hz, 1H), 1.30 (dd, J = 14.9, 7.3 Hz, 1H), 1.17 (s, 6H), 1.16 (s, 6H). $^{13}C\{^1H\}$ NMR (100 MHz, $CDCl_3$): δ 143.5, 128.2, 127.4, 126.6, 83.2, 81.3, 56.4, 24.8, 24.6 (The carbon signal bound to boron was not observed due to quadrupolar relaxation.). ^{11}B NMR (128 MHz, $CDCl_3$) : δ 33.12. HRMS (ESI) m/z $(M+Na)^+$ calcd for $C_{15}H_{23}BNaO_3$: 285.1633, found: 285.1629.

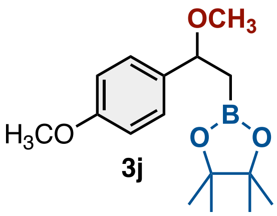

3j

2-(2-Methoxy-2-(4-methoxyphenyl)ethyl)-4,4,5,5-tetramethyl-1,3,2-dioxaborolane (3j)

The reaction was performed with $Cu(CH_3CN)_4PF_6$ (7.4 mg, 0.020 mmol, 10 mol%), CF_3-dppbz (20 mg, 0.020 mmol, 10 mol%), LiO*t*Bu (32 mg, 0.40 mmol, 2.0 equiv), **1j** (27 mg, 0.20 mmol, 1.0 equiv), **2** (104 mg, 0.30 mmol, 1.5 equiv), pinB–Bpin (128 mg, 0.50 mmol, 2.5 equiv), 1,4-dioxane (0.80 mL), and toluene (0.80 mL) according to **General Procedure A**. It was purified by GPC ($CHCl_3$ then ethyl acetate): 37 mg (67%, 0.20 mmol scale); pale yellow oil; ^{1}H NMR (400 MHz, $CDCl_3$): δ 7.26 (d, J = 8.6

Hz, 2H), 6.86 (d, J = 8.7 Hz, 2H), 4.35 (t, J = 7.6 Hz, 1H), 3.79 (s, 3H), 3.15 (s, 3H), 1.48 (dd, J = 14.9, 7.2 Hz, 1H), 1.29 (dd, J = 14.9, 7.3 Hz, 1H), 1.17 (s, 6H), 1.16 (s, 6H). $^{13}C\{^1H\}$ NMR (100 MHz, $CDCl_3$): δ 158.9, 135.5, 127.8, 113.6, 83.2, 80.7, 56.2, 55.3, 24.7, 24.6 (The carbon signal bound to boron was not observed due to quadrupolar relaxation.). ^{11}B NMR (128 MHz, $CDCl_3$): δ 33.27. HRMS (ESI) m/z $(M+Na)^+$ calcd for $C_{16}H_{25}BNaO_4$: 315.1738, found: 315.1738.

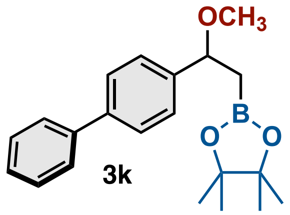

3k

2-(2-([1,1'-Biphenyl]-4-yl)-2-methoxyethyl)-4,4,5,5-tetramethyl-1,3,2-dioxaborolane (3k)

The reaction was performed with $Cu(CH_3CN)_4PF_6$ (7.4 mg, 0.020 mmol, 10 mol%), CF_3-dppbz (20 mg, 0.020 mmol, 10 mol%), LiO*t*Bu (32 mg, 0.40 mmol, 2.0 equiv), **1k** (36 mg, 0.20 mmol, 1.0 equiv), **2** (104 mg, 0.30 mmol, 1.5 equiv), pinB–Bpin (128 mg, 0.50 mmol, 2.5 equiv), 1,4-dioxane (0.80 mL), and toluene (0.80 mL) according to **General Procedure A**. It was purified by GPC ($CHCl_3$ then ethyl acetate): 43 mg (63%, 0.20 mmol scale); colorless oil; ^1H NMR (400 MHz, $CDCl_3$): δ 7.60-7.54 (m, 4H), 7.45-7.40 (m, 4H), 7.35-7.31 (m, 1H), 4.45 (t, J = 7.7 Hz, 1H), 3.23 (s, 3H), 1.51 (dd, J = 15.0, 7.2 Hz, 1H), 1.33 (dd, J = 14.9, 7.3 Hz, 1H), 1.19 (s, 6H), 1.17 (s, 6H). $^{13}C\{^1H\}$ NMR (100 MHz, $CDCl_3$): δ 142.6, 141.1, 140.3, 128.7, 127.1, 127.05 (2C), 127.01, 83.2, 81.0, 56.5, 24.8, 24.6 (The carbon signal bound to boron was not observed due to quadrupolar relaxation.). ^{11}B NMR (128 MHz, $CDCl_3$): δ 31.78. HRMS (ESI) m/z $(M+Na)^+$ calcd for $C_{21}H_{27}BNaO_3$: 361.1946, found: 361.1949.

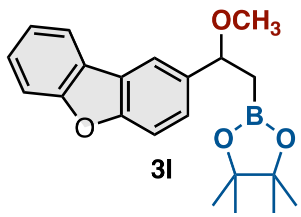

3l

2-(2-(Dibenzo[*b*,*d*]furan-2-yl)-2-methoxyethyl)-4,4,5,5-tetramethyl-1,3,2-dioxaborolane (3l)

The reaction was performed with $Cu(CH_3CN)_4PF_6$ (7.4 mg, 0.020 mmol, 10 mol%), CF_3-dppbz (20 mg, 0.020 mmol, 10 mol%), LiO*t*Bu (32 mg, 0.40 mmol, 2.0 equiv), **1l** (39 mg, 0.20 mmol, 1.0 equiv), **2** (104

mg, 0.30 mmol, 1.5 equiv), pinB–Bpin (128 mg, 0.50 mmol, 2.5 equiv), 1,4-dioxane (0.80 mL), and toluene (0.80 mL) according to **General Procedure A**. It was purified by silica gel column chromatography with hexane/ethyl acetate (40/1, v/v) then GPC ($CHCl_3$): 28 mg (40%, 0.20 mmol scale); pale yellow oil; ^{1}H NMR (400 MHz, $CDCl_3$): δ 7.94 (s, 1H), 7.93 (d, J = 10.6 Hz, 1H), 7.56-7.50 (m, 2H), 7.46-7.42 (m, 2H), 7.34 (td, J = 7.7, 0.8 Hz, 1H), 4.57 (t, J = 7.7 Hz, 1H), 3.23 (s, 3H), 1.58 (dd, J = 15.0, 7.7 Hz, 1H), 1.40 (dd, J = 15.0, 6.9 Hz, 1H), 1.15 (s, 6H), 1.13 (s, 6H). ^{13}C{^{1}H} NMR (100 MHz, $CDCl_3$): δ 156.6, 155.7, 138.2 , 127.1, 125.9, 124.3, 124.1, 122.7, 120.6, 118.7, 111.7, 111.3, 83.2, 81.3, 56.4, 24.71, 24.68 (The carbon signal bound to boron was not observed due to quadrupolar relaxation.). ^{11}B NMR (128 MHz, $CDCl_3$): δ 33.27. HRMS (ESI) m/z $(M+Na)^+$ calcd for $C_{21}H_{25}BNaO_4$: 375.1738, found: 375.1741.

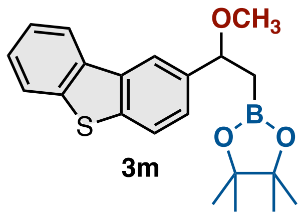

3m

2-(2-(Dibenzo[*b,d*]thiophen-2-yl)-2-methoxyethyl)-4,4,5,5-tetramethyl-1,3,2-dioxaborolane (3m)

The reaction was performed with $Cu(CH_3CN)_4PF_6$ (7.4 mg, 0.020 mmol, 10 mol%), CF_3-dppbz (20 mg, 0.020 mmol, 10 mol%), LiO*t*Bu (32 mg, 0.40 mmol, 2.0 equiv), **1m** (42 mg, 0.20 mmol, 1.0 equiv), **2** (104 mg, 0.30 mmol, 1.5 equiv), pinB–Bpin (128 mg, 0.50 mmol, 2.5 equiv), 1,4-dioxane (0.80 mL), and toluene (0.80 mL) according to **General Procedure A**. It was purified by GPC ($CHCl_3$ then ethyl acetate): 27 mg (36%, 0.20 mmol scale); colorless oil; ^{1}H NMR (400 MHz, $CDCl_3$): δ 8.16-8.14 (m, 2H), 7.86-7.82 (m, 1H), 7.80 (d, J = 8.2 Hz, 1H), 7.48-7.43 (m, 3H), 4.59 (t, J = 7.7 Hz, 1H), 3.24 (s, 3H), 1.58 (dd, J = 7.4, 14.9 Hz, 1H), 1.40 (dd, J = 7.2, 15.0 Hz, 1H), 1.17 (s, 6H), 1.15 (s, 6H). ^{13}C{^{1}H} NMR (100 MHz, $CDCl_3$): δ 140.2, 139.8, 138.4, 135.5 (2C), 126.6, 125.5, 124.4, 122.9, 122.7, 121.6, 119.7, 83.3, 81.4, 56.5, 24.75, 24.69 (The carbon signal bound to boron was not observed due to quadrupolar relaxation.). ^{11}B NMR (128 MHz, $CDCl_3$): δ 33.33. HRMS (ESI) m/z $(M+Na)^+$ calcd for $C_{21}H_{25}BNaO_3S$: 391.1510, found: 391.1504.

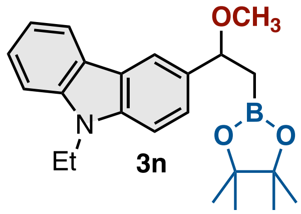

9-Ethyl-3-(1-methoxy-2-(4,4,5,5-tetramethyl-1,3,2-dioxaborolan-2-yl)ethyl)-9*H*-carbazole (3n)

The reaction was performed with $Cu(CH_3CN)_4PF_6$ (7.4 mg, 0.020 mmol, 10 mol%), CF_3-dppbz (20 mg, 0.020 mmol, 10 mol%), LiO*t*Bu (32 mg, 0.40 mmol, 2.0 equiv), **1n** (44 mg, 0.20 mmol, 1.0 equiv), **2** (104 mg, 0.30 mmol, 1.5 equiv), pinB–Bpin (128 mg, 0.50 mmol, 2.5 equiv), 1,4-dioxane (0.80 mL), and toluene (0.80 mL) according to **General Procedure A**. It was purified by GPC ($CHCl_3$ then ethyl acetate): 32 mg (42%, 0.20 mmol scale); colorless oil; ^1H NMR (400 MHz, $CDCl_3$): δ 8.07 (d, J = 7.8 Hz, 1H), 8.06 (s, 1H), 7.49-7.43 (m, 2H), 7.41-7.36 (m, 2H), 7.24-7.20 (m, 1H), 4.61 (t, J = 7.6 Hz, 1H), 4.36 (q, J = 7.4 Hz, 2H), 3.22 (s, 3H), 1.61 (dd, J = 7.3, 14.9 Hz, 1H), 1.433 (dd, J = 6.3, 15.1 Hz, 1H), 1.425 (t, J = 7.2 Hz, 3H), 1.15 (s, 6H), 1.13 (s, 6H). $^{13}C\{^{18}H\}$ NMR (100 MHz, $CDCl_3$): δ 140.2, 139.6, 133.9, 125.5, 124.4, 123.0, 122.6, 120.4, 118.8, 118.7, 108.4, 108.3, 83.1, 81.7, 56.3, 37.6, 24.71, 24.68, 13.8 (The carbon signal bound to boron was not observed due to quadrupolar relaxation.). ^{11}B NMR (128 MHz, $CDCl_3$): δ 33.40. HRMS (ESI) m/z $(M+Na)^+$ calcd for $C_{23}H_{30}BNNaO_3$: 402.2211, found: 402.2210.

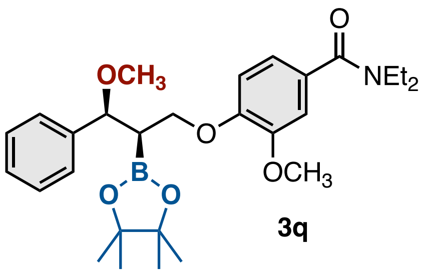

3-(2-(1-Methoxy-2-(1*H*-naphtho[1,8-*de*][1,3,2]diazaborinin-2(3*H*)-yl)ethyl)-10*H*-phenothiazin-10-yl)-*N*,*N*-dimethylpropan-1-amine (3p-Bdan)

The reaction was performed with $Cu(CH_3CN)_4PF_6$ (3.7 mg, 0.010 mmol, 10 mol%), CF_3-dppbz (9.9 mg, 0.010 mmol, 10 mol%), LiO*t*Bu (16 mg, 0.20 mmol, 2.0 equiv), **1q** (34 mg, 0.10 mmol, 1.0 equiv), **2** (52 mg, 0.15 mmol, 1.5 equiv), pinB–Bpin (64 mg, 0.25 mmol, 2.5 equiv), 1,4-dioxane (0.40 mL), and toluene (0.40 mL) according to **General Procedure A**. I was purified by silica gel column chromatography with hexane/ethyl acetate (1/1, v/v) then GPC (ethyl acetate): 13 mg (25%, 0.10 mmol scale); brown oil; ^1H NMR (400 MHz, CD_2Cl_2): δ 7.19- 7.05 (m, 5H), 6.98- 6.89 (m, 6H), 6.28 (dd, J =

7.3, 1.0 Hz, 2H), 5.99 (brs, 2H), 4.39 (t, *J* = 6.6 Hz, 1H), 3.87 (t, *J* = 6.9 Hz, 2H), 3.25 (s, 3H), 2.27 (t, *J* = 7.2 Hz, 2H), 2.03 (s, 6H), 1.87- 1.82 (m, 2H), 1.44- 1.33 (m, 2H); $^{13}C\{^1H\}$ NMR (100 MHz, CD_2Cl_2): δ 146.3, 146.2, 144.3, 142.3, 142.0, 137.1, 128.3, 128.0, 127.9, 125.7, 124.8, 123.1, 121.3, 117.9, 116.4, 114.2, 106,4, 106.2, 82.6, 57.4, 57.1, 46.0, 45.9, 25.6 (The carbon signal bound to boron was not observed due to quadrupolar relaxation.).; ^{11}B NMR (128 MHz, CD_2Cl_2): δ 31.21; HRMS (APCI) m/z $(M+H)^+$ calcd for $C_{30}H_{34}BN_4OS$: 509.2546, found: 509.2542.

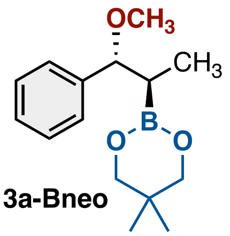

3a-Bneo

2-((1*R,2*R**)-1-Methoxy-1-phenylpropan-2-yl)-5,5-dimethyl-1,3,2-dioxaborinane (3a-Bneo)**

The reaction was performed with $Cu(CH_3CN)_4PF_6$ (7.4 mg, 0.020 mmol, 20 mol%), CF_3-dppbz (20 mg, 0.020 mmol, 20 mol%), LiO*t*Bu (32 mg, 0.40 mmol, 4.0 equiv), (*Z*)-**1a** (12 mg, 0.10 mmol, 1.0 equiv), **2** (52 mg, 0.15 mmol, 1.5 equiv), neoB–Bneo (90 mg, 0.40 mmol, 4.0 equiv), 1,4-dioxane (0.40 mL), and toluene (0.40 mL) according to **General Procedure A**. Purified by GPC ($CHCl_3$ then ethyl acetate): 9.4 mg (36%, 0.10 mmol scale); colorless oil; ^1H NMR (400 MHz, $CDCl_3$): δ 7.33- 7.27 (m, 4H), 7.24- 7.20 (m, 1H), 4.22 (d, *J* = 7.8 Hz, 1H), 3.51- 3.45 (m, 4H), 3.19 (s, 3H), 1.37- 1.32 (m, 1H), 0.99 (d, *J* = 7.2 Hz, 3H), 0.80 (s, 6H). $^{13}C\{^1H\}$ NMR (100 MHz, $CDCl_3$): δ 142.5, 128.0, 127.1, 127.0, 86.1, 71.9, 56.8, 31.5, 21.6, 11.4 (The carbon signal bound to boron was not observed due to quadrupolar relaxation.). ^{11}B NMR (128 MHz, $CDCl_3$): δ 30.11. HRMS (ESI) m/z $(M+Na)^+$ calcd for $C_{15}H_{23}BNaO_3$: 285.1633, found: 285.1630.

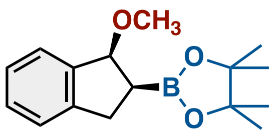

3r

2-((1*S,2*S**)-1-Methoxy-2,3-dihydro-1*H*-inden-2-yl)-4,4,5,5-tetramethyl-1,3,2-dioxaborolane (3r)**

The reaction was performed with $Cu(CH_3CN)_4PF_6$ (7.4 mg, 0.020 mmol, 10 mol%), CF_3-dppbz (20 mg, 0.020 mmol, 10 mol%), LiO*t*Bu (32 mg, 0.40 mmol, 2.0 equiv), **1r** (23 mg, 0.20 mmol, 1.0 equiv), **2**

(104 mg, 0.30 mmol, 1.5 equiv), pinB–Bpin (128 mg, 0.50 mmol, 2.5 equiv), 1,4-dioxane (0.80 mL), and toluene (0.80 mL) according to **General Procedure A**. It was purified silica gel column chromatography with hexane/ethyl acetate (40/1, v/v) then GPC ($CHCl_3$): 38 mg (70%, 0.20 mmol scale); colorless oil; ^{1}H NMR (400 MHz, $CDCl_3$): δ 7.37 (d, J = 7.4 Hz, 1H), 7.29-7.23 (m, 2H), 7.17 (t, J = 7.4 Hz, 1H), 4.84 (d, J = 6.6 Hz, 1H), 3.31 (s, 3H), 3.30 (dd, J = 6.9, 16.6 Hz, 1H), 2.87 (dd, J = 7.6, 16.4 Hz, 1H), 2.12-2.06 (m, 1H), 1.29 (s, 12H). ^{13}C{^{1}H} NMR (100 MHz, $CDCl_3$): δ 146.1, 142.4, 128.4, 125.6, 125.1, 125.0, 87.1, 87.0, 83.4, 56.1, 33.0, 25.1, 25.0, 24.7, 24.6 (The carbon signal bound to boron was not observed due to quadrupolar relaxation.). ^{11}B NMR (128 MHz, $CDCl_3$): δ 33.15. HRMS (ESI) m/z $(M+Na)^+$ calcd for $C_{16}H_{23}BNaO_3$: 297.1633, found: 297.1622.

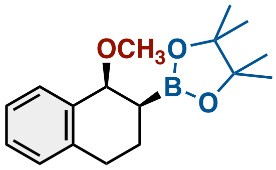

3s

2-((1*S,2S*)-1-Methoxy-1,2,3,4-tetrahydronaphthalen-2-yl)-4,4,5,5-tetramethyl-1,3,2-dioxaborolane (3s)**

The reaction was performed with $Cu(CH_3CN)_4PF_6$ (7.4 mg, 0.020 mmol, 10 mol%), CF_3-dppbz (20 mg, 0.020 mmol, 10 mol%), LiO*t*Bu (32 mg, 0.40 mmol, 2.0 equiv), **1s** (26 mg, 0.20 mmol, 1.0 equiv), **2** (104 mg, 0.30 mmol, 1.5 equiv), pinB–Bpin (128 mg, 0.50 mmol, 2.5 equiv), 1,4-dioxane (0.80 mL), and toluene (0.80 mL) according to **General Procedure A**. It was purified by silica gel column chromatography with hexane/ethyl acetate (40/1, v/v) then GPC ($CHCl_3$): 25 mg (44%, 0.20 mmol scale); colorless oil; ^{1}H NMR (400 MHz, $CDCl_3$): δ 7.23- 7.19 (m, 2H), 7.15-7.11 (m, 2H), 4.35 (d, J = 3.2 Hz, 1H), 3.34 (s, 3H), 2.92- 2.85 (m, 1H), 2.74- 2.65 (m, 1H), 2.15- 2.04 (m, 1H), 1.89- 1.82 (m, 1H), 1.39 (dt, J = 12.8, 3.5 Hz, 1H), 1.294 (s, 6H), 1.287 (s, 6H). ^{13}C{^{1}H} NMR (100 MHz, $CDCl_3$): δ 137.9, 135.9, 129.7 ,129.5 ,127.8, 124.8, 83.2, 78.5, 78.4, 56.7, 28.9, 25.1, 25.0, 24.54, 24.49, 18.8 (The carbon signal bound to boron was not observed due to quadrupolar relaxation.).^{11}B NMR (128 MHz, $CDCl_3$): δ 33.70. HRMS (ESI) m/z $(M+Na)^+$ calcd for $C_{17}H_{25}BNaO_3$: 311.1789, found: 311.1784.

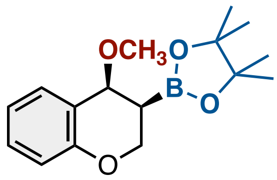

3t

2-((3*S,4*S**)-4-Methoxychroman-3-yl)-4,4,5,5-tetramethyl-1,3,2-dioxaborolane (3t)**

The reaction was performed with $Cu(CH_3CN)_4PF_6$ (3.7 mg, 0.010 mmol, 10 mol%), CF_3-dppbz (9.9 mg, 0.010 mmol, 10 mol%), LiO*t*Bu (16 mg, 0.20 mmol, 2.0 equiv), **1t** (13 mg, 0.10 mmol, 1.0 equiv), **2** (52 mg, 0.15 mmol, 1.5 equiv), pinB–Bpin (64 mg, 0.25 mmol, 2.5 equiv), 1,4-dioxane (0.40 mL), and toluene (0.40 mL) according to **General Procedure A**. It was purified by GPC ($CHCl_3$ then ethyl acetate): 20 mg (70%, 0.10 mmol scale);colorless oil; ^{1}H NMR (400 MHz, $CDCl_3$): δ 7.23- 7.19 (m, 1H), 7.16 (dd, *J* = 7.5, 1.7 Hz, 1H), 6.87- 6.82 (m, 2H), 4.35- 4.30 (m, 3H), 3.39 (s, 3H), 1.69 (ddd, *J* = 10.9, 6.8, 2.9 Hz, 1H), 1.29 (s, 6H), 1.29 (s, 6H). ^{13}C{^{1}H} NMR (100 MHz, $CDCl_3$): δ 154.7, 130.7, 129.7, 121.4, 118.9, 117.2, 83.6, 73.5, 63.3, 56.3, 25.0, 24.4. ^{11}B NMR (128 MHz, $CDCl_3$): *δ* 33.17. HRMS (ESI) m/z $(M+Na)^+$ calcd for $C_{16}H_{23}BNaO_4$: 313.1582, found: 313.1582.

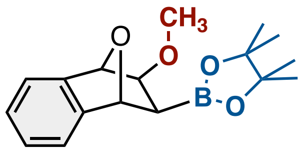

3u

2-((1*R,2*S**,3*S**,4*R**)-3-Methoxy-1,2,3,4-tetrahydro-1,4-epoxynaphthalen-2-yl)-4,4,5,5-tetramethyl-1,3,2-dioxaborolane (3u)**

The reaction was performed with $Cu(CH_3CN)_4PF_6$ (3.7 mg, 0.010 mmol, 10 mol%), CF_3-dppbz (9.9 mg, 0.010 mmol, 10 mol%), LiO*t*Bu (16 mg, 0.20 mmol, 2.0 equiv), **1u** (14 mg, 0.10 mmol, 1.0 equiv), **2** (52 mg, 0.15 mmol, 1.5 equiv), pinB–Bpin (64 mg, 0.25 mmol, 2.5 equiv), and toluene (0.80 mL) according to **General Procedure A**. It was purified by silica gel column chromatography with hexane/ethyl acetate (40/1, v/v) then GPC ($CHCl_3$): 9 mg (29%, 0.10 mmol scale); colorless oil; ^{1}H NMR (400 MHz, $CDCl_3$): *δ* 7.28-7.26 (m, 1H), 7.17-7.08 (m, 3H), 5.41 (s, 1H), 5.40 (s, 1H), 3.78 (d, *J* = 7.5 Hz, 1H), 3.45 (s, 3H), 1.59 (d, *J* = 7.5 Hz, 1H), 1.31 (s, 6H), 1.29 (s, 6H). ^{13}C{^{1}H} NMR (100 MHz, $CDCl_3$): *δ*148.9, 141.9, 127.1, 126.1, 120.5, 118.3, 85.2, 83.6, 81.2, 79.8, 57.9, 25.3, 24.3 (The carbon signal bound to boron was not observed due to quadrupolar relaxation.). ^{11}B NMR (128 MHz, $CDCl_3$): *δ* 32.36. HRMS (ESI) m/z $(M+Na)^+$ calcd for $C_{17}H_{23}BNaO_4$: 325.1582, found: 325.15811.

Copper-Catalyzed Methoxyboration of Styrenes with Diboron and Acetal-Based Peroxide Followed by Transesterification with H_2Bdan: General Procedure B

$Cu(CH_3CN)_4PF_6$ (10 mol%), CF_3-dppbz (10 mol%), and LiO*t*Bu (2.0 equiv) were placed in a 20 mL-Schlenk flask, which was filled with nitrogen by the standard Schlenk technique. 1,4-Dioxane and

toluene (1/1, v/v) were added, and the solution was stirred at room temperature for 15 min. pinB–Bpin (2.5 equiv) was then added, and after additional 5 min, the solution was cooled to 0 ˚C (cooling block). Finally, styrene **1** (1.0 equiv) and 2-(3,5-bis(trifluoromethyl)phenyl)-2-(methylperoxy)tetrahydro-2*H*-pyran (**2**, 1.5 equiv) were added, and the resulting solution was stirred for 18 h at 0 ˚C. The solution was filtered through a short pad of Na_2SO_4 and activated alumina, and the filtrate was evaporated under reduced pressure. The residue, 1,8-diaminonaphthalene (1.5 equiv), and imidazole (3.0 equiv) were placed in another 20 mL-Schlenk flask, which was filled with nitrogen by the standard Schlenk technique. A solution of $FeCl_3$ (25 mol%) in H_2O (0.20 mL) and MeCN (0.80 mL) were then added, and the mixture was stirred at room temperature overnight. The resulting mixture was diluted with water, extracted with ethyl acetate (20 mL) three times, and washed with brine. The combined organic layer was dried by filtration through a short pad of Na_2SO_4 and evaporated under reduced pressure. The residual material was purified by column chromatography on silica gel and GPC ($CHCl_3$) to give the corresponding methoxyborated product **3-Bdan**.

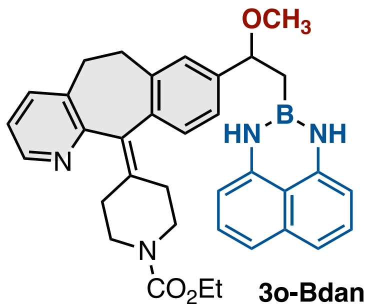

3o-Bdan

Ethyl 4-(8-(1-methoxy-2-(1*H*-naphtho[1,8-*de*][1,3,2]diazaborinin-2(3*H*)-yl)ethyl)-5,6-dihydro-11*H*-benzo[5,6]cyclohepta[1,2-*b*]pyridin-11-ylidene)piperidine-1-carboxylate (3o-Bdan)

The reaction was performed with $Cu(CH_3CN)_4PF_6$ (3.7 mg, 0.010 mmol, 10 mol%), CF_3-dppbz (9.9 mg, 0.010 mmol, 10 mol%), LiO*t*Bu (16 mg, 0.20 mmol, 2.0 equiv), **1o** (37 mg, 0.10 mmol, 1.0 equiv), **2** (52 mg, 0.15 mmol, 1.5 equiv), pinB–Bpin (64 mg, 0.25 mmol, 2.5 equiv), 1,4-dioxane (0.40 mL), toluene (0.40 mL), 1,8-diaminonaphthalene (24 mg, 0.15 mmol, 1.5 equiv), imidazole (20 mg, 0.30 mmol, 3.0 equiv), a solution of $FeCl_3$ (4.1 mg, 0.025 mmol, 25 mol%) in H_2O (0.20 mL), and MeCN (0.80 mL) according to **General Procedure B**. It was purified by silica gel column chromatography with hexane/ethyl acetate (2/1, v/v) then GPC (ethyl acetate): 13 mg (23%, 0.10 mmol scale); brown oil; ^{1}H NMR (400 MHz, CD_2Cl_2): δ 8.39 (d, J = 4.7 Hz, 1H), 7.55 (d, J = 7.6 Hz, 1H), 7.20- 7.14 (m, 4H), 7.05 (td, J = 8.3, 1.8 Hz, 2H), 6.95 (dd, J = 8.3, 0.7 Hz, 2H), 6.21- 6.18 (m, 2H), 5.84 (d, J = 12.8 Hz, 2H), 4.40- 4.34 (m, 1H), 4.1 (q, J = 7.1 Hz, 2H), 3.81-3.72 (m, 2H), 3.45-3.34 (m, 2H), 3.25- 3.17 (m, 2H),

3.23 (d, J = 4.0 Hz, 3H), 2.86- 2.79 (m, 2H), 2.50- 2.39 (m, 2H), 2.36- 2.20 (m, 2H), 1.40 (dd, J = 15.5, 8.1 Hz, 1H), 1.32 (dd, J = 10.2, 5.1 Hz, 1H), 1.24 (td, J = 7.2, 0.9 Hz, 3H). $^{13}C\{^1H\}$ NMR (100 MHz, CD_2Cl_2): δ 157.6, 157.1, 155.3, 143.2, 141.18, 141.15, 137.8, 137.77, 136.3, 129.49, 129.45, 127.5, 127.2, 127.1, 123.9, 122.4, 119.6, 117.1, 117.0, 105.3, 61.1, 56.34, 56.28, 44.8, 44.7, 31.8, 31.52, 31.50, 30.7, 30.6, 14.5 (Complicated ^1H and $^{13}C\{^1H\}$ signals were obtained because of conformers associated with two ring systems). ^{11}B NMR (128 MHz, CD_2Cl_2): δ 32.44. HRMS (APCI) m/z $(M+H)^+$ calcd for $C_{35}H_{38}BN_4O_3$: 573.3038, found: 573.3043.

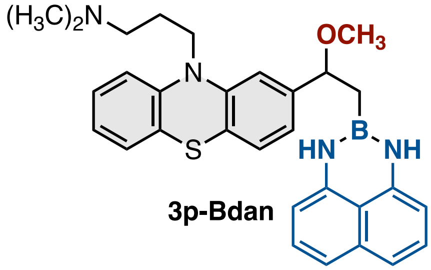

3p-Bdan

3-(2-(1-Methoxy-2-(1*H*-naphtho[1,8-*de*][1,3,2]diazaborinin-2(3*H*)-yl)ethyl)-10*H*-phenothiazin-10-yl)-*N*,*N*-dimethylpropan-1-amine (3p-Bdan)

The reaction was performed with $Cu(CH_3CN)_4PF_6$ (3.7 mg, 0.010 mmol, 10 mol%), CF_3-dppbz (9.9 mg, 0.010 mmol, 10 mol%), LiO*t*Bu (16 mg, 0.20 mmol, 2.0 equiv), **1p** (31 mg, 0.10 mmol, 1.0 equiv), **2** (52 mg, 0.15 mmol, 1.5 equiv), pinB–Bpin (64 mg, 0.25 mmol, 2.5 equiv), 1,4-dioxane (0.40 mL), toluene (0.40 mL), 1,8-diaminonaphthalene (24 mg, 0.15 mmol, 1.5 equiv), imidazole (20 mg, 0.30 mmol, 3.0 equiv), a solution of $FeCl_3$ (4.1 mg, 0.025 mmol, 25 mol%) in H_2O (0.20 mL), and MeCN (0.80 mL) according to **General Procedure B**. It was purified by column chromatography on NH_2 silica gel (Wakogel, $50NH_2$) with hexane/ethyl acetate (1/1, v/v) then GPC (ethyl acetate) (Caution: column chromatography on silica gel (60 N, spherical neutral, Kanto Chemical Co.) caused decomposition. Contact with $CHCl_3$ (a trace amount of HCl) should also be avoided.): 13 mg (25%, 0.10 mmol scale); brown oil; ^1H NMR (400 MHz, CD_2Cl_2): δ 7.19- 7.05 (m, 5H), 6.98- 6.89 (m, 6H), 6.28 (dd, J = 7.3, 1.0 Hz, 2H), 5.99 (brs, 2H), 4.39 (t, J = 6.6 Hz, 1H), 3.87 (t, J = 6.9 Hz, 2H), 3.25 (s, 3H), 2.27 (t, J = 7.2 Hz, 2H), 2.03 (s, 6H), 1.87- 1.82 (m, 2H), 1.44- 1.33 (m, 2H); $^{13}C\{^1H\}$ NMR (100 MHz, CD_2Cl_2): δ 146.3, 146.2, 144.3, 142.3, 142.0, 137.1, 128.3, 128.0, 127.9, 125.7, 124.8, 123.1, 121.3, 117.9, 116.4, 114.2, 106,4, 106.2, 82.6, 57.4, 57.1, 46.0, 45.9, 25.6 (The carbon signal bound to boron was not observed due to quadrupolar relaxation.).; ^{11}B NMR (128 MHz, CD_2Cl_2): δ 31.21; HRMS (APCI) m/z $(M+H)^+$ calcd for $C_{30}H_{34}BN_4OS$: 509.2546, found: 509.2542.

Copper-Catalyzed Methoxyboration of Styrene (*E*)-1a with B_2pin_2 and Acetal-Based Peroxide 2 (1.0 mmol scale; Scheme 2)

$Cu(CH_3CN)_4PF_6$ (37 mg, 0.10 mmol, 10 mol%), CF_3-dppbz (99 mg, 0.10 mmol, 10 mol%), and LiO*t*Bu (160 mg, 2.0 mmol, 2.0 equiv) were placed in a 20 mL-Schlenk flask, which was filled with nitrogen by the standard Schlenk technique. 1,4-Dioxane (4.0 mL) and toluene (4.0 mL) were added, and the solution was stirred at room temperature for 15 min. pinB–Bpin (640 mg, 2.5 mmol, 2.5 equiv) was then added, and after additional 5 min, the solution was cooled to 0 ˚C (cooling block). Finally, styrene (*E*)-**1a** (120 mg, 1.0 mmol, 1.0 equiv) and 2-(3,5-bis(trifluoromethyl)phenyl)-2-(methylperoxy)tetrahydro-2*H*-pyran (**2**, 520 mg, 1.5 mmol, 1.5 equiv) were added, and the resulting solution was stirred for 18 h at 0 ˚C. The solution was filtered through a short pad of Na_2SO_4 and activated alumina, and the filtrate was evaporated under reduced pressure. The residue was purified by column chromatography on silica gel with hexane/ethyl acetate (10/1, v/v) to give 2-((1*R**,2*S**)-1-methoxy-1-phenylpropan-2-yl)-4,4,5,5-tetramethyl-1,3,2-dioxaborolane (**3a**, 224 mg, 0.81 mmol, *syn/anti* >99:1) in 81% yield.

Oxidation

Oxidation of *syn*-**3a** (Scheme 2): A mixture of 2-((1*R**,2*S**)-1-methoxy-1-phenylpropan-2-yl)-4,4,5,5-tetramethyl-1,3,2-dioxaborolane (**3a**, 28 mg, 0.10 mmol, *syn/anti* >99:1), aq. NaOH (1 M, 1.0 mL, 1.0 mmol), and aq. H_2O_2 (30 w%, 0.50 mL) in THF (1.0 mL) was stirred for 1 h at room temperature. The resulting mixture was quenched with saturated aq. $Na_2S_2O_3$ and saturated aq. NH_4Cl, and extracted with ethyl acetate (20 mL) three times. The combined organic phase was dried by filtration through a short pad of Na_2SO_4 and evaporated in vacuo. The residual oil was purified by column chromatography on silica gel with hexane/ethyl acetate (4/1, v/v) to afford (1*R**,2*R**)-1-methoxy-1-phenylpropan-2-ol (**4**, 13 mg, 0.078 mmol, *syn/anti* >99:1) in 78% yield.

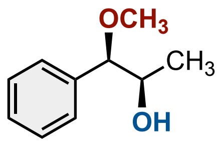

4 *syn/anti* >99:1

(1*R,2*R**)-1-Methoxy-1-phenylpropan-2-ol (4)**[S6]

It was purified by silica gel column chromatography with hexane/ethyl acetate (4/1, v/v): 13 mg (78%, 0.1 mmol scale); colorless oil; ^{1}H NMR ($CDCl_3$, 400 MHz): δ 7.39-7.26 (m, 5H), 3.85-3.79 (m, 2H), 3.24 (s, 3H), 3.07 (br, 1H), 0.96 (d, J = 6.0 Hz, 3H); ^{13}C{^{1}H} NMR ($CDCl_3$, 100 MHz): δ 138.4, 128.5, 128.3, 127.7, 89.5, 71.4, 56.7, 18.0; HRMS (ESI) m/z $(M+Na)^+$ calcd for $C_{10}H_{14}NaO_2$: 189.0886, found: 189.0890.

Methoxyboration of (*Z*)-**1a** followed by oxidation of *anti*-**3a** (Scheme S1): $Cu(CH_3CN)_4PF_6$ (7.4 mg, 0.020 mmol, 20 mol%), CF_3-dppbz (20 mg, 0.020 mmol, 20 mol%), and LiO*t*Bu (32 mg, 0.40 mmol, 4.0 equiv) were placed in a 20 mL-Schlenk flask, which was filled with nitrogen by the standard Schlenk technique. 1,4-Dioxane (0.40 mL) and toluene (0.40 mL) were added, and the solution was stirred at room temperature for 15 min. neoB–Bneo (90 mg, 0.40 mmol, 4.0 equiv) was then added, and after additional 5 min, the solution was cooled to 0 ˚C (cooling block). Finally, (*Z*)-**1a** (12 mg, 0.10 mml, 1.0 equiv) and 2-(3,5-bis(trifluoromethyl)phenyl)-2-(methylperoxy)tetrahydro-2*H*-pyran (**2**; 52 mg, 0.15 mmol, 1.5 equiv) were added, and the resulting solution was stirred for 18 h at 0 ˚C. The solution was filtered through a short pad of Na_2SO_4 and activated alumina, and the filtrate was evaporated under reduced pressure. The residue and $NaBO_3•H_2O$ (128 mg, 1.5 mmol, 15 equiv) were placed in a 50 mL flask, and THF (0.50 mL) and H_2O (0.50 mL) were added. The mixture was stirred at room temperature. After 7 h, the resulting mixture was diluted with water and extracted with ethyl acetate (20 mL) three times. The combined organic layer was dried by filtration through a short pad of Na_2SO_4 and evaporated in vacuo. The residual oil was purified by column chromatography on silica gel with hexane/ethyl acetate (4/1, v/v) and GPC ($CHCl_3$) to afford (1*S**,2*R**)-1-methoxy-1-phenylpropan-2-ol (**4**, 8.2 mg, 0.050 mmol, *syn/anti* <1:99) in 50% yield.

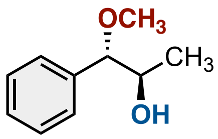

4 *syn/anti* <1:99

(1*S,2*R**)-1-Methoxy-1-phenylpropan-2-ol (4)**[S6]

Purified by silica gel column chromatography with hexane/ethyl acetate (4/1, v/v) then GPC ($CHCl_3$): 8.2 mg (50%, 0.10 mmol scale); colorless oil; ^{1}H NMR (400 MHz, $CDCl_3$): δ 7.40-7.29 (m, 5H), 4.11 (d, J = 4.7 Hz, 1H), 3.98-3.91 (m, 1H), 3.29 (s, 3H), 1.95-1.94 (br, 1H), 1.10 (d, J = 6.5 Hz, 3H); ^{13}C{^{1}H} NMR (100 MHz, $CDCl_3$): δ 138.0, 128.4, 128.0, 127.6, 87.5, 70.8, 57.2, 17.9; HRMS (ESI) m/z $(M+Na)^+$

calcd for $C_{10}H_{14}NaO_2$: 189.0886, found: 189.0882.

Methoxyboration of **1r** followed by oxidation of **3r** (Scheme S1): $Cu(CH_3CN)_4PF_6$ (7.4 mg, 0.020 mmol, 10 mol%), CF_3-dppbz (20 mg, 0.020 mmol, 10 mol%), and LiO*t*Bu (32 mg, 0.40 mmol, 2.0 equiv) were placed in a 20 mL-Schlenk flask, which was filled with nitrogen by the standard Schlenk technique. 1,4-Dioxane (0.80 mL) and toluene (0.80 mL) were added, and the solution was stirred at room temperature for 15 min. pinB–Bpin (127 mg, 0.50 mmol, 2.5 equiv) was then added, and after additional 5 min, the solution was cooled to 0 ˚C (cooling block). Finally, **1r** (23 mg, 0.20 mml, 1.0 equiv) and 2-(3,5-bis(trifluoromethyl)phenyl)-2-(methylperoxy)tetrahydro-2*H*-pyran (**2**; 104 mg, 0.30 mmol, 1.5 equiv) were added, and the resulting solution was stirred for 18 h at 0 ˚C. The solution was filtered through a short pad of Na_2SO_4 and activated alumina, and the filtrate was evaporated under reduced pressure. The residue and $NaBO_3•H_2O$ (245 mg, 3.0 mmol, 15 equiv) were placed in a 50 mL flask, and THF (1.0 mL) and H_2O (1.0 mL) were added. The mixture was stirred at room temperature. After 7 h, the resulting mixture was diluted with water and extracted with ethyl acetate (20 mL) three times. The combined organic layer was dried by filtration through a short pad of Na_2SO_4 and evaporated in vacuo. The residual oil was purified by column chromatography on silica gel with hexane/ethyl acetate (4/1, v/v) and GPC ($CHCl_3$) to afford (1*R**,2*S**)-1-methoxy-2,3-dihydro-1*H*-inden-2-ol (3r-O) (**3r-O**, 24 mg, 0.15 mmol, *cis/trans* >99:1) in 75% yield.

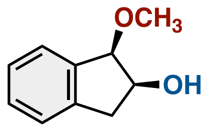

3r-O

(1*R,2*S**)-1-Methoxy-2,3-dihydro-1*H*-inden-2-ol (3r-O)**[S7]

Purified by silica gel column chromatography with hexane/ethyl acetate (4/1, v/v) then GPC ($CHCl_3$): 25 mg (75%, 0.20 mmol scale); colorless oil; ^{1}H NMR (400 MHz, $CDCl_3$): δ 7.38 (d, J = 8.0 Hz, 1H), 7.32-7.21 (m, 3H), 4.55-4.53 (m, 2H), 3.54 (s, 3H), 3.09 (dd, J = 15.8, 5.6 Hz, 1H), 2.98 (dd, J = 15.7, 4.5 Hz, 1H), 2.85-2.84 (br, 1H); ^{13}C{^{1}H} NMR (100 MHz, $CDCl_3$): δ 141.1, 139.8, 129.1, 126.6, 125.6, 125.5, 84.1, 72.4, 57.2, 39.1; HRMS (ESI) m/z $(M+Na)^+$ calcd for $C_{10}H_{12}NaO_2$: 187.0726, found: 187.0730.

Amination (Scheme 2)

In a glovebox filled with nitrogen, 2-((1*R**,2*S**)-1-methoxy-1-phenylpropan-2-yl)-4,4,5,5-tetramethyl-1,3,2-dioxaborolane (**3a**, 28 mg, 0.10 mmol, *syn/anti* >99:1), NH_2-DABCO (38 mg, 0.10 mmol),[S5] and KO*t*Bu (22 mg, 0.20 mmol) were placed in a 10 mL microwave reaction vessel. THF (1.2 mL) was then added, and the vessel was sealed with a cap and then taken out from the glovebox. The solution was heated at 100 ˚C (heat block). After 3 h, TFAA (28 µL, 0.20 mmol) was added and heated at 100 ˚C (heat block) for additional 1 h. After cooling to room temperature, the resulting mixture was quenched with water and extracted with ethyl acetate (20 mL) three times. The combined organic phase was dried by filtration through a short pad of Na_2SO_4 and evaporated in vacuo. The residual oil was purified by column chromatography on silica gel with hexane/ethyl acetate (4/1, v/v) to afford 2,2,2-trifluoro-*N*-((1*R**,2*R**)-1-methoxy-1-phenylpropan-2-yl)acetamide (**5**, 27 mg, 0.10 mmol, *syn/anti* >99:1) in >99% yield.

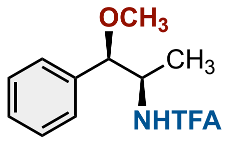

5

2,2,2-Trifluoro-*N*-((1*R,2*R**)-1-methoxy-1-phenylpropan-2-yl)acetamide (5)**

It was purified by silica gel column chromatography with hexane/ethyl acetate (4/1, v/v): 27 mg (quant., 0.1 mmol scale); colorless oil; ^{1}H NMR ($CDCl_3$, 400 MHz): δ 7.39-7.29 (m, 3H), 7.24-7.21 (m, 2H), 6.50, (br, 1H), 4.21-4.13 (m, 2H), 3.31 (s, 3H), 1.28 (d, J = 6.7 Hz, 3H); ^{13}C{^{1}H} NMR ($CDCl_3$, 100 MHz): δ 156.5 (q, J = 36.5 Hz), 137.8, 128.6, 128.4, 126.7, 115.8 (q, J = 286.5 Hz), 84.5, 57.4, 51.2, 17.4; ^{19}F{^{1}H} NMR ($CDCl_3$, 376 MHz): δ -62.39; HRMS (ESI) m/z $(M+Na)^+$ calcd for $C_{12}H_{14}F_3NNaO_2$: 284.0869, found: 284.0852.

Vinylation (Scheme 2)

To a solution of 2-((1*R**,2*S**)-1-methoxy-1-phenylpropan-2-yl)-4,4,5,5-tetramethyl-1,3,2-dioxaborolane (**3a**, 28 mg, 0.10 mmol, *syn/anti* >99:1) in THF (1.0 mL) was added vinylmagnesium bromide (1.0 M in THF, 0.60 mL, 0.40 mmol) dropwise at –78 ˚C. The resulting mixture was gradually allowed to warm to room temperature over 30 min and then cooled down to -78 °C again. A solution of iodine (102 mg,

0.40 mmol) in MeOH (0.40 mL) was added dropwise to the reaction mixture, followed 1 h later by a solution of NaOMe (43 mg, 0.80 mmol) in MeOH (0.80 mL). The reaction mixture was then allowed to warm to room temperature and stirred for additional 18 h. The reaction was quenched with saturated aq. $Na_2S_2O_3$. Extraction was repeated a total of three times with ethyl acetate (20 mL), and combined organic phase was dried by filtration through a short pad of Na_2SO_4 and then evaporated in vacuo. The residue was purified by silica gel column chromatography with hexane/ethyl acetate (20/1, v/v) to give ((1*S**,2*S**)-1-Methoxy-2-methylbut-3-en-1-yl)benzene (**6**; 13 mg, 0.075 mmol, *syn/anti* >99:1) in 75% yield.

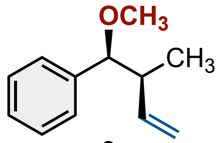

6

((1*S,2*S**)-1-Methoxy-2-methylbut-3-en-1-yl)benzene (6)**

It was purified by silica gel column chromatography with hexane/ethyl acetate (20/1, v/v): 13 mg (75%, 0.1 mmol scale); colorless oil; ^{1}H NMR ($CDCl_3$, 400 MHz): δ 7.34-7.25 (m, 5H), 5.93-5.84 (m, 1H), 5.054-5.051 (m, 1H), 5.02 (ddd, *J* = 6.4, 1.8, 1.0 Hz, 1H), 3.90 (d, *J* = 7.4 Hz, 1H), 3.19 (s, 3H), 2.56-2.47 (m, 1H), 0.84 (d, *J* = 6.9 Hz, 3H); ^{13}C{^{1}H} NMR ($CDCl_3$, 100 MHz): δ 141.1, 140.4, 128.1, 127.55, 127.52, 114.3, 88.2, 57.0, 44.3, 16.4; HRMS (ESI) m/z $(M+Na)^+$ calcd for $C_{12}H_{16}NaO$: 199.1093, found: 199.1094.

Homologation (Scheme 2)

To a solution of 2-((1*R**,2*S**)-1-methoxy-1-phenylpropan-2-yl)-4,4,5,5-tetramethyl-1,3,2-dioxaborolane (**3a**, 28 mg, 0.10 mmol, *syn/anti* >99:1) and bromochloromethane (26 mg, 0.20 mmol) in THF (1.0 mL) at –78 °C was added *n*-BuLi (1.51 M hexane solution, 0.11 mL, 0.17 mmol), and the solution was stirred at the same temperature for 30 min. The mixture was allowed to warm to room temperature over 30 min and then heated at 60 °C (oil bath) for additional 3 h. The resulting mixture was quenched with saturated aq. NH_4Cl and extracted with ethyl acetate (20 mL) three times. The combined organic layer was dried by filtration through a short pad of Na_2SO_4, and the solvent was removed under reduced pressure. The residual oil was purified by silica gel column chromatography on silica gel with hexane/ethyl acetate (40/1, v/v) and GPC ($CHCl_3$) to give 2-((2*S**,3*S**)-3-methoxy-2-methyl-3-phenylpropyl)-4,4,5,5-tetramethyl-1,3,2-dioxaborolane (**7**; 21 mg, 0.071 mmol, syn/anti >99:1) in 71%

yield.

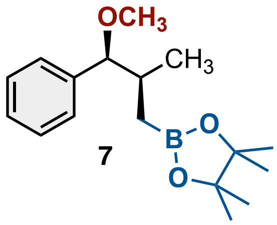

7

2-((2*S,3*S**)-3-Methoxy-2-methyl-3-phenylpropyl)-4,4,5,5-tetramethyl-1,3,2-dioxaborolane (7)**

It was purified by silica gel column chromatography with hexane/ethyl acetate (20/1, v/v): 21 mg (71%, 0.1 mmol scale); colorless oil; ^{1}H NMR ($CDCl_3$, 400 MHz): δ 7.34-7.23 (m, 5H), 3.65 (d, *J* = 8.3 Hz, 1H), 3.14 (s, 3H), 2.13-2.02 (m, 1H), 1.26 (s, 12H), 1.01 (dd, *J* = 15.3, 7.1 Hz, 1H), 0.72 (dd, *J* = 15.2, 7.1 Hz, 1H), 0.71 (d, *J* = 6.8 Hz, 3H); ^{13}C{^{1}H} NMR ($CDCl_3$, 100 MHz): δ 141.2, 128.0, 127.7, 127.4, 90.4, 82.7, 56.6, 36.7, 24.97, 24.85, 19.3 (The carbon signal bound to boron was not observed because of quadrupolar relaxation.); ^{11}B NMR ($CDCl_3$, 128 MHz): δ 33.90; HRMS (ESI) m/z $(M+Na)^+$ calcd for $C_{17}H_{27}BNaO_3$: 313.1946, found: 313.1939.

Stereochemical Assignment

The relative stereochemistry of *syn*- and *anti*-**3a**[S6] as well as **3r**[S7] was determined by comparison with the reported ^{1}H NMR spectra after the oxidation.

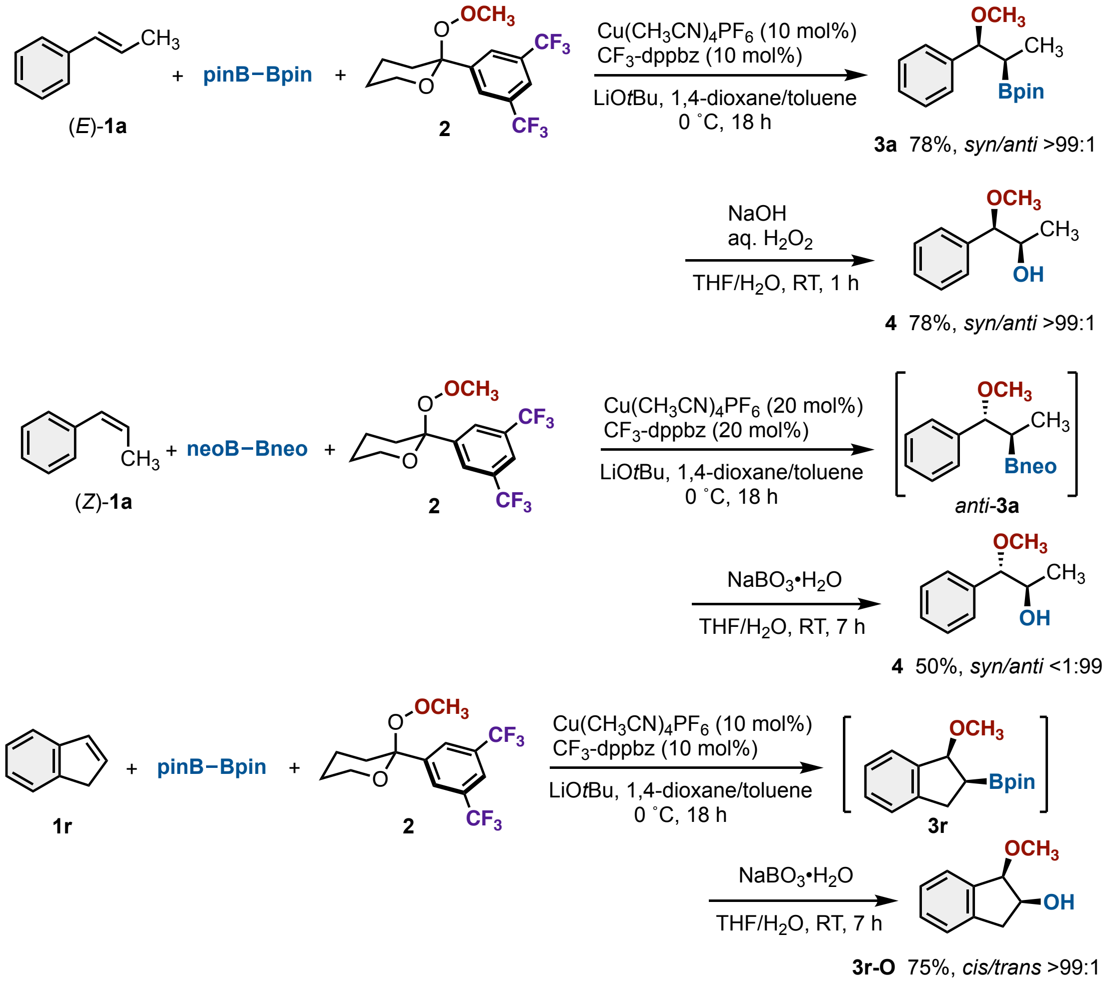

Scheme S1. Oxidation and stereochemical assignment.

Detailed Optimization Studies

Table S1. Optimization studies for copper-catalyzed methoxyboration of (*E*)-*β*-methylstyrene [(*E*)-**1a**] with pinB–Bpin and acetal-based peroxide **2**: catalyst screening.[a]

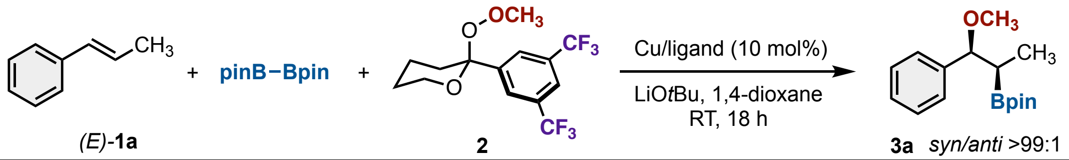

entry	Cu	ligand	yield of **3a** (%)[b]
1	$Cu(OAc)_2$	dppbz	11
2	$Cu(OAc)_2$	MeO-dppbz	8
3	$Cu(OAc)_2$	*p*-CF_3-dppbz	8
4	***$Cu(OAc)_2$***	***CF_3-dppbz***	***28***
5	$Cu(OAc)_2$	F_3-dppbz	26
6	$Cu(OAc)_2$	*t*Bu-dppbz	15
7	$Cu(OAc)_2$	TMS-dppbz	4
8	$Cu(OAc)_2$	DTBM-dppbz	9
9	$Cu(OAc)_2$	CF_3-dppe	6
10	$Cu(OAc)_2$	CF_3-dppf	10
11[c]	$Cu(OAc)_2$	$P[3,5-(CF_3)_2C_6H_3]_3$	2
12	CuOAc	CF_3-dppbz	17
13	CuCl	CF_3-dppbz	7
14	***$Cu(CH_3CN)_4PF_6$***	***CF_3-dppbz***	***61***
15	$Cu(CH_3CN)_4BF_4$	CF_3-dppbz	3
16	$Cu(CH_3CN)_4OTf$	CF_3-dppbz	8
17	CuOTf•1/2toluene	CF_3-dppbz	39

[a] Reaction conditions: **1a** (0.10 mmol), pinB–Bpin (0.25 mmol), **2** (0.15 mmol), Cu (0.010 mmol), ligand (0.010 mmol), LiO*t*Bu (0.20 mmol), 1,4-dioxane (0.80 mL), RT, 18 h, N_2. [b] Estimated by ^1H NMR with 1-methylnaphthalene as the internal standard. [c] With $P[3,5-(CF_3)_2C_6H_3]_3$ (0.020 mmol; 20 mol%).

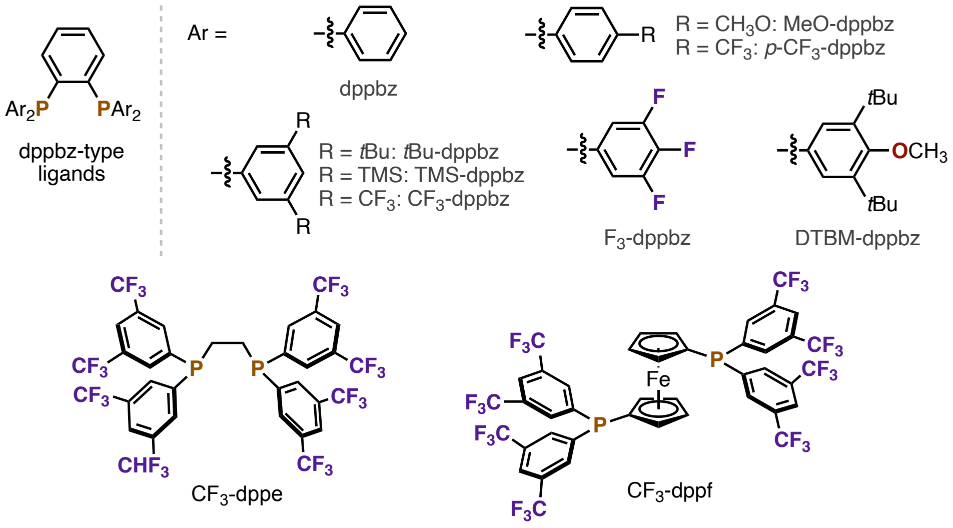

Table S2. Optimization studies for copper-catalyzed methoxyboration of (*E*)-*β*-methylstyrene [(*E*)-**1a**] with pinB–Bpin and acetal-based peroxide **2**: solvent and temperature screening.[a]

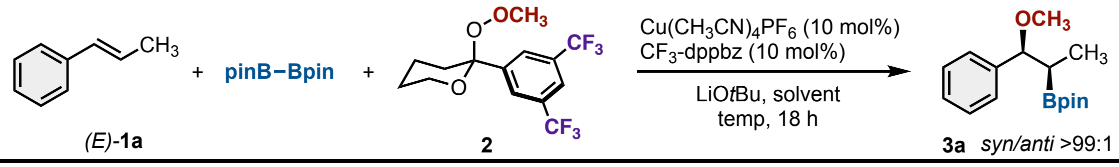

entry	solvent	temp (˚C)	yield of **3a** (%)[b]
1	1,4-dioxane	RT	61
2	THF	RT	32
3	CPME	RT	50
4	toluene	RT	57
5	toluene	0	71
6	toluene	–20	67
7[c]	***1,-dioxane/toluene (1:1)***	***0***	***82 (78)***
8[c,d]	1,-dioxane/toluene (1:1)	0	0

[a] Reaction conditions: **1a** (0.10 mmol), pinB–Bpin (0.25 mmol), **2** (0.15 mmol), $Cu(CH_3CN)_4PF_6$ (0.010 mmol), CF_3-dppbz (0.010 mmol), LiO*t*Bu (0.20 mmol), solvent (0.80 mL), temp, 18 h, N_2. [b] Estimated by ^1H NMR with 1-methylnaphthalene as the internal standard. Isolated yield is in parentheses. [c] In 1,4-dioxane (0.4 mL) and toluene (0.4 mL). [d] Without LiO*t*Bu.

Table S3. Optimization studies for copper-catalyzed methoxyboration of (*E*)-*β*-methylstyrene [(*E*)-**1a**] with pinB–Bpin and acetal-based peroxide **2**: peroxide screening.[a]

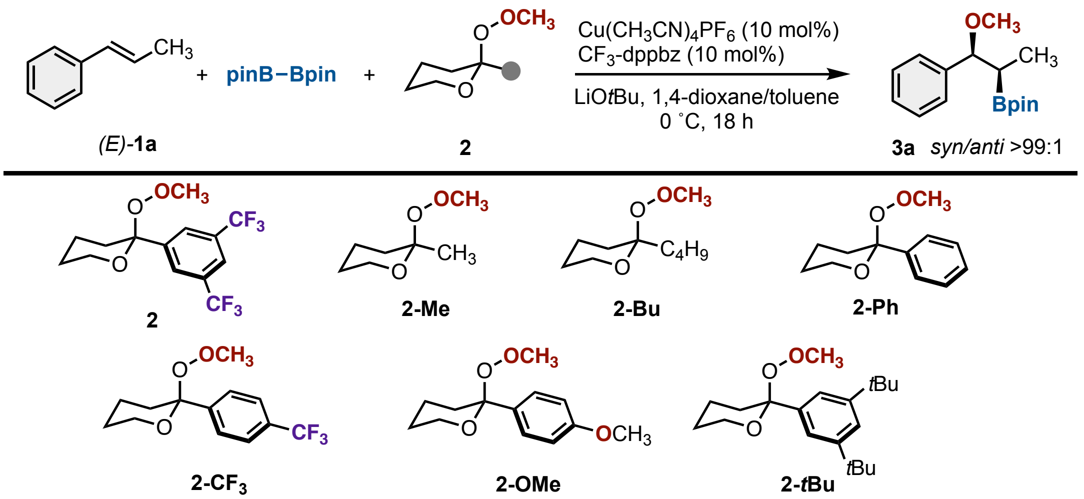

entry	peroxide **2**	yield of **3a** (%)[b]	entry	peroxide **2**	yield of **3a** (%)[b]
1	***2***	***82 (78)***	5	**2-CF₃**	63
2	**2-Me**	49	6	**2-OMe**	17
3	**2-Bu**	53	7	**2-*t*Bu**	28
4	**2-Ph**	49			

[a] Reaction conditions: **1a** (0.10 mmol), pinB–Bpin (0.25 mmol), **2** (0.15 mmol), $Cu(CH_3CN)_4PF_6$ (0.010 mmol), CF_3-dppbz (0.010 mmol), LiO*t*Bu (0.20 mmol), 1,4-dioxane/toluene (0.40/0.40 mL), 0 ˚C, 18 h, N_2. [b] Estimated by ^{1}H NMR with 1-methylnaphthalene as the internal standard. Isolated yield is in parentheses.

Table S4. Optimization studies for copper-catalyzed methoxyboration of (*E*)-*β*-methylstyrene [(*E*)-**1a**] with diboron and acetal-based peroxide **2**: diboron screening.[a]

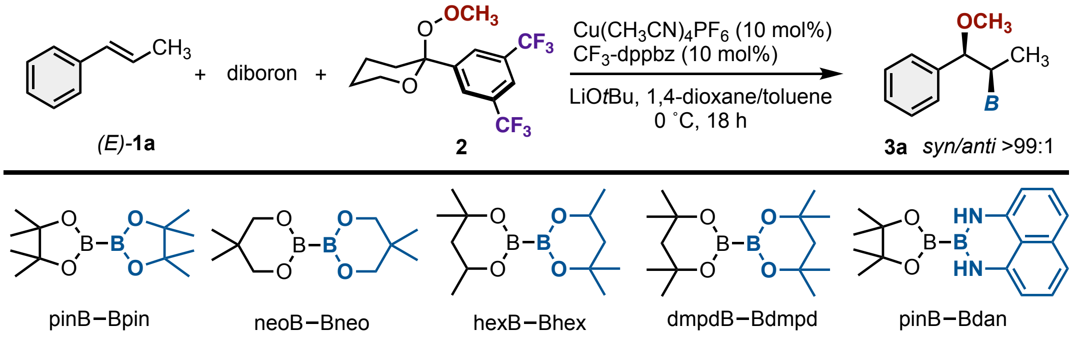

entry	diboron	**3**, yield (%)[b]	entry	diboron	**3**, yield (%)[b]
1	***pinB–Bpin***	***3a, 82 (78)***	4	dmpdB–Bdmpd	**3a-Bdmpd**, 2
2	neoB–Bneo	**3a-Bneo**, 22	5	pinB–Bdan	**3a-Bdan**, 12

3	hexB–Bhex	**3a-Bhex**, 39			

[a] Reaction conditions: **1a** (0.10 mmol), diboron (0.25 mmol), **2** (0.15 mmol), $Cu(CH_3CN)_4PF_6$ (0.010 mmol), CF_3-dppbz (0.010 mmol), LiO*t*Bu (0.20 mmol), 1,4-dioxane/toluene (0.40/0.40 mL), 0 ˚C, 18 h, N_2. [b] Estimated by ^{1}H NMR with 1-methylnaphthalene as the internal standard. Isolated yield is in parentheses.

Table S5. Optimization studies for copper-catalyzed methoxyboration of (*Z*)-*β*-methylstyrene [(*Z*)-**1a**] with diboron and acetal-based peroxide **2**[a]

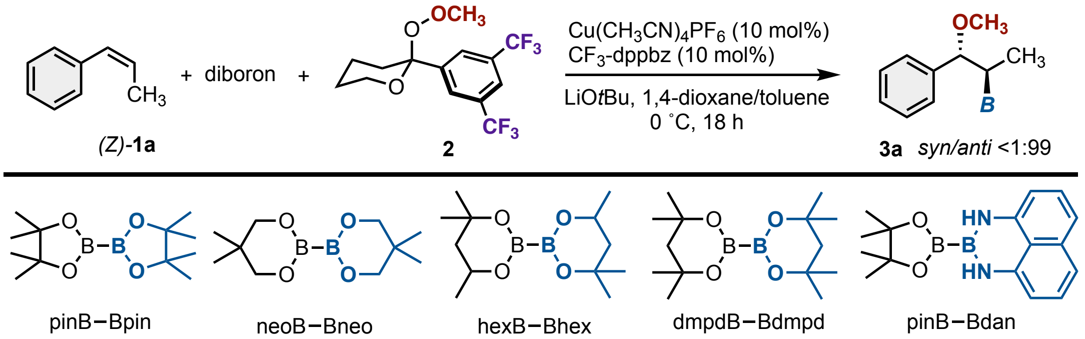

entry	diboron	**3**, yield (%)[b]	entry	diboron	**3**, yield (%)[b]
1	pinB–Bpin	**3a**, 11	4	dmpdB–Bdmpd	**3a-Bdmpd**, 3
2	***neoB–Bneo***	***3a-Bneo, 43***	5	pinB–Bdan	**3a-Bdan**, 8
3	hexB–Bhex	**3a-Bhex**, 39	***6[c]***	***neoB–Bneo***	***3a-Bneo, 59 (36)***

[a] Reaction conditions: **1a** (0.10 mmol), diboron (0.25 mmol), **2** (0.15 mmol), $Cu(CH_3CN)_4PF_6$ (0.010 mmol), CF_3-dppbz (0.010 mmol), LiO*t*Bu (0.20 mmol), 1,4-dioxane/toluene (0.40/0.40 mL), 0 ˚C, 18 h, N_2. [b] Estimated by ^{1}H NMR with 1-methylnaphthalene as the internal standard. Isolated yield is in parentheses. [c] With neoB–Bneo (0.40 mmol), $Cu(CH_3CN)_4PF_6$ (0.020 mmol), CF_3-dppbz (0.020 mmol), and LiO*t*Bu (0.40 mmol).

Recovery and Recycle of Hemiacetal Fragment

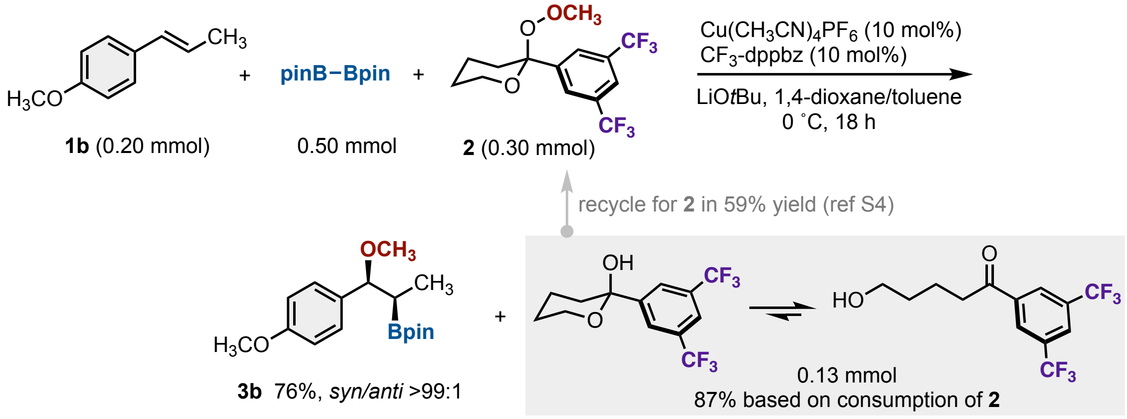

Scheme S2. Recovery of hemiacetal fragment and its ring-opening form.

Effect of Radical Trapping Reagent

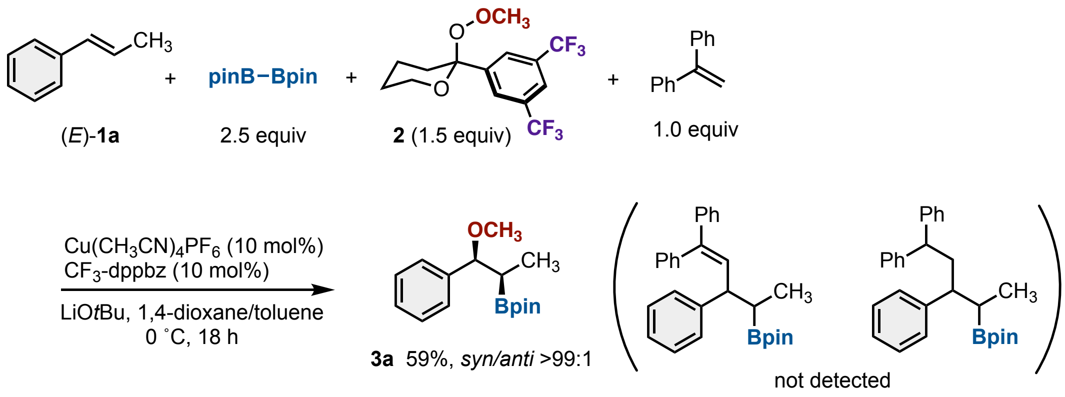

Scheme S3. Reaction of (*E*)-**1a** in the presence of 1,1-diphenylethene.

Unsuccessful Substrates

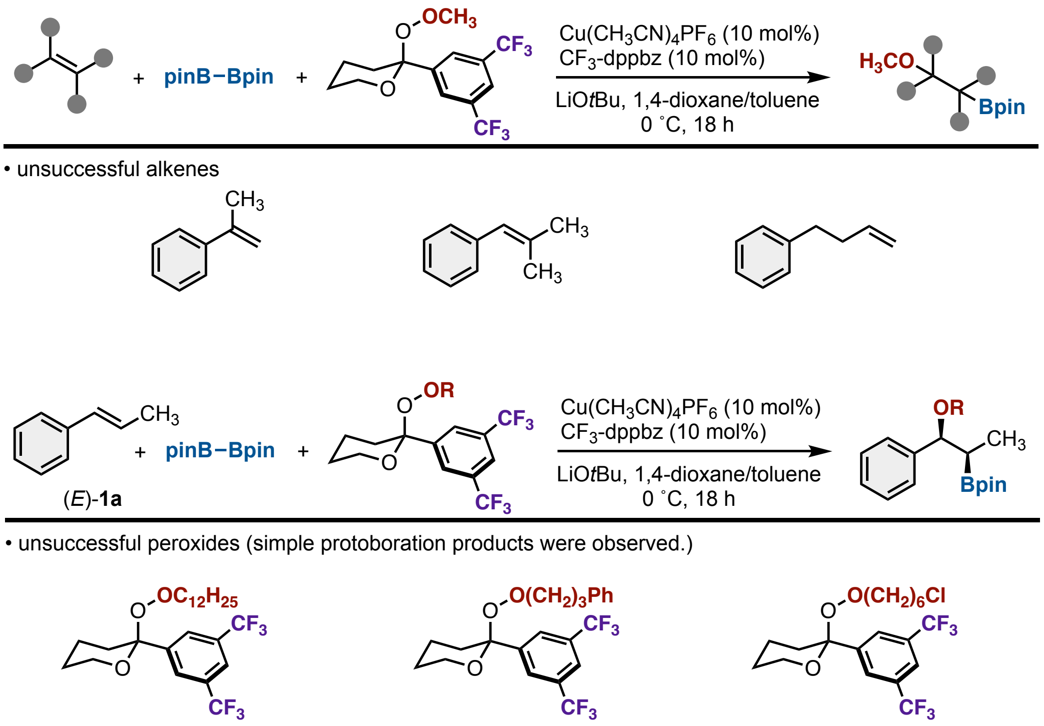

Scheme S4. Unsuccessful alkenes and peroxides.

Attempts of Asymmetric Catalysis

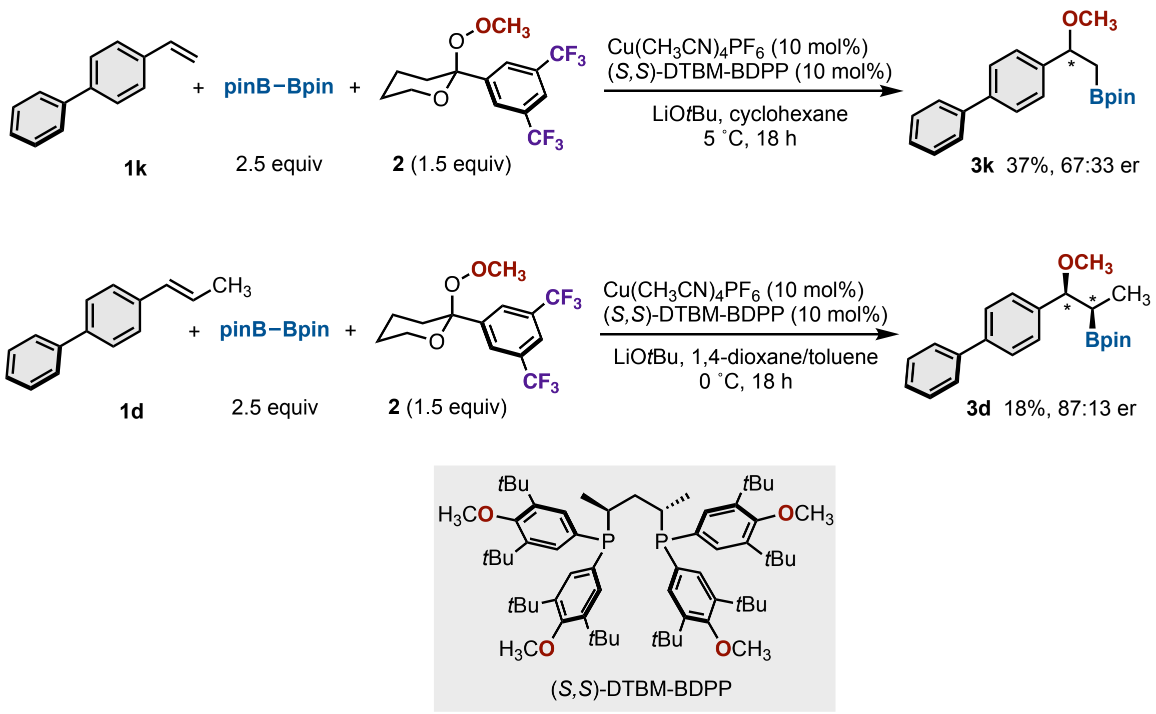

Scheme S5. Preliminary attempts of enantioselective methoxyboration with (*S,S*)-DTBM-BDPP.

Chiral HPLC Charts of Enantioenriched Product

3k: The enantiomeric ratio was determined by HPLC analysis in comparison with authentic racemic material (CHIRALPAK AD-H column, 99.8/0.2 hexane/isopropyl alcohol, 0.5 mL/min, major isomer: t_R = 24.3 min, minor isomer: t_R = 35.8 min, UV detection at 250 nm, 30 ºC).

rac-**3k**

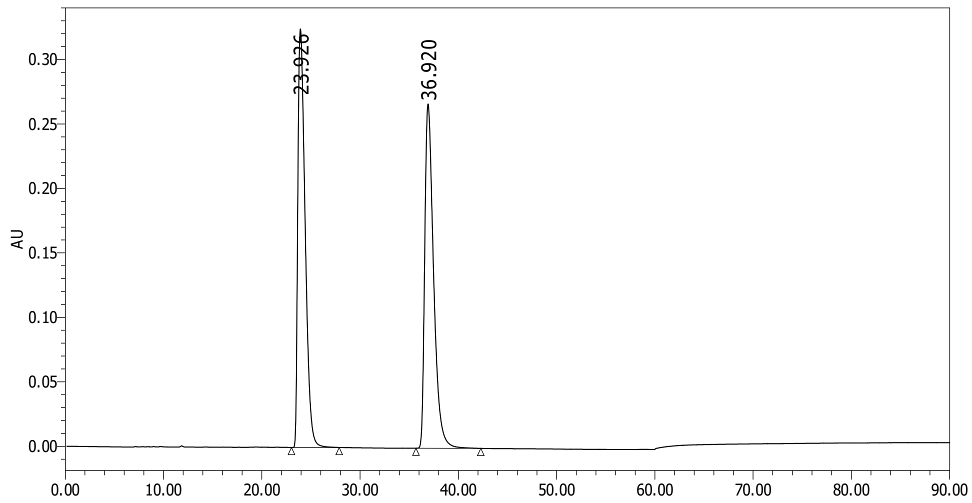

Peak #	Ret. Time	Area	Area %
1	23.926	16862197	50.13
2	36.920	16773983	49.87

enantioenriched-**3k** (from Scheme S5)

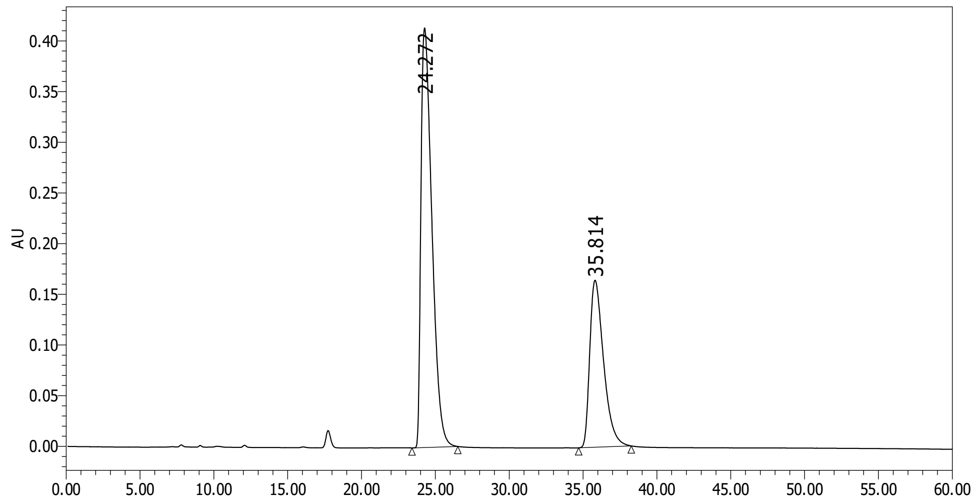

Peak #	Ret. Time	Area	Area %
1	24.272	21315403	66.57
2	35.814	10704393	33.43

3d: The enantiomeric ratio was determined by HPLC analysis in comparison with authentic racemic material (CHIRALPAK AD-H column, 99.5/0.5 hexane/isopropyl alcohol, 0.5 mL/min, major isomer: t_R = 11.0 min, minor isomer: t_R = 10.1 min, UV detection at 224.9 nm, 30 ºC).

rac-**3d**

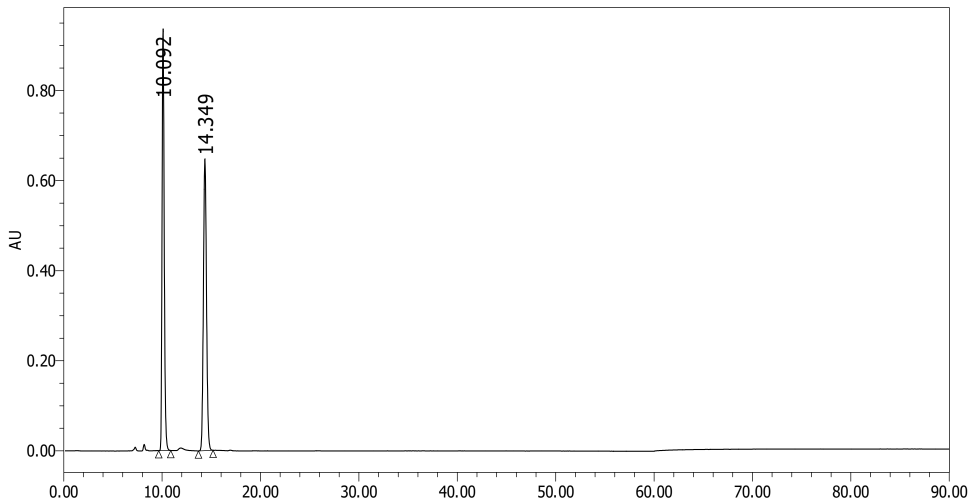

Peak #	Ret. Time	Area	Area %
1	10.092	14389700	50.34
2	14.349	14194371	49.66

enantioenriched-**3d** (from Scheme S5)

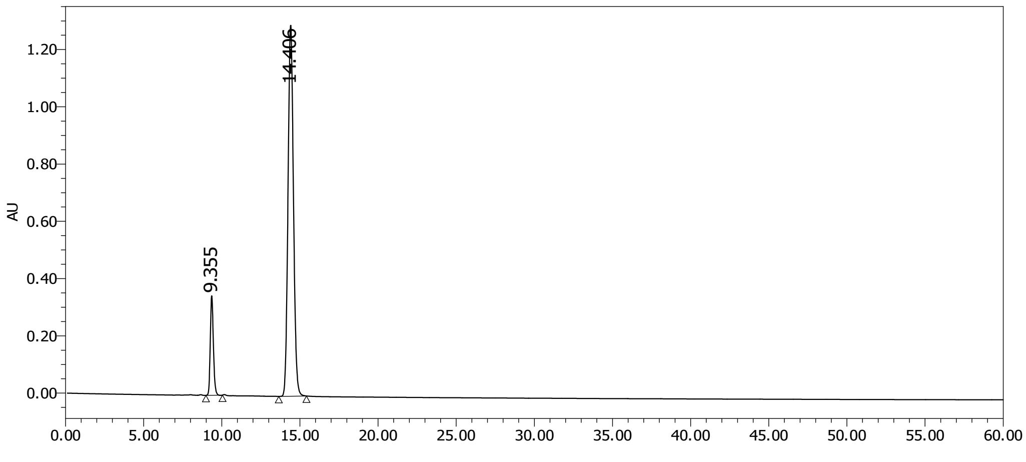

Peak #	Ret. Time	Area	Area %
1	9.355	4758953	347913
2	14.406	31233303	1296550

Copies of NMR Spectra

[^{1}H, ^{13}C{^{1}H}, and ^{11}B NMR Spectra of *syn*-**3a**]

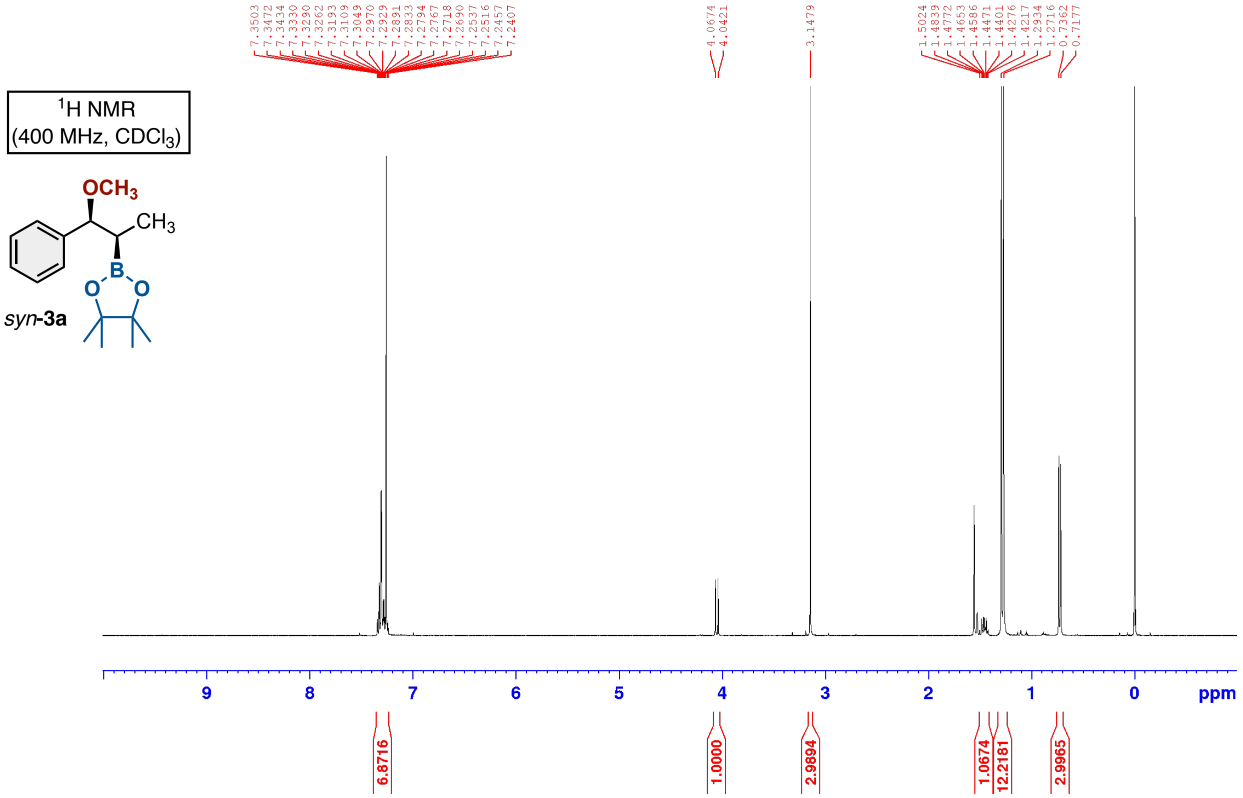

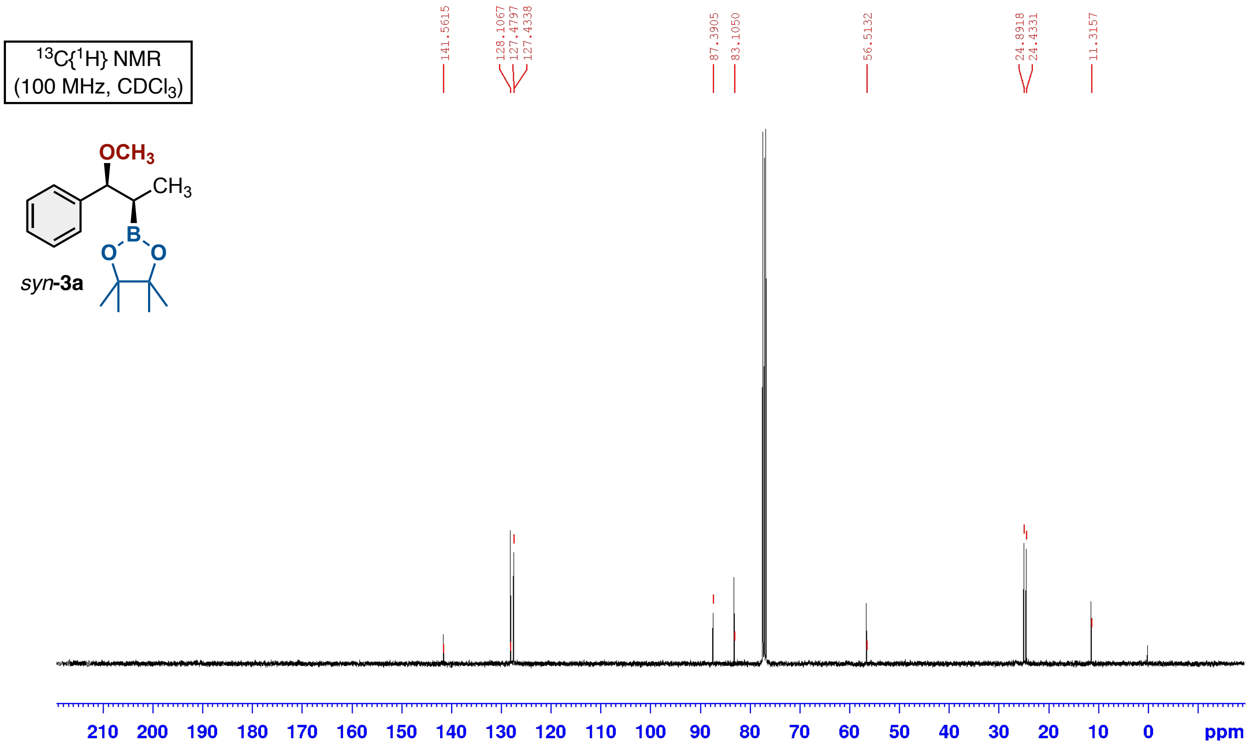

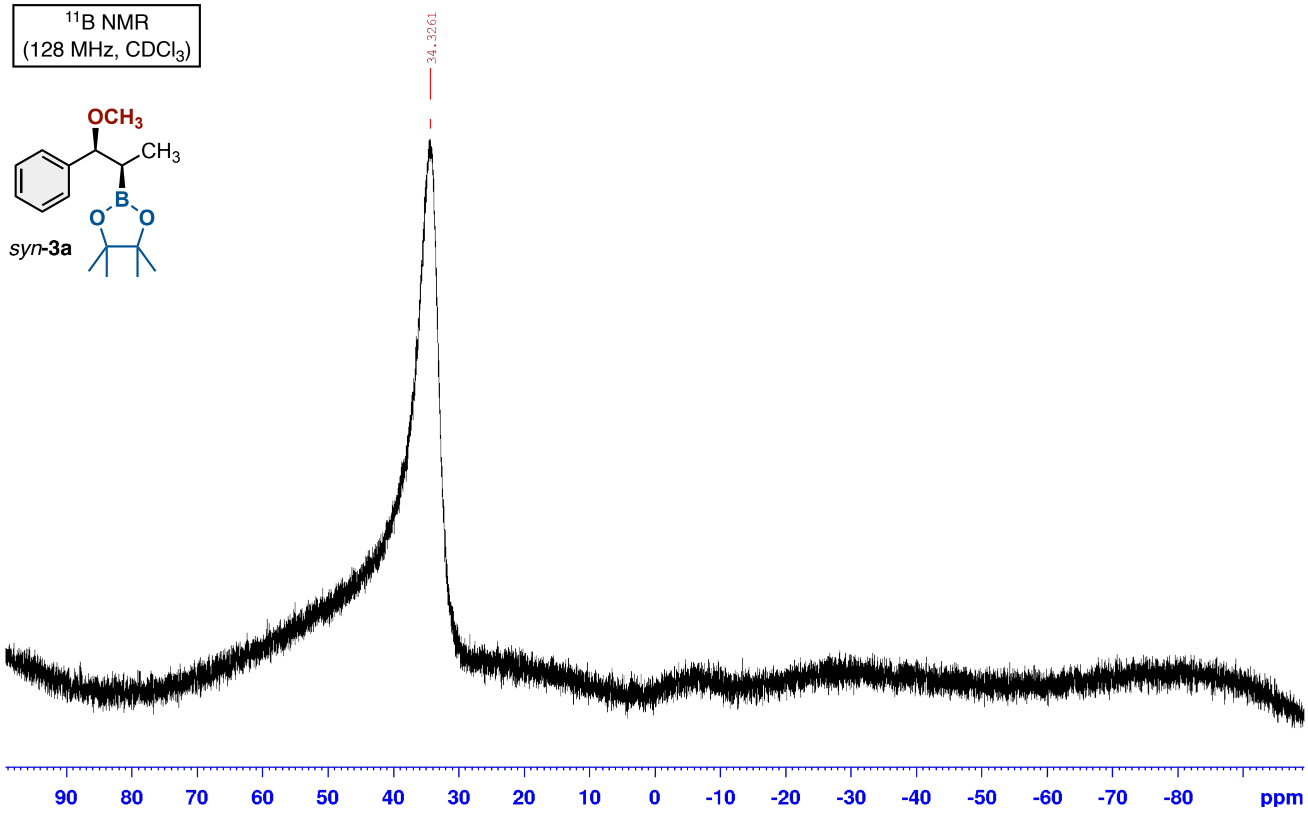

11B NMR
(128 MHz, CDCl3)
OCH3
CH3
B
O
O
syn-3a
34.3261
90
80
70
60
50
40
30
20
10
0
-10
-20
-30
-40
-50
-60
-70
-80
ppm

[^{1}H, ^{13}C{^{1}H}, and ^{11}B NMR Spectra of **3b**]

^{1}H NMR
(400 MHz, $CDCl_3$)

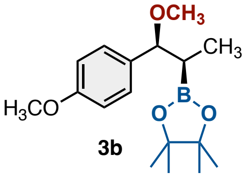

3b

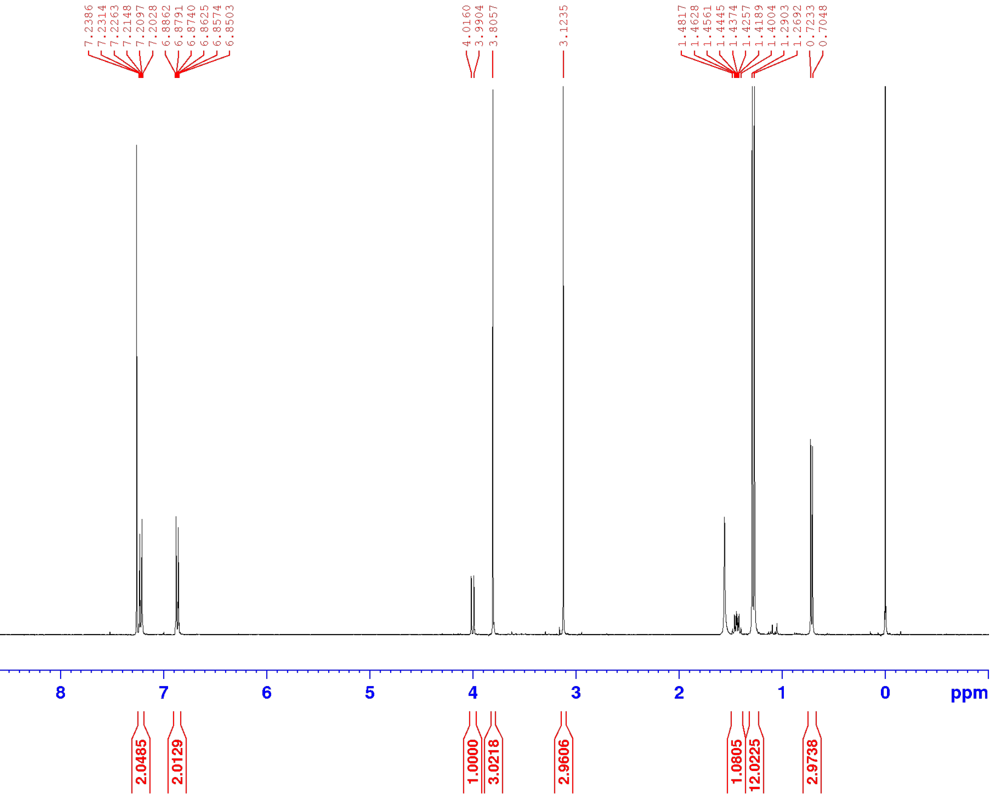

^{13}C{^{1}H} NMR
(100 MHz, $CDCl_3$)

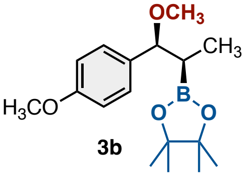

3b

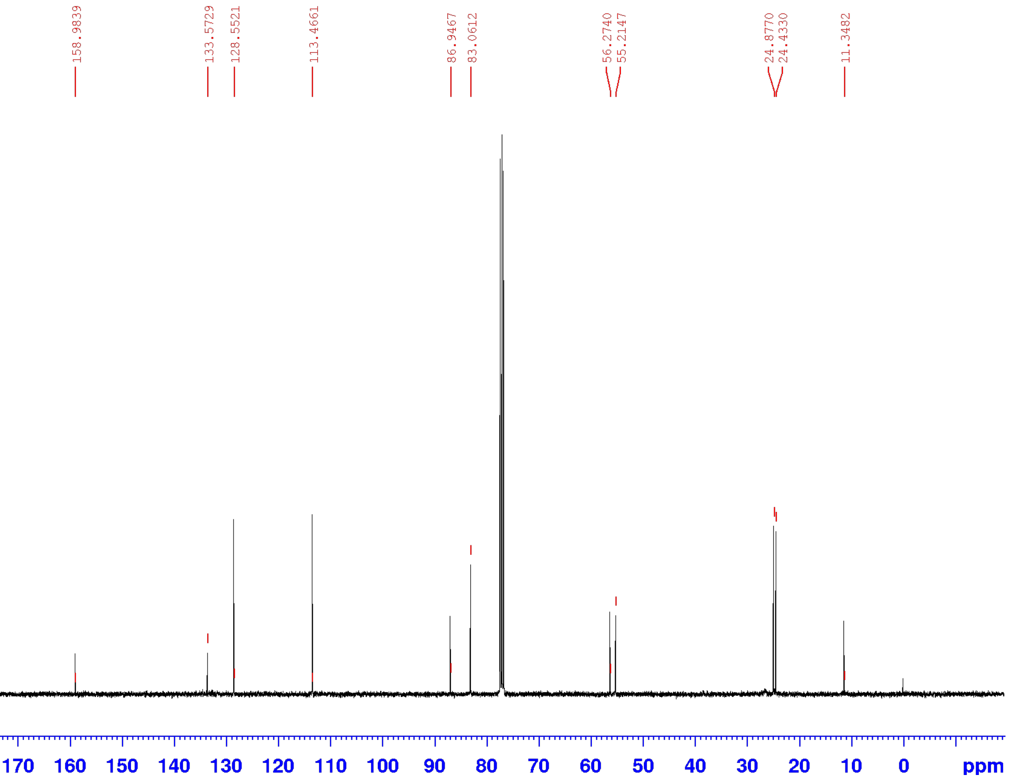

^{11}B NMR
(128 MHz, $CDCl_3$)

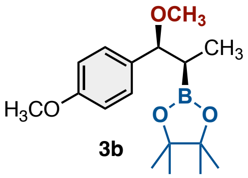

3b

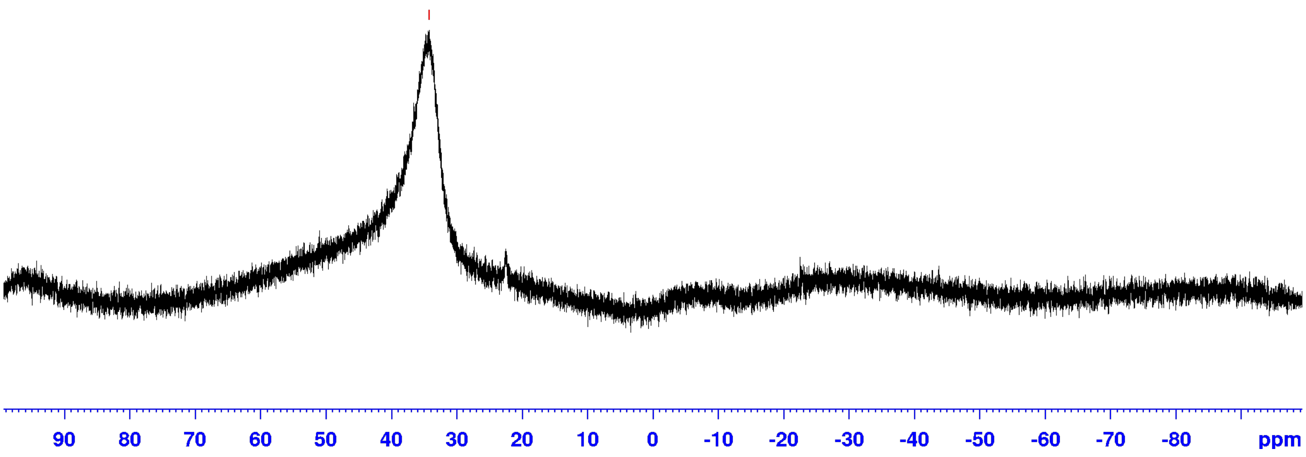

[^{1}H, ^{13}C{^{1}H}, ^{11}B, and ^{19}F{^{1}H} NMR Spectra of **3c**]

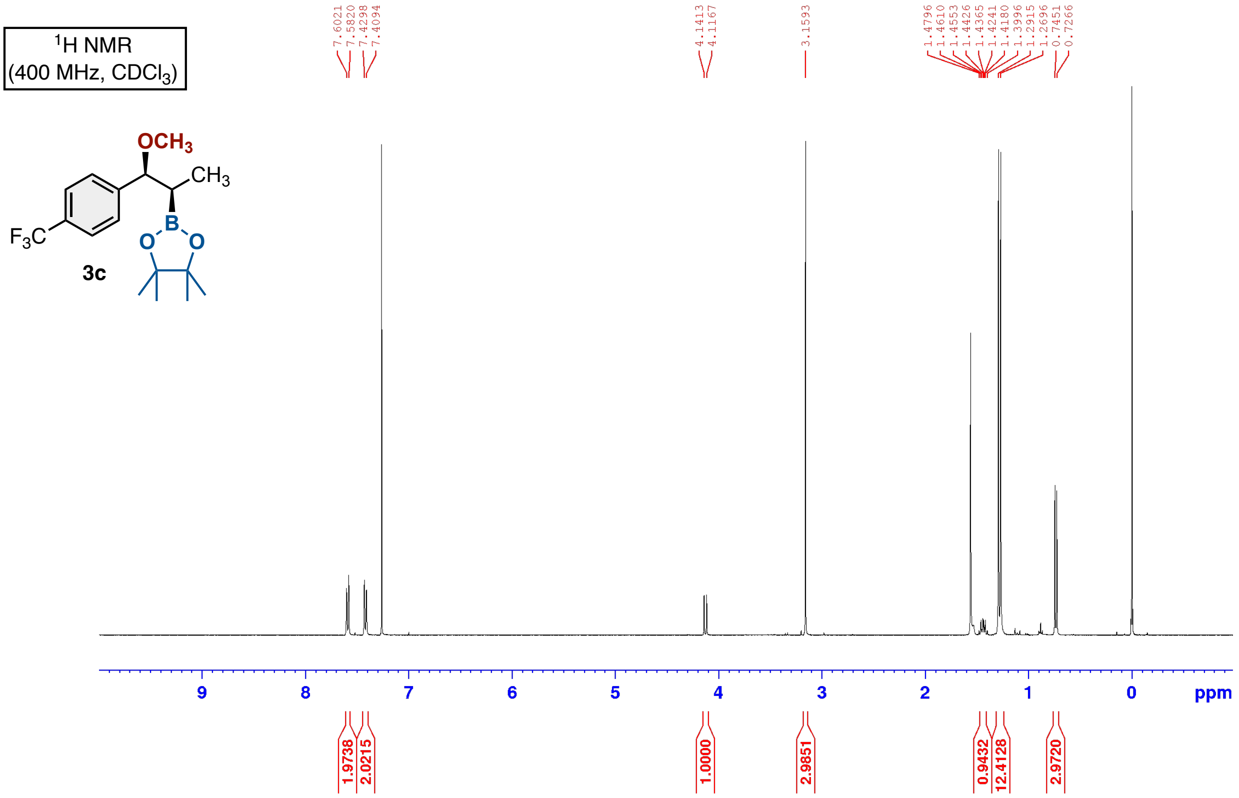

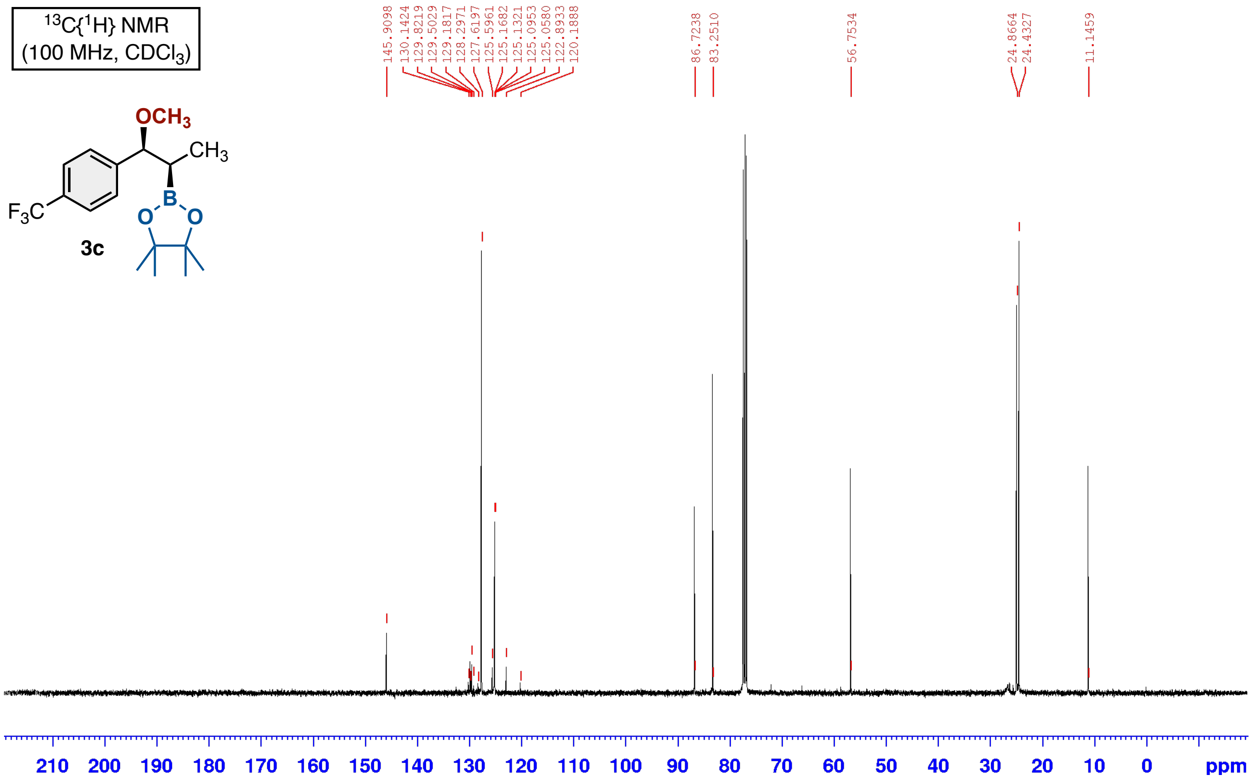

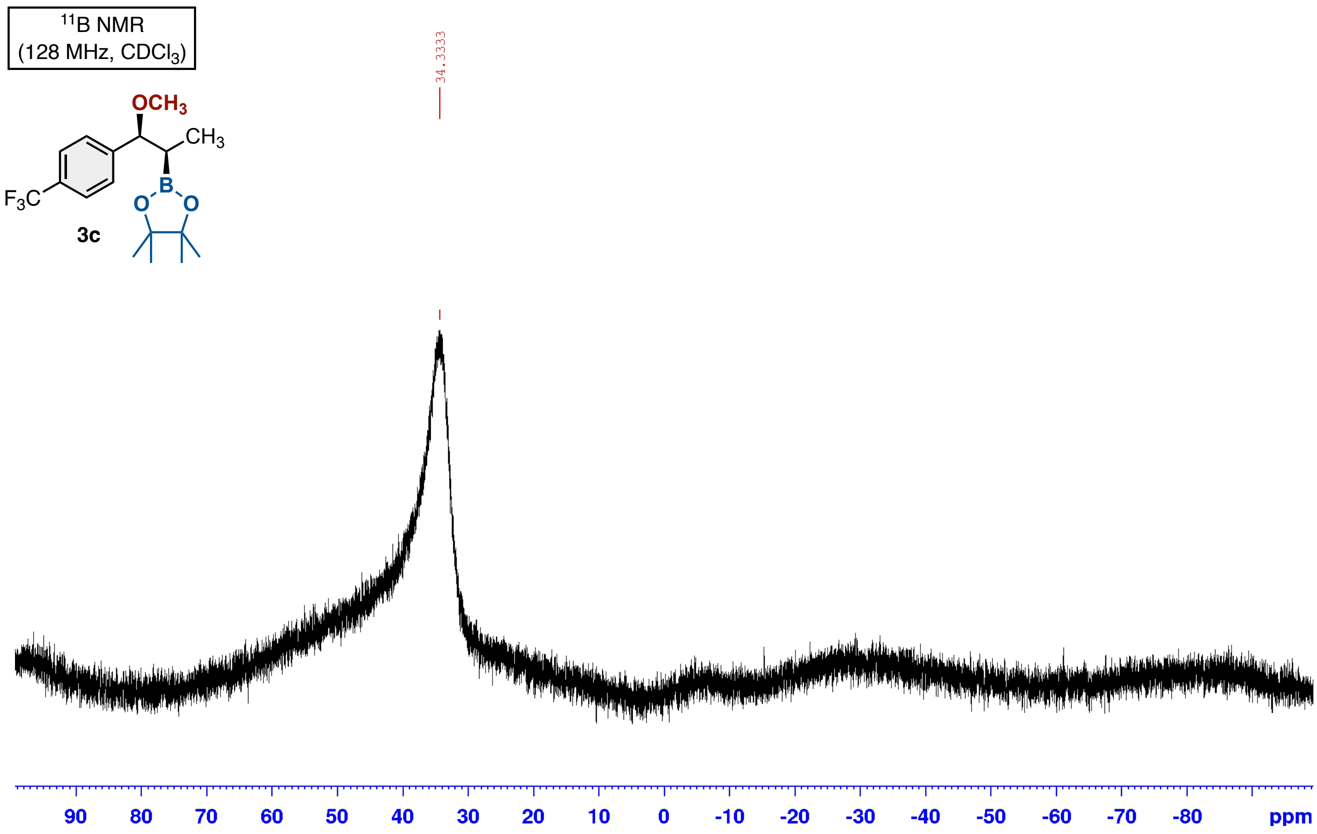

^{11}B NMR
(128 MHz, CDCl$_3$)
OCH$_3$
CH$_3$
F$_3$C
B
O
O
3c
34.3333
90
80
70
60
50
40
30
20
10
0
-10
-20
-30
-40
-50
-60
-70
-80
ppm

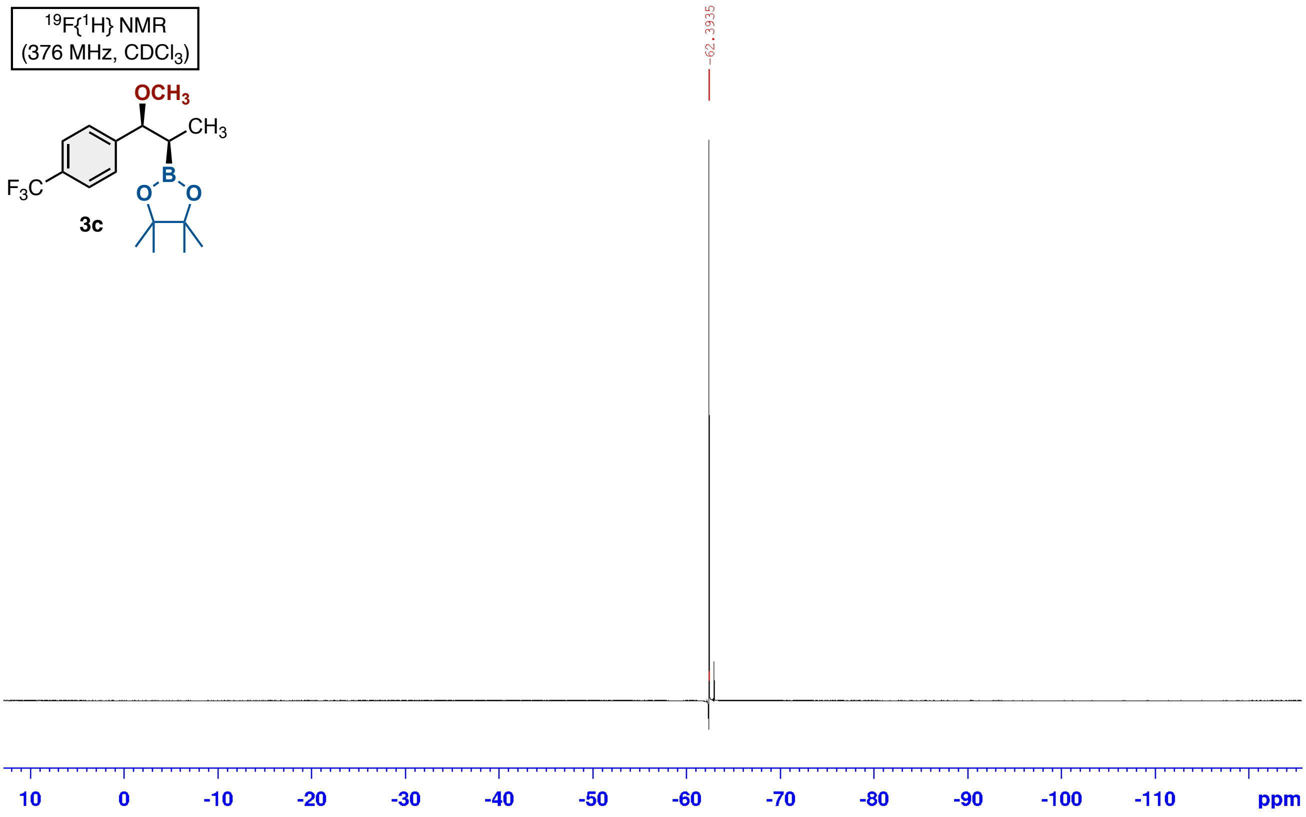

^{19}F{^{1}H} NMR
(376 MHz, CDCl$_3$)
OCH$_3$
CH$_3$
F$_3$C
B
O
O
3c
-62.3935
10
0
-10
-20
-30
-40
-50
-60
-70
-80
-90
-100
-110
ppm

[^{1}H, $^{13}C\{^{1}H\}$, and ^{11}B NMR Spectra of **3d**]

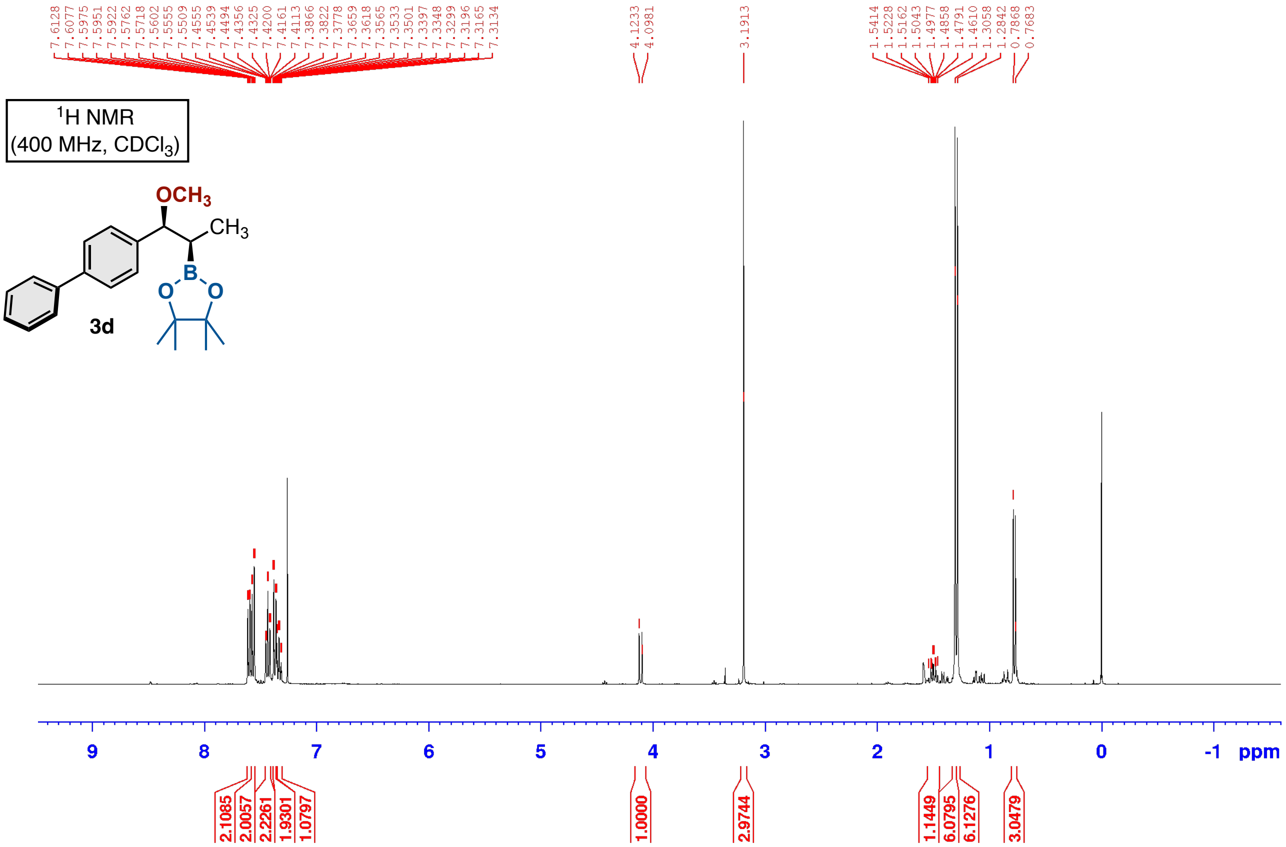

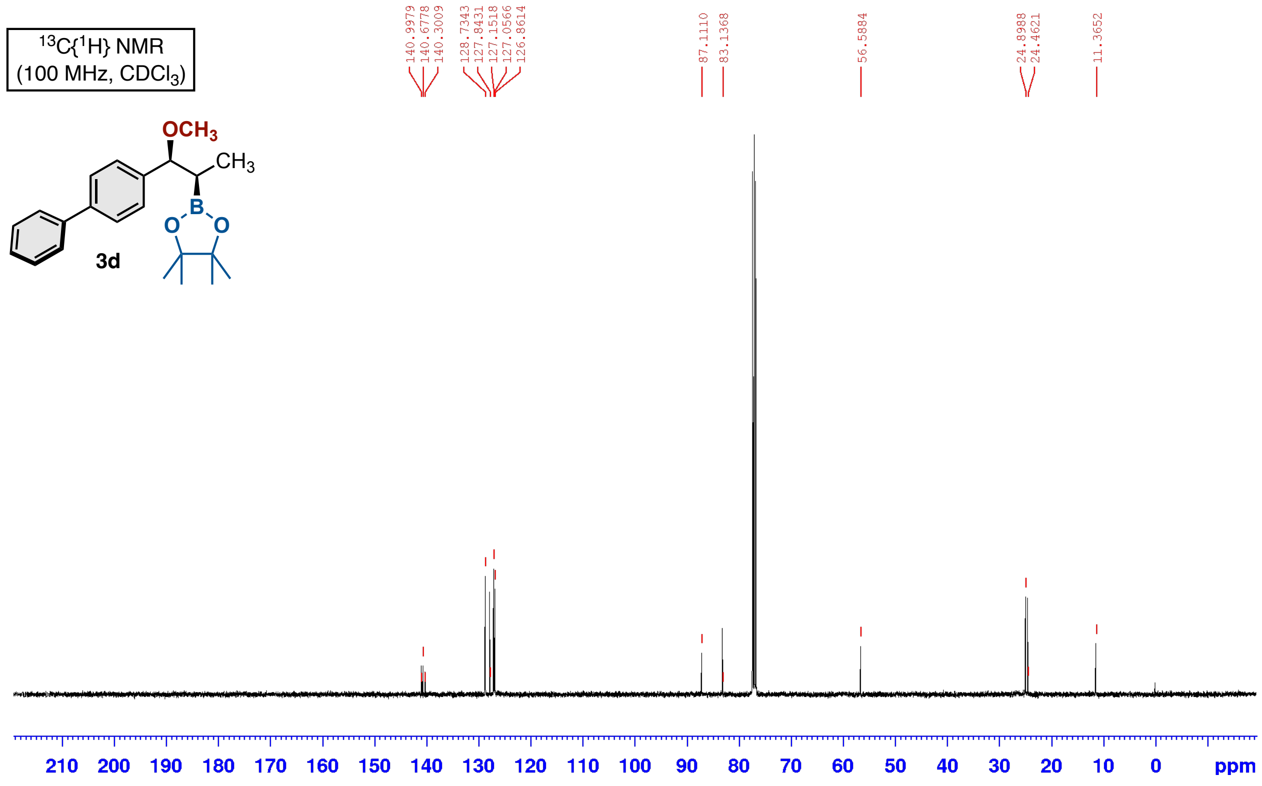

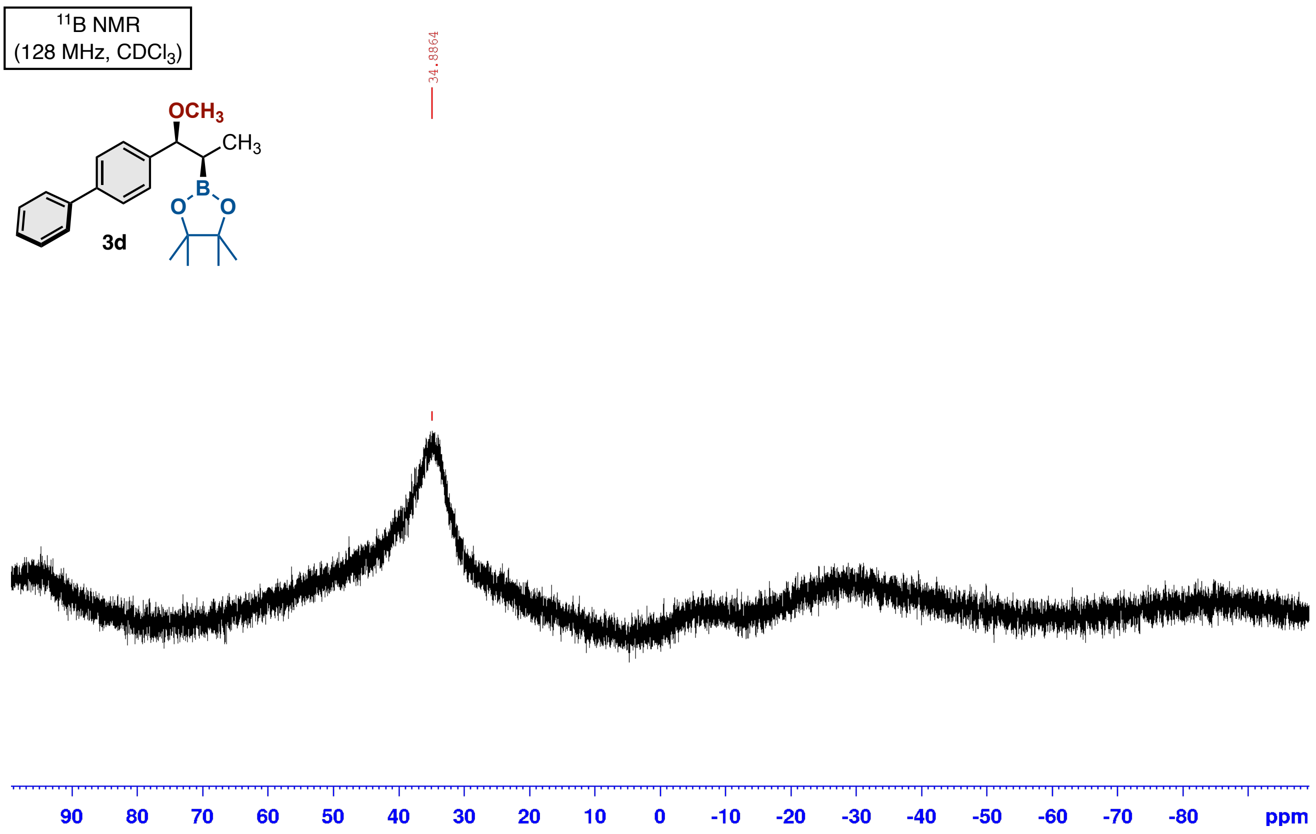

¹¹B NMR
(128 MHz, CDCl$_3$)
34.8864
OCH$_3$
CH$_3$
B
O
O
3d
90 80 70 60 50 40 30 20 10 0 -10 -20 -30 -40 -50 -60 -70 -80 ppm

[^{1}H, ^{13}C{^{1}H}, and ^{11}B NMR Spectra of **3e**]

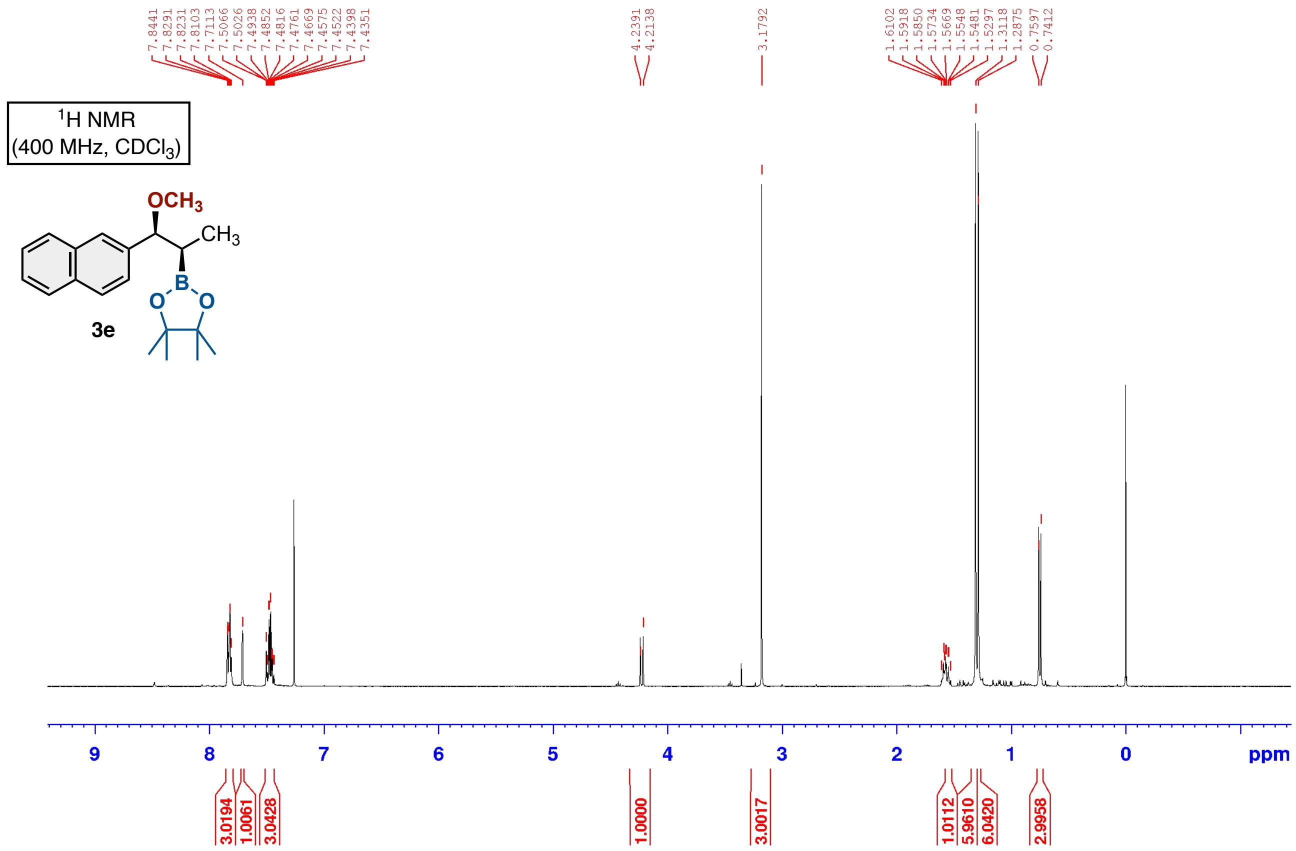

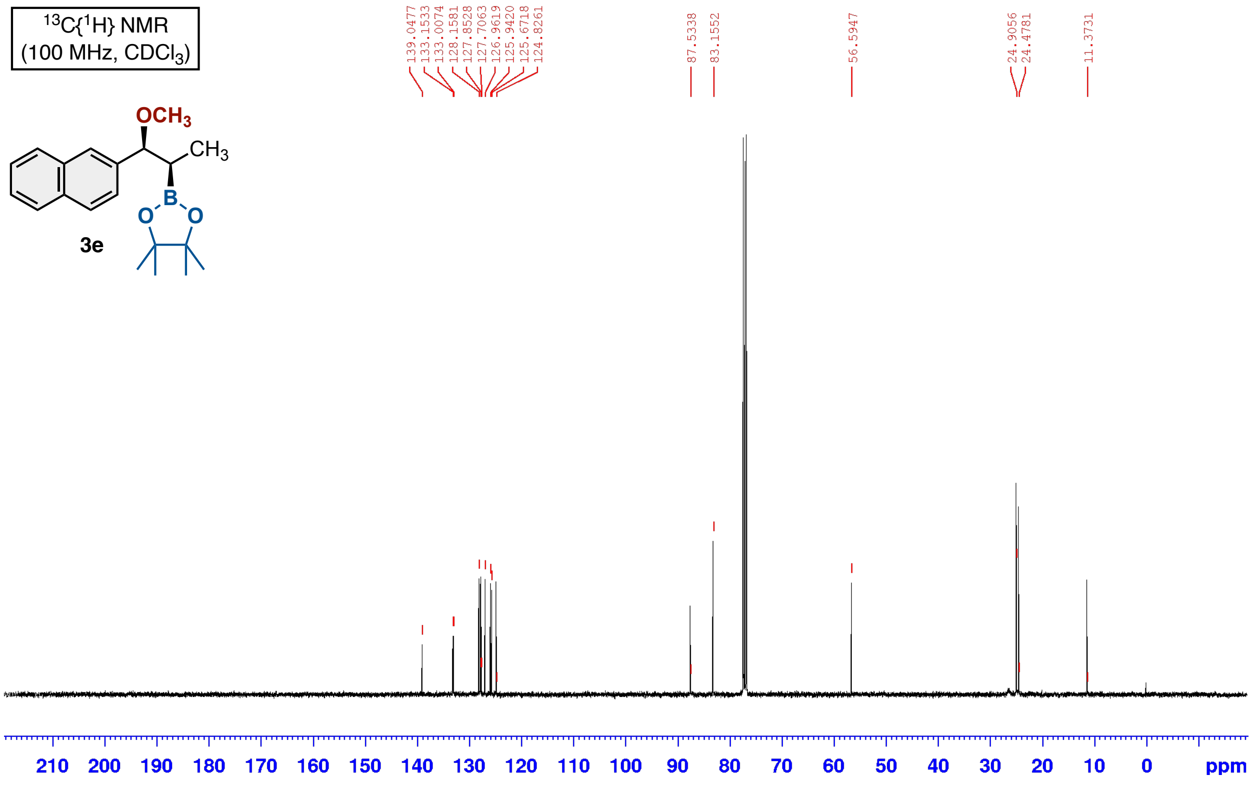

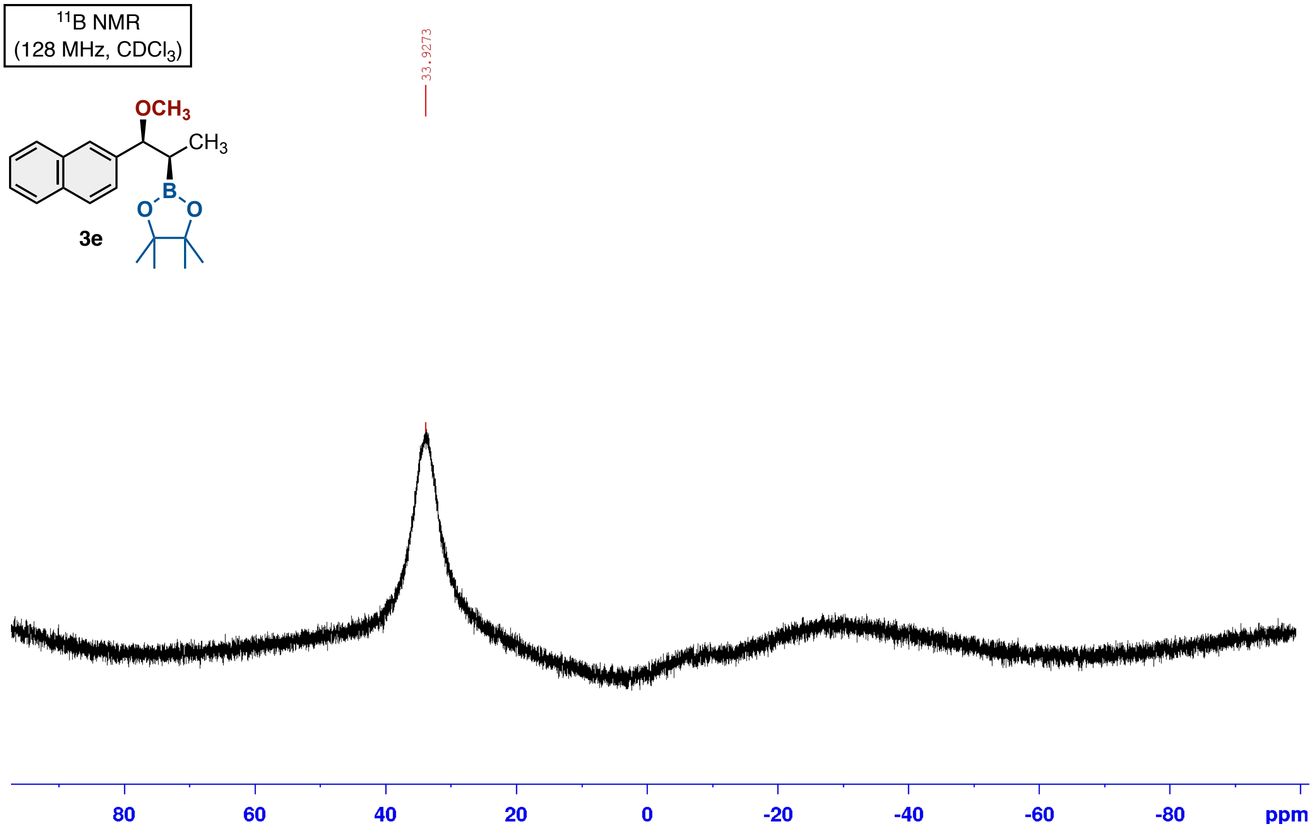

11B NMR
(128 MHz, CDCl3)
33.9273
OCH3
CH3
B
O
O
3e
80
60
40
20
0
-20
-40
-60
-80
ppm

[^{1}H, ^{13}C{^{1}H}, and ^{11}B NMR Spectra of **3f**]

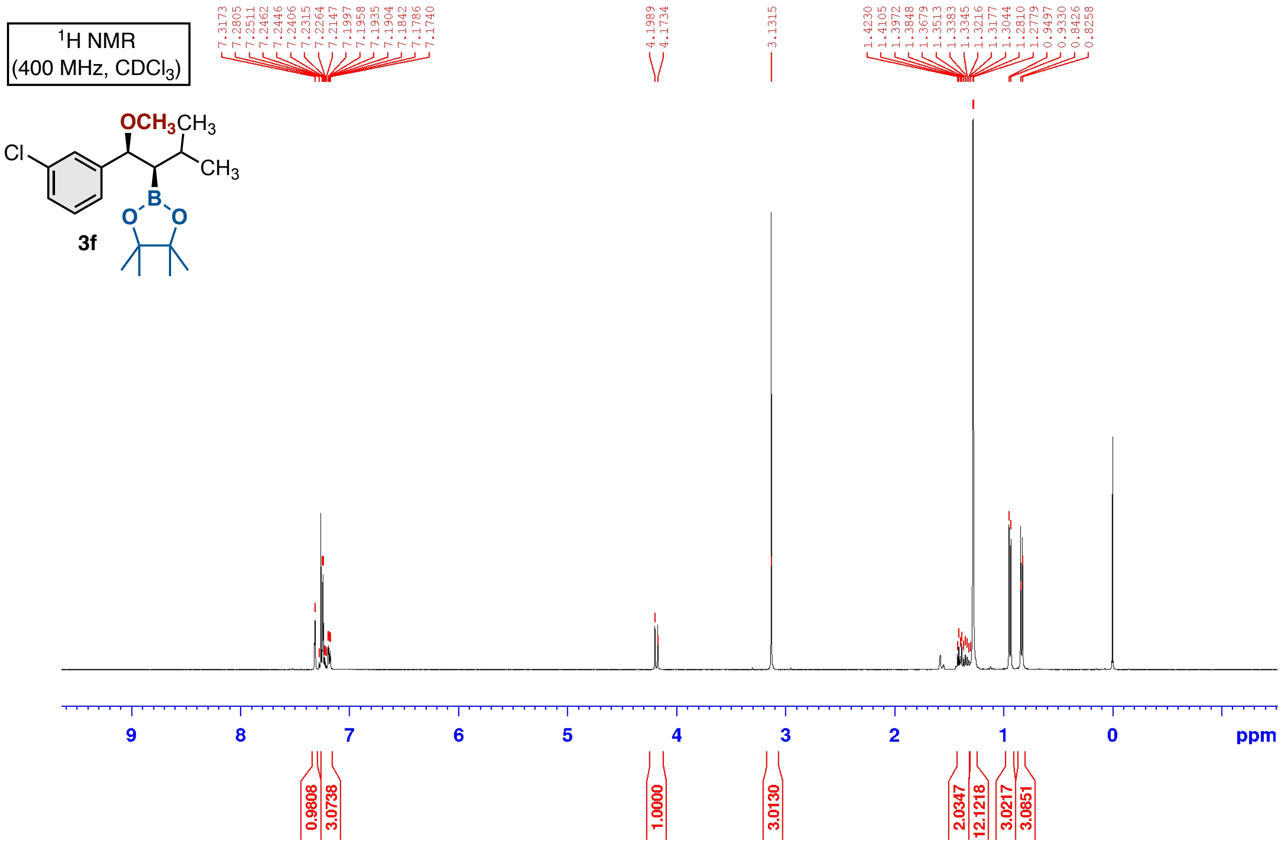

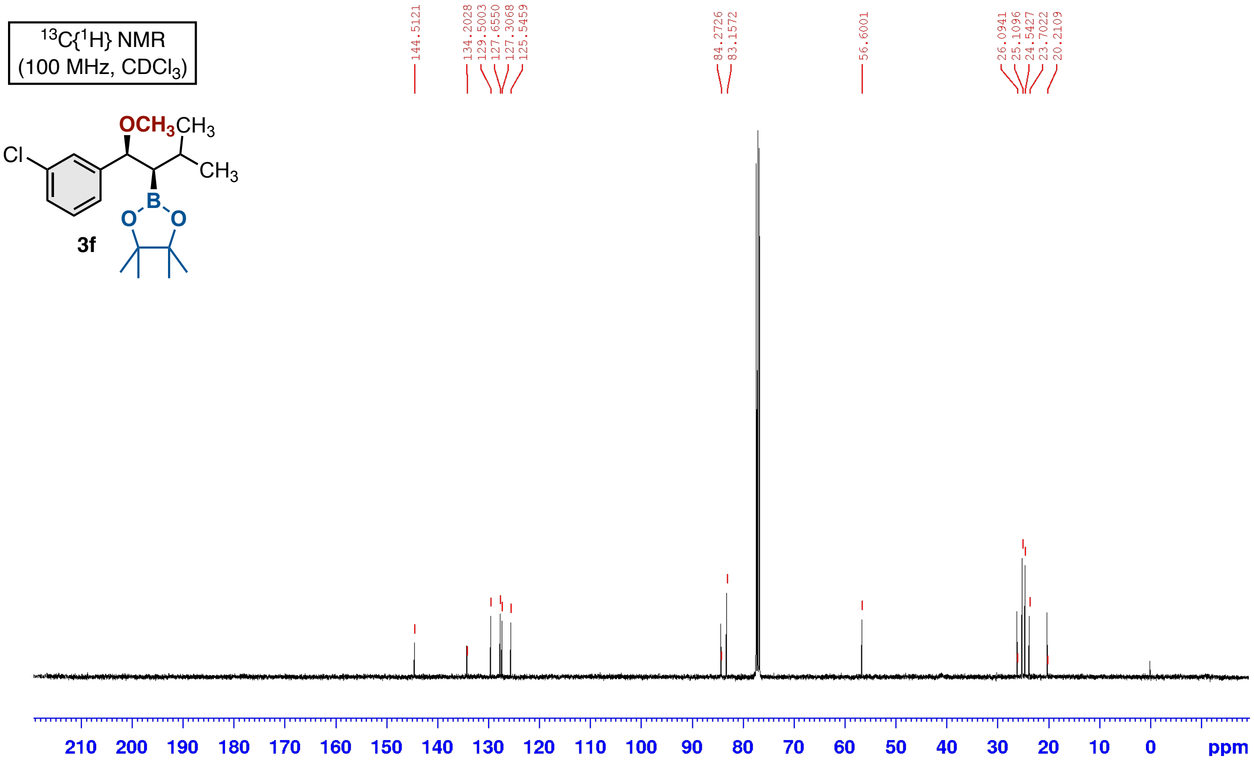

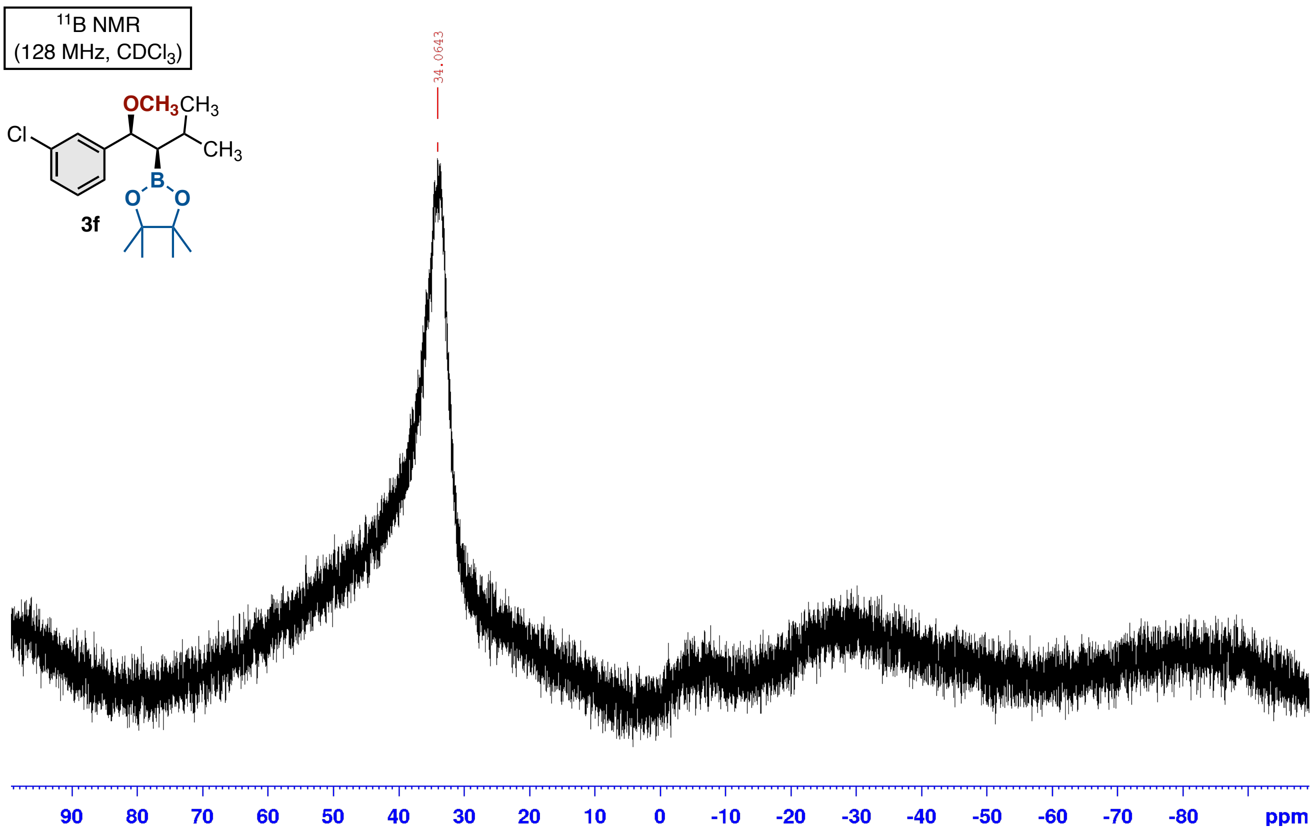

^{11}B NMR
(128 MHz, CDCl$_3$)
OCH$_3$
CH$_3$
Cl
CH$_3$
B
O
O
3f
34.0643
90 80 70 60 50 40 30 20 10 0 -10 -20 -30 -40 -50 -60 -70 -80 ppm

[^{1}H, ^{13}C{^{1}H}, and ^{11}B NMR Spectra of **3g**]

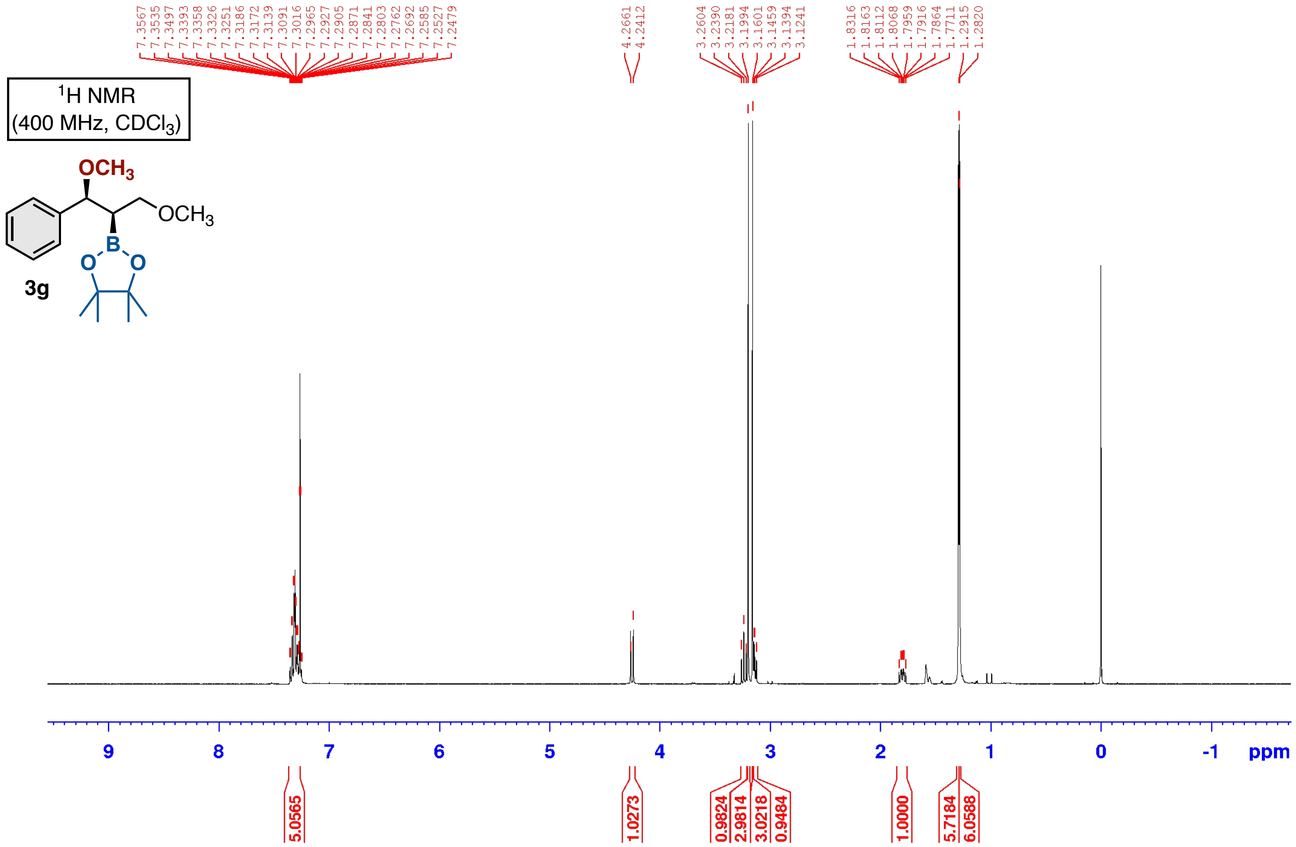

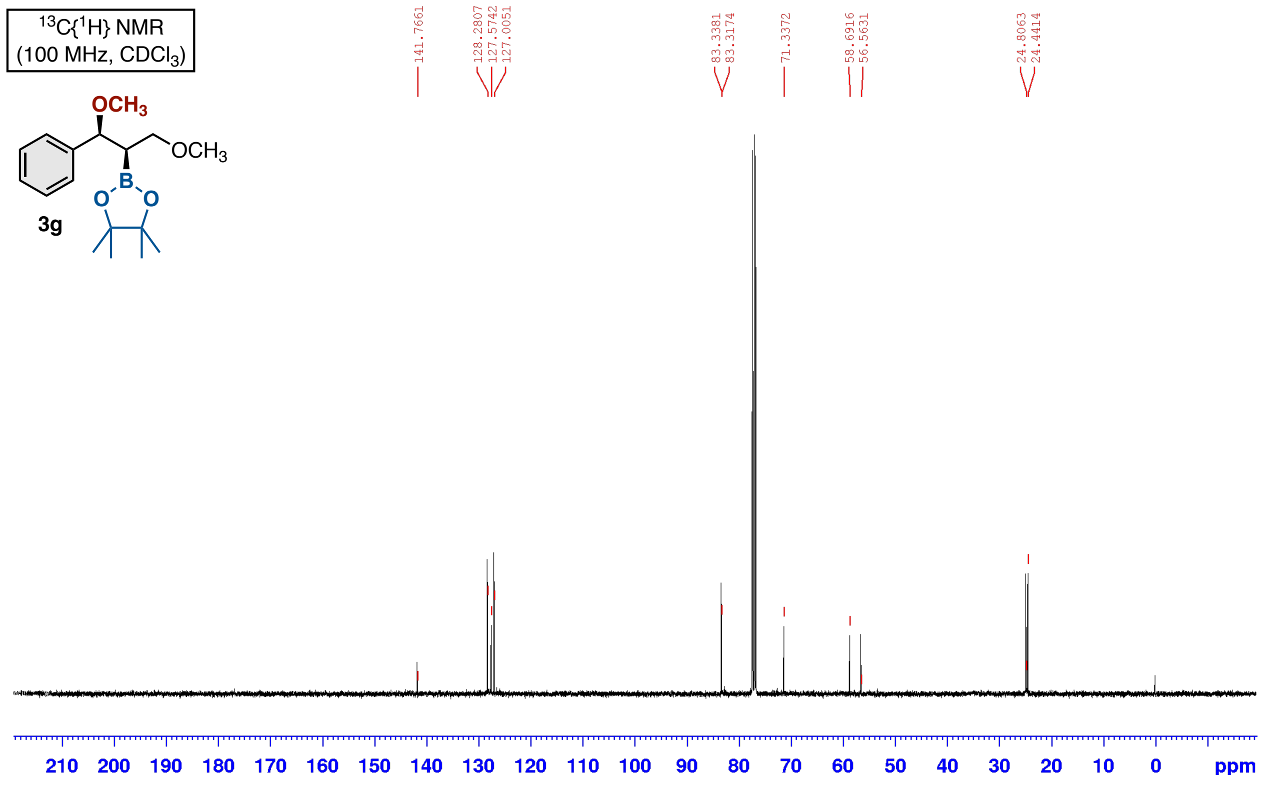

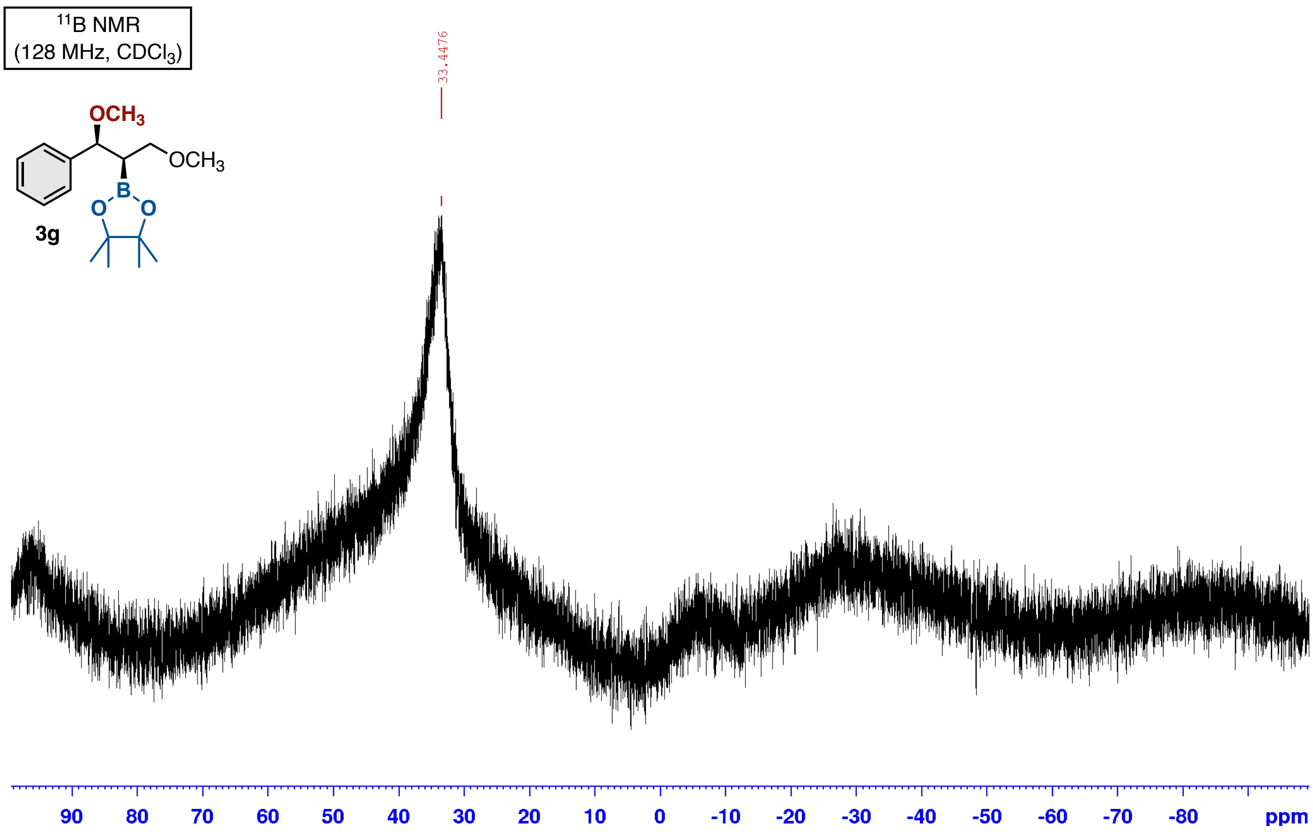

11B NMR
(128 MHz, CDCl3)
33.4476
OCH3
OCH3
B
O
O
3g
90
80
70
60
50
40
30
20
10
0
-10
-20
-30
-40
-50
-60
-70
-80
ppm

[^{1}H, $^{13}C\{^{1}H\}$, and ^{11}B NMR Spectra of **3h**]

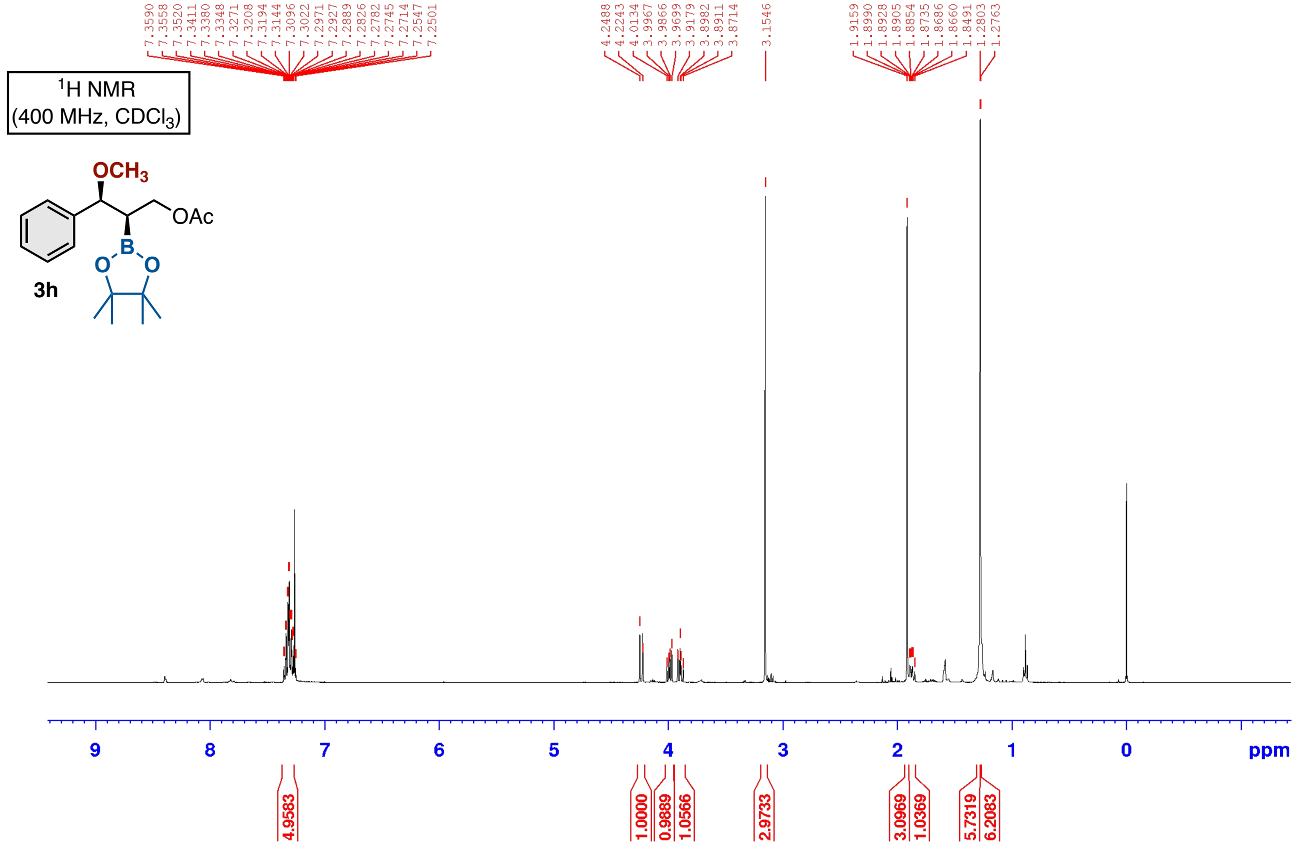

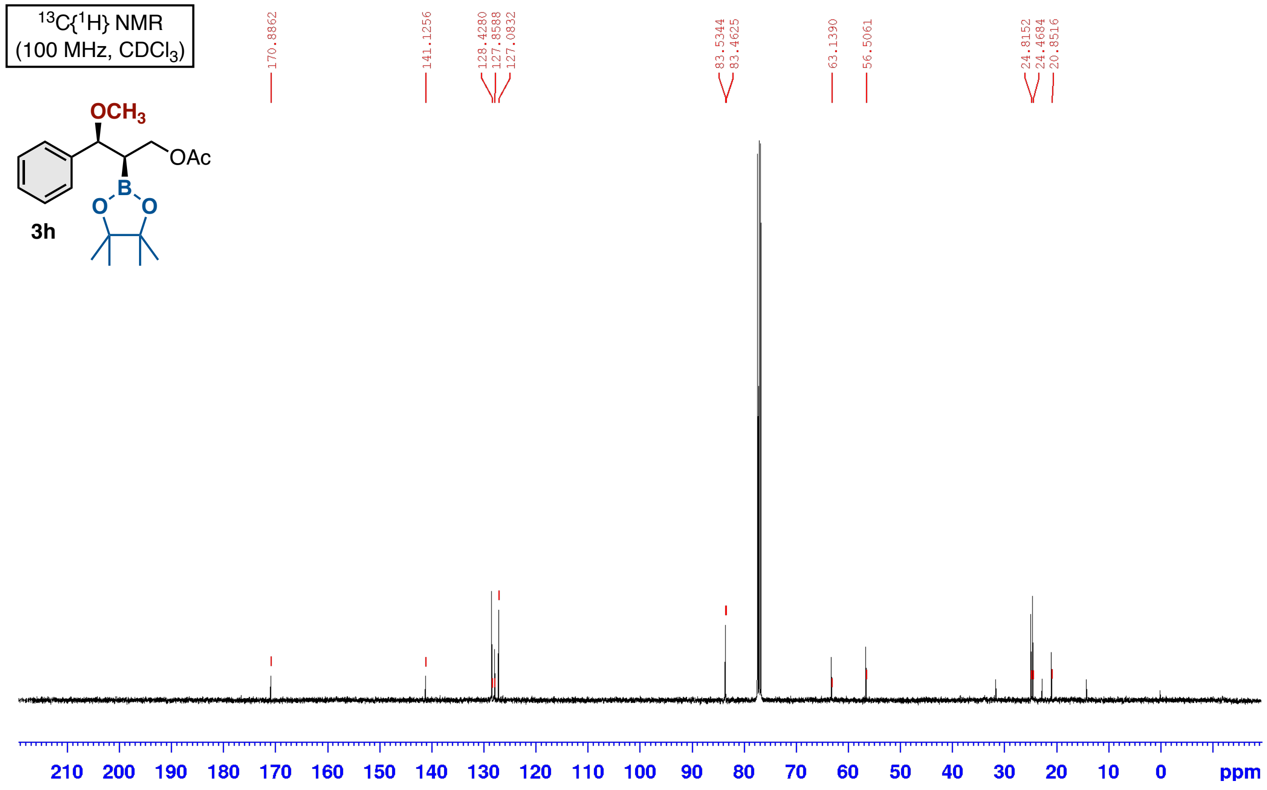

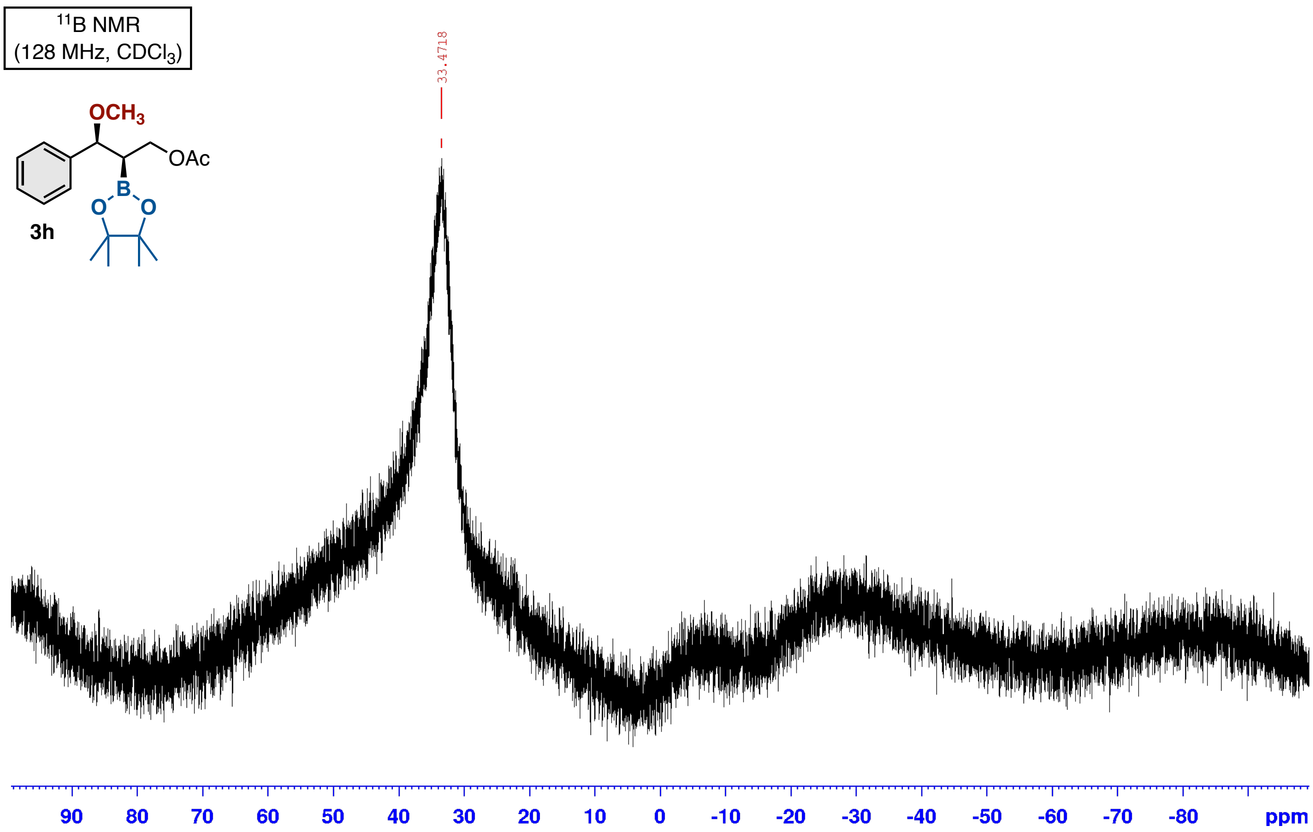

^{11}B NMR
(128 MHz, CDCl$_3$)
OCH$_3$
OAc
B
O
O
3h
33.4718
90
80
70
60
50
40
30
20
10
0
-10
-20
-30
-40
-50
-60
-70
-80
ppm

[^{1}H, ^{13}C{^{1}H}, and ^{11}B NMR Spectra of **3i**]

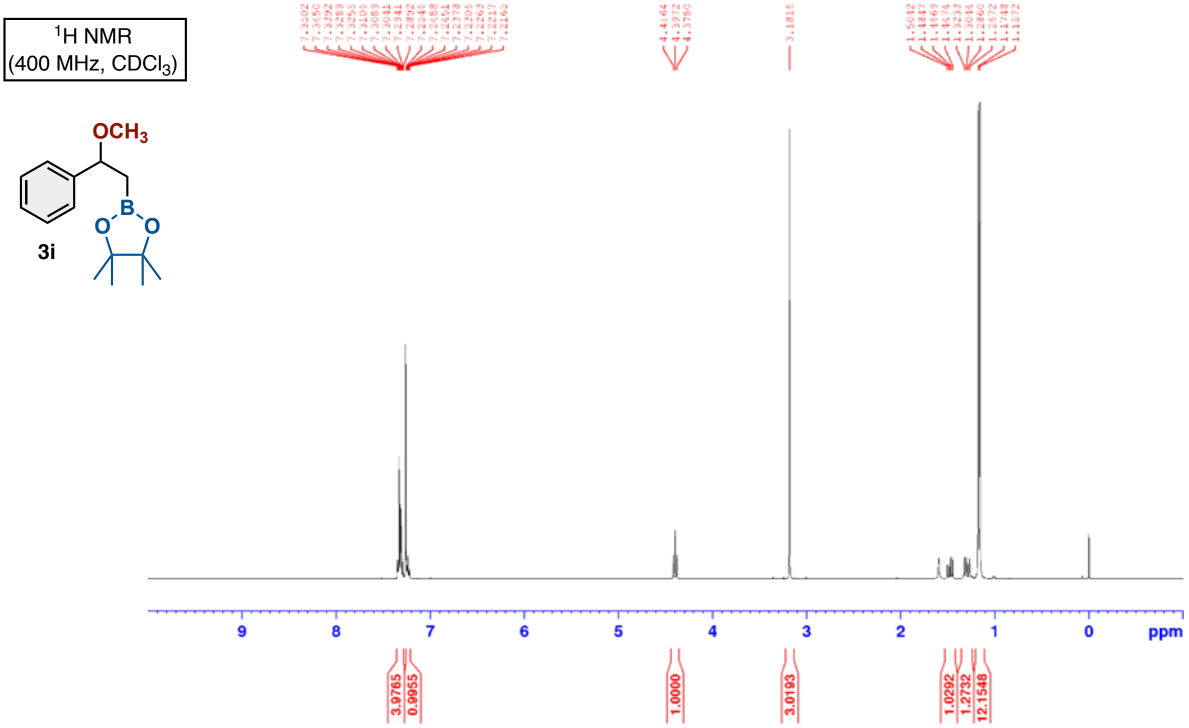

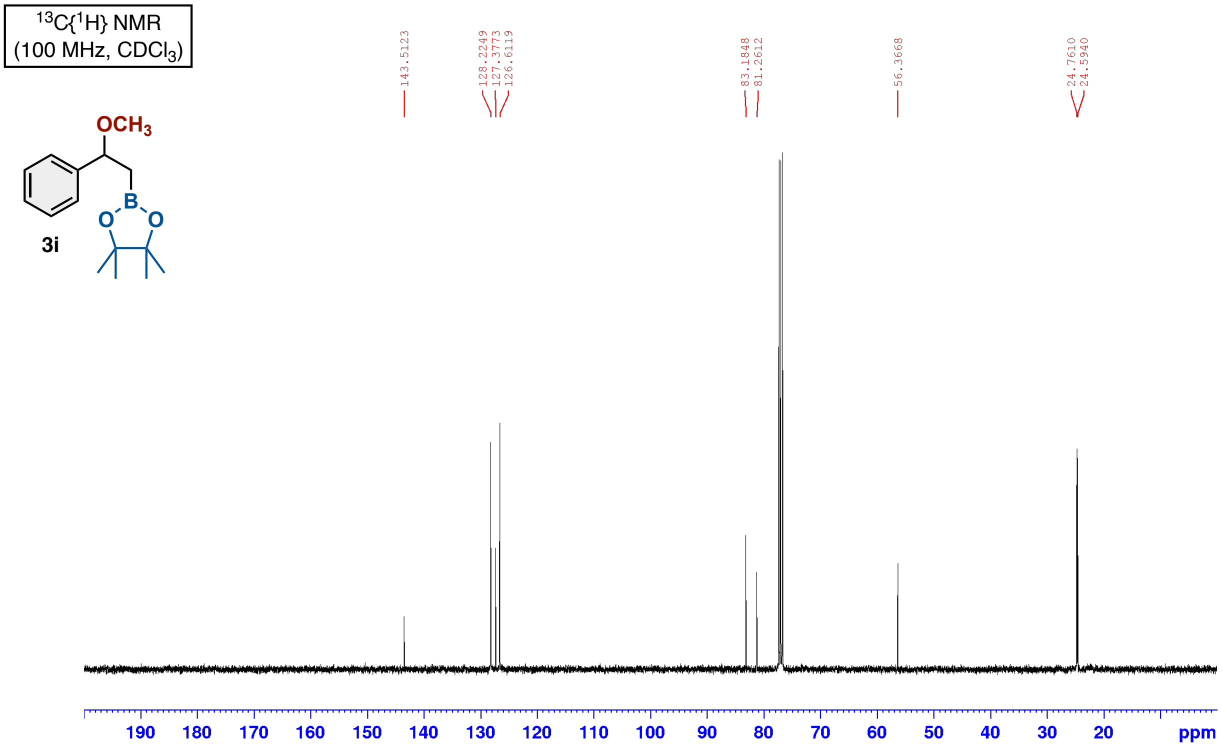

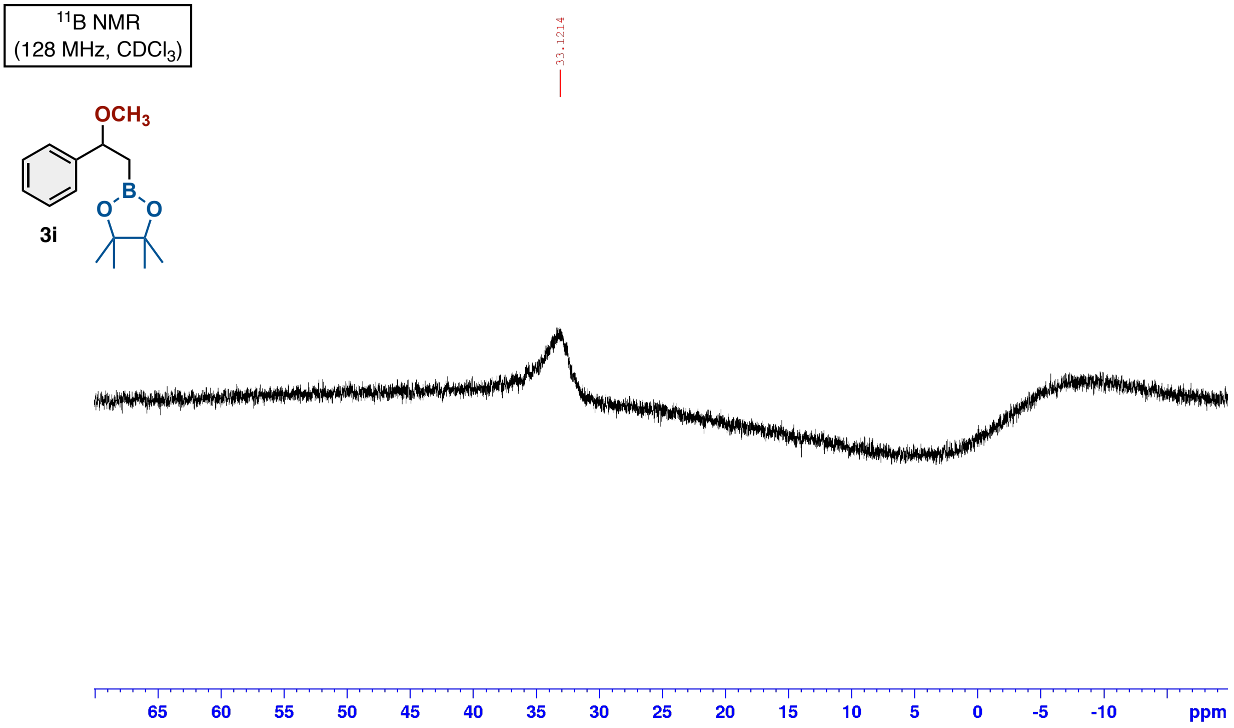

^{11}B NMR
(128 MHz, $CDCl_3$)
33.1214
OCH3
B
O
O
3i
65
60
55
50
45
40
35
30
25
20
15
10
5
0
-5
-10
ppm

[^{1}H, ^{13}C{^{1}H}, and ^{11}B NMR Spectra of **3j**]

^{1}H NMR
(400 MHz, $CDCl_3$)

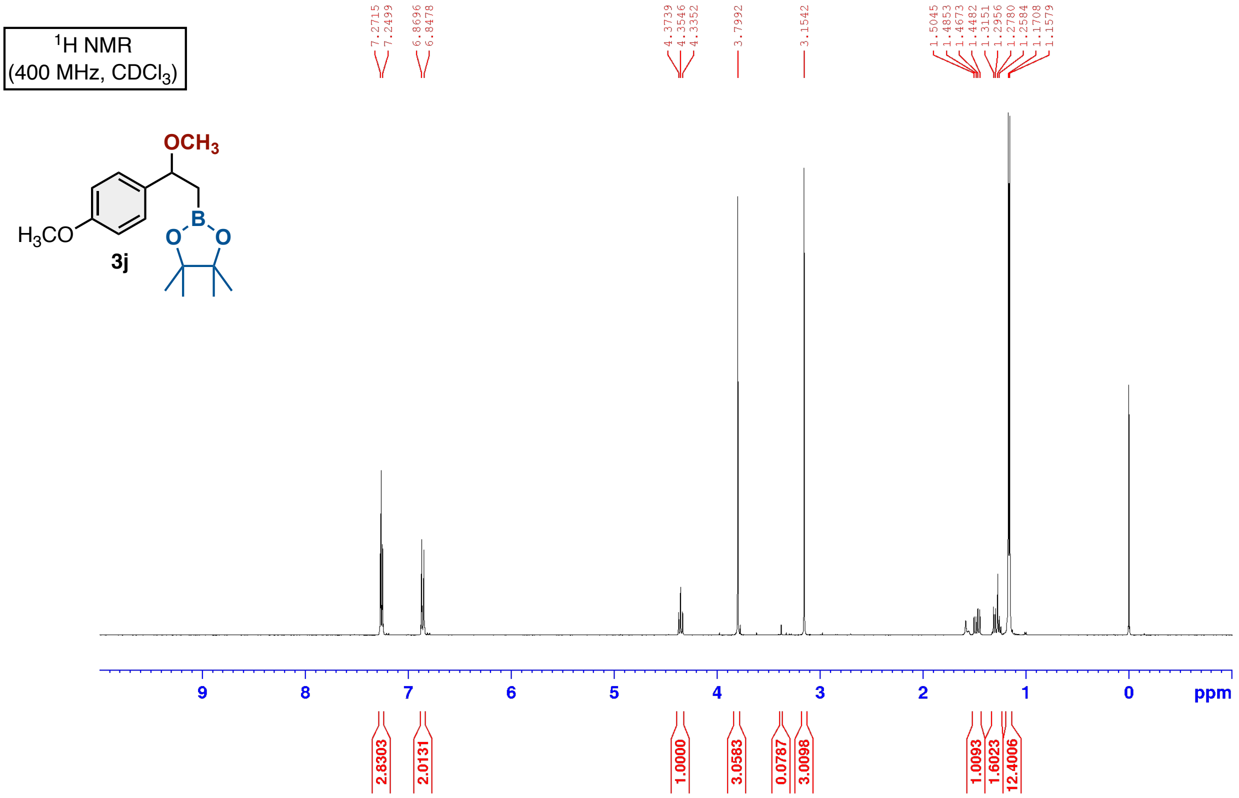

^{13}C{^{1}H} NMR
(100 MHz, $CDCl_3$)

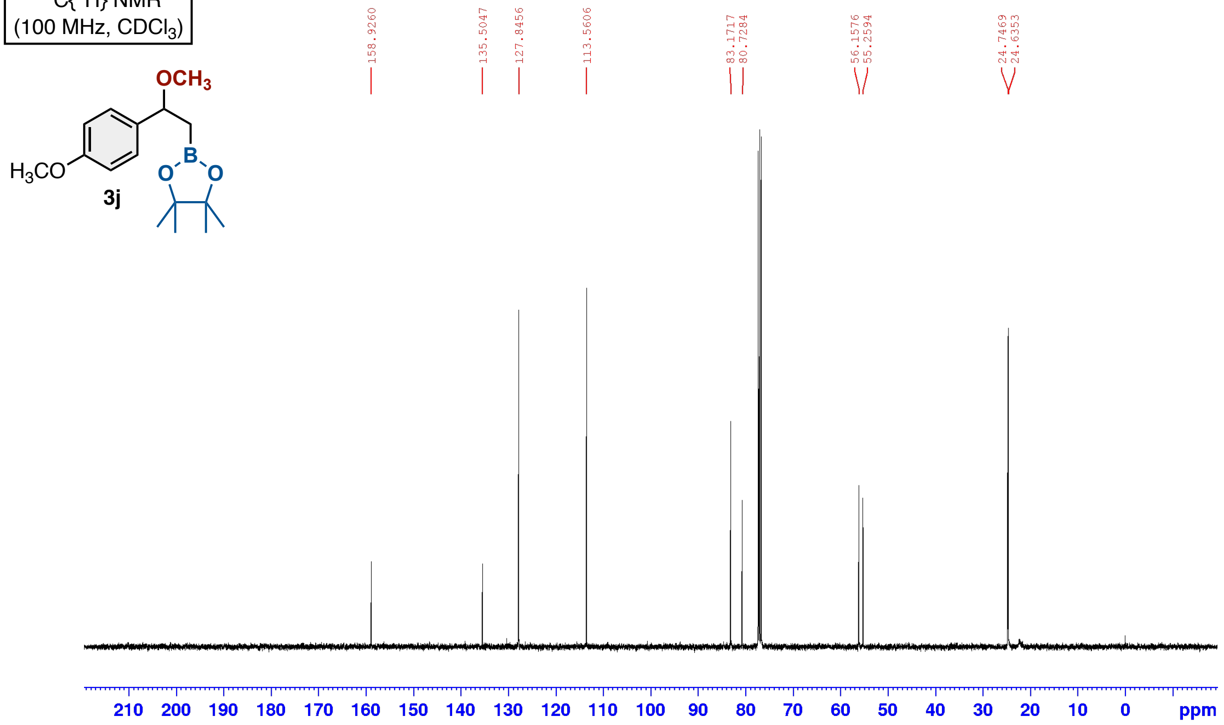

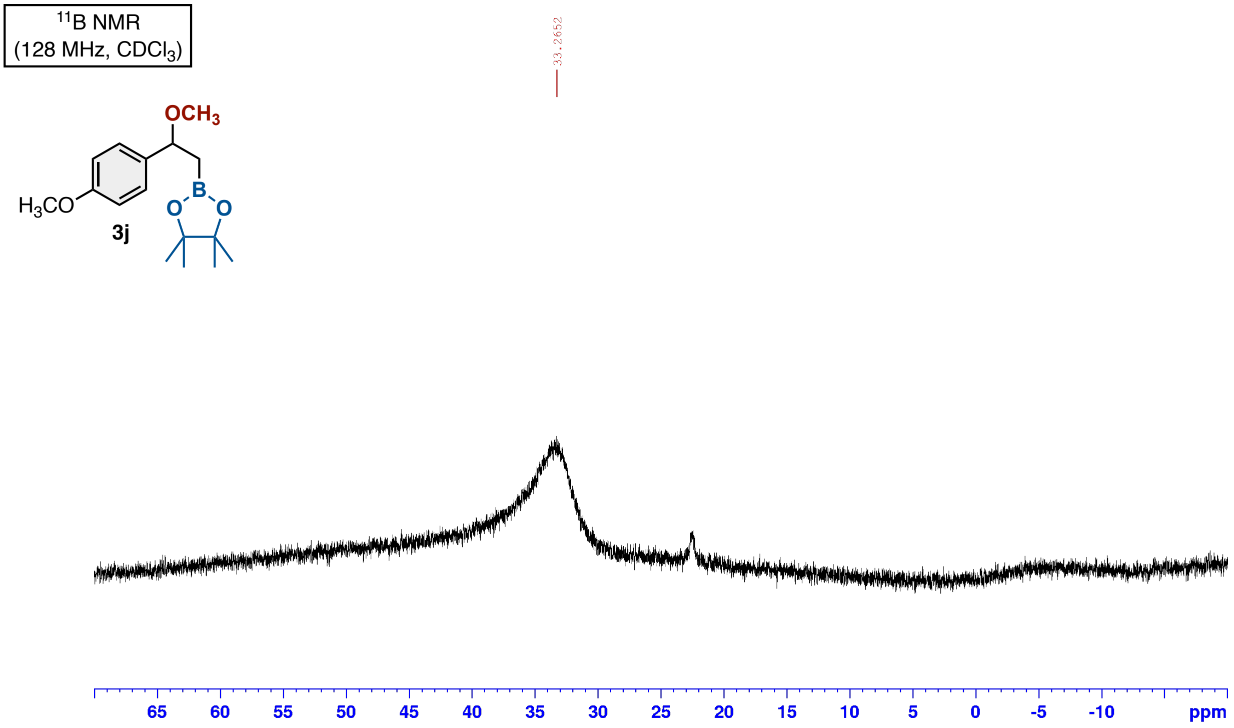

^{11}B NMR
(128 MHz, $CDCl_3$)
33.2652
OCH_3
H_3CO
3j
B
O
O
65
60
55
50
45
40
35
30
25
20
15
10
5
0
-5
-10
ppm

[^{1}H, ^{13}C{^{1}H}, and ^{11}B NMR Spectra of **3k**]

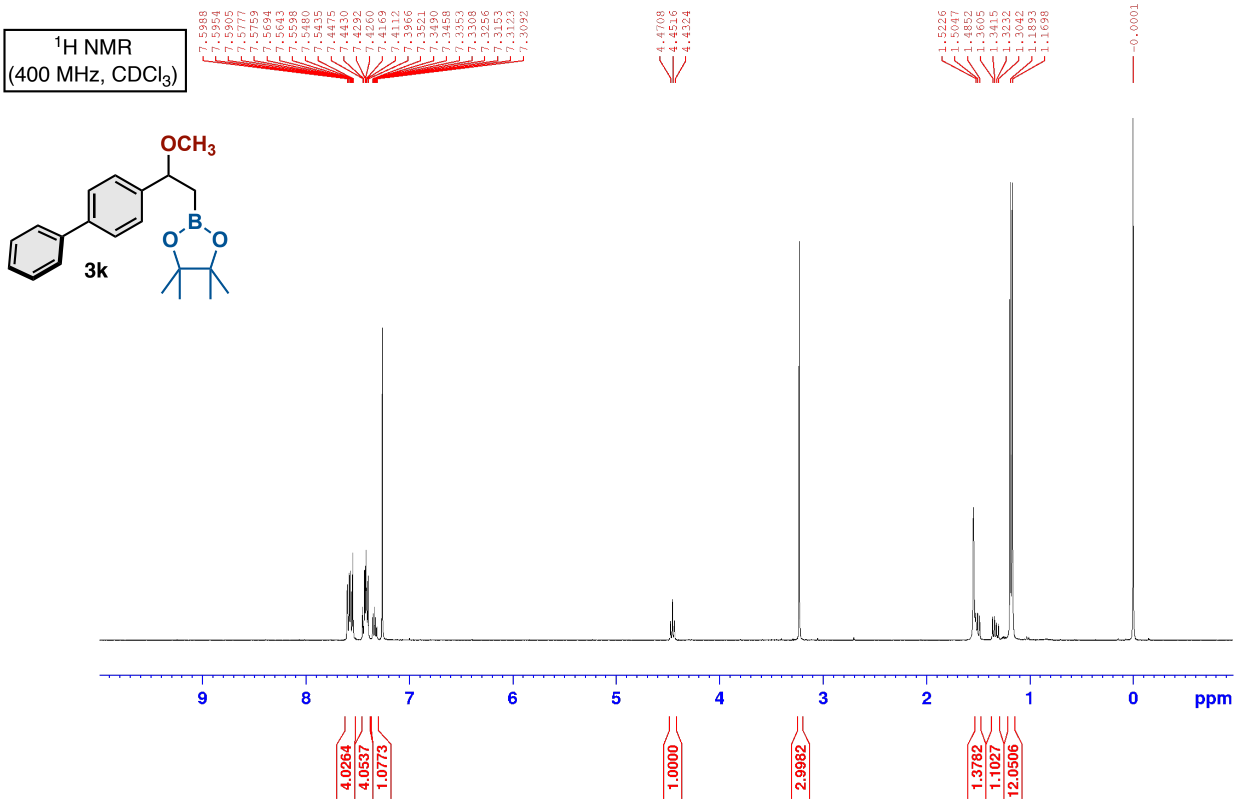

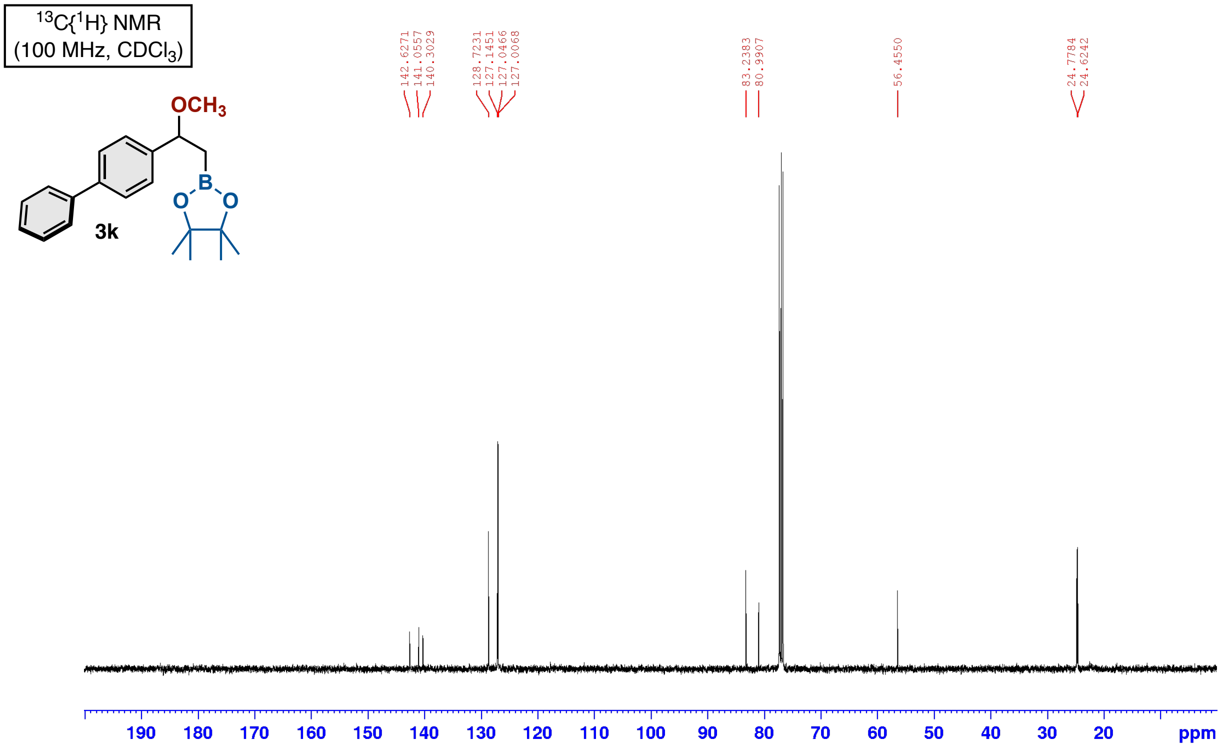

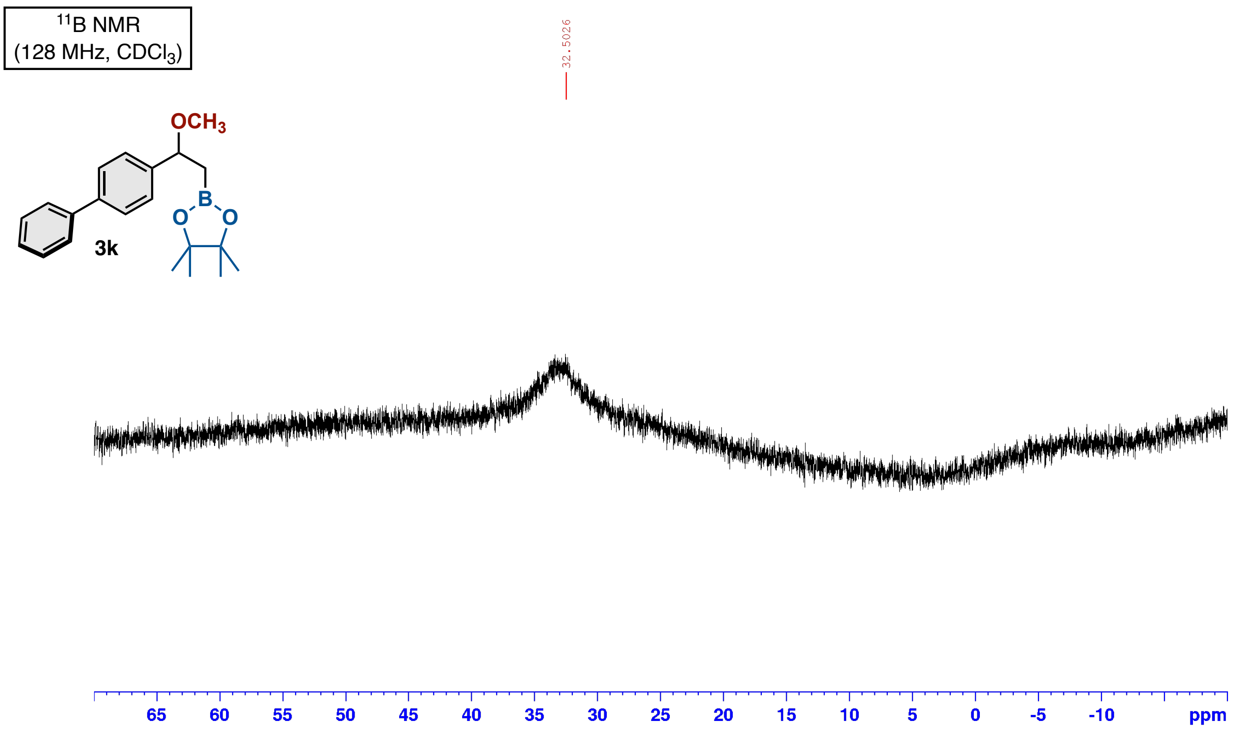

11B NMR
(128 MHz, CDCl3)
32.5026
OCH3
B
O
O
3k
65
60
55
50
45
40
35
30
25
20
15
10
5
0
-5
-10
ppm

[^{1}H, ^{13}C{^{1}H}, and ^{11}B NMR Spectra of **3l**]

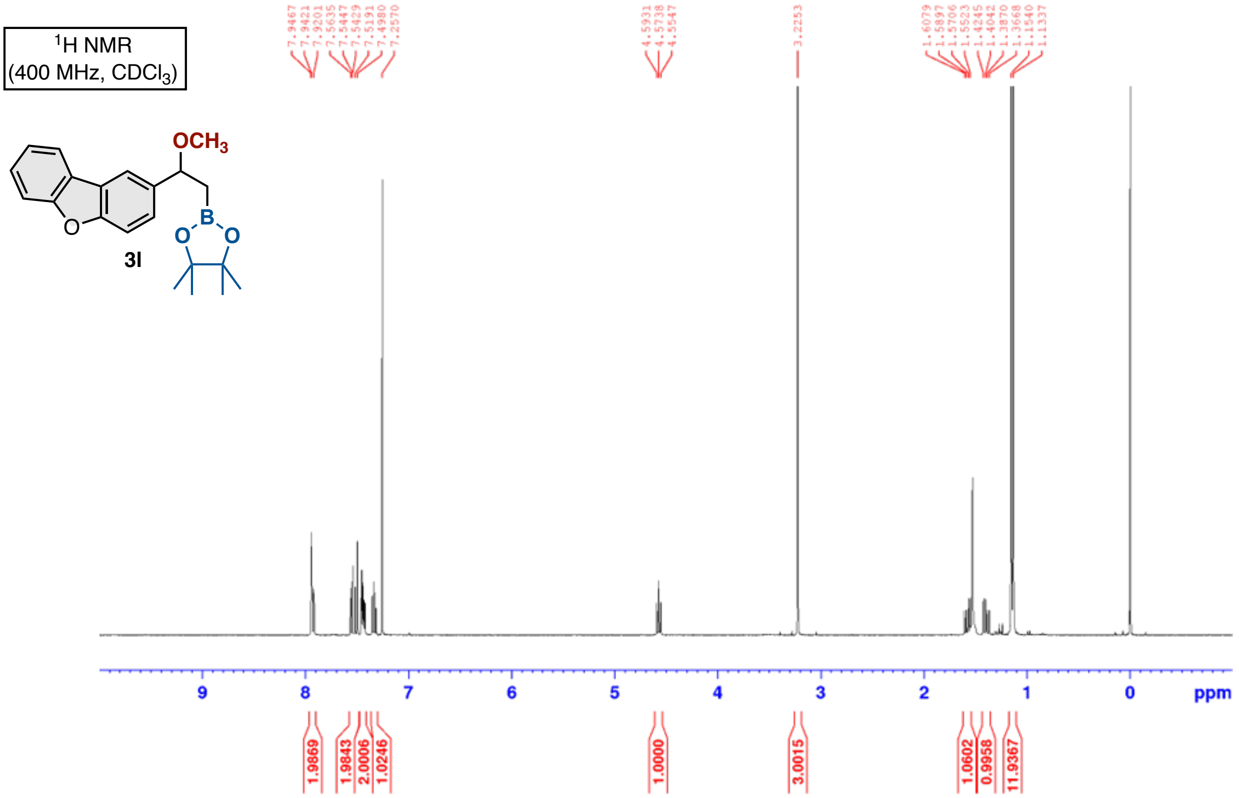

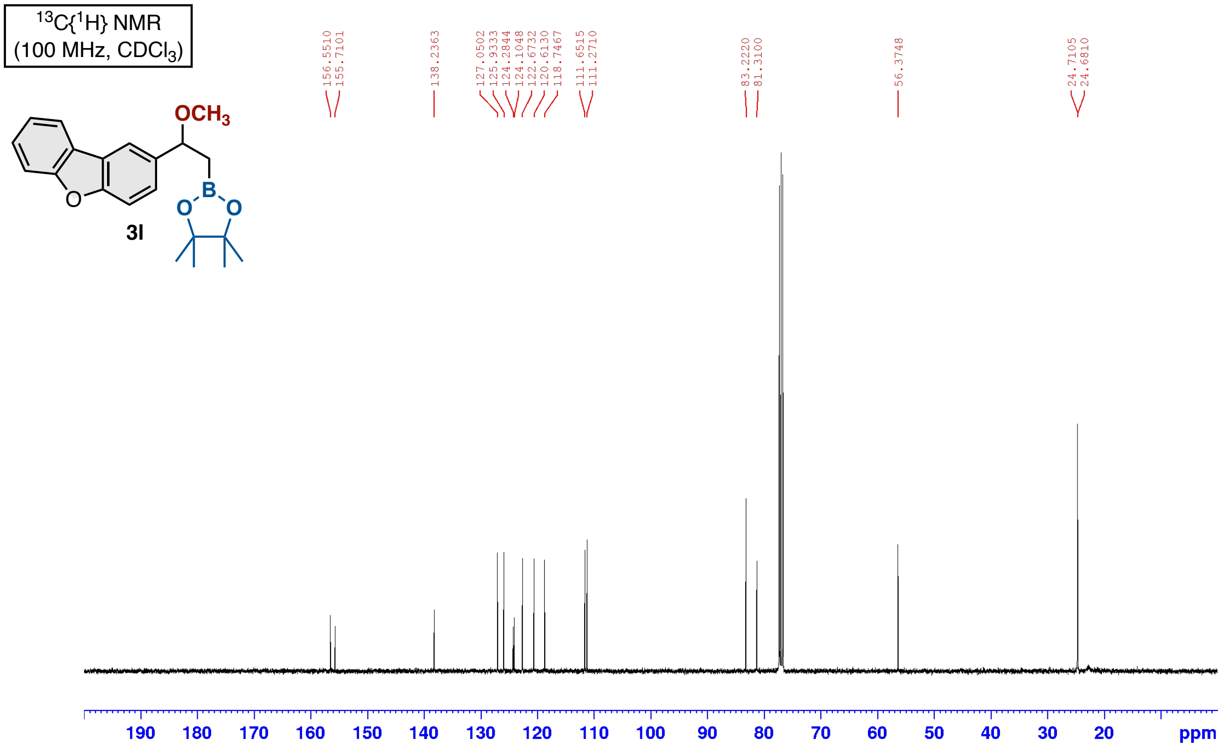

^{11}B NMR
(128 MHz, $CDCl_3$)

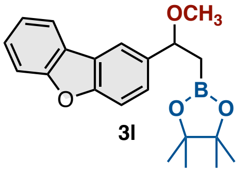

3l

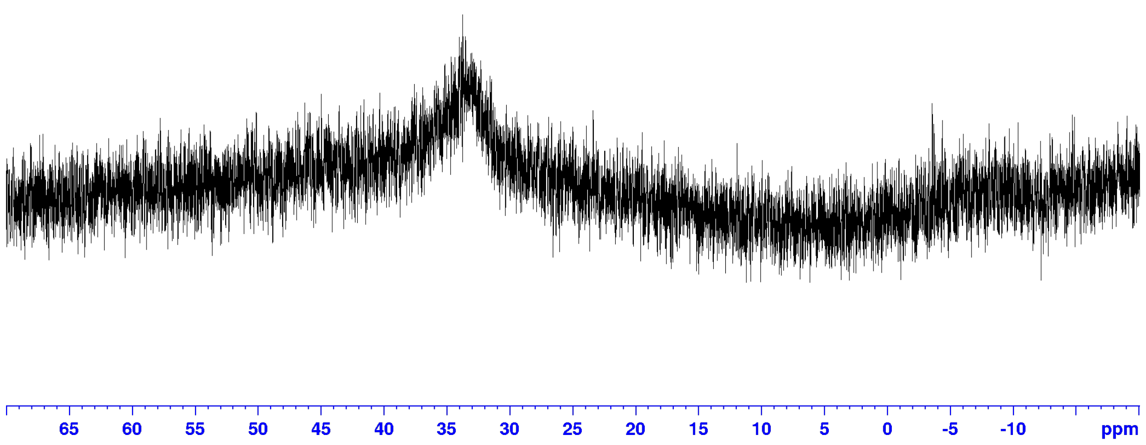

[^{1}H, ^{13}C{^{1}H}, and ^{11}B NMR Spectra of **3m**]

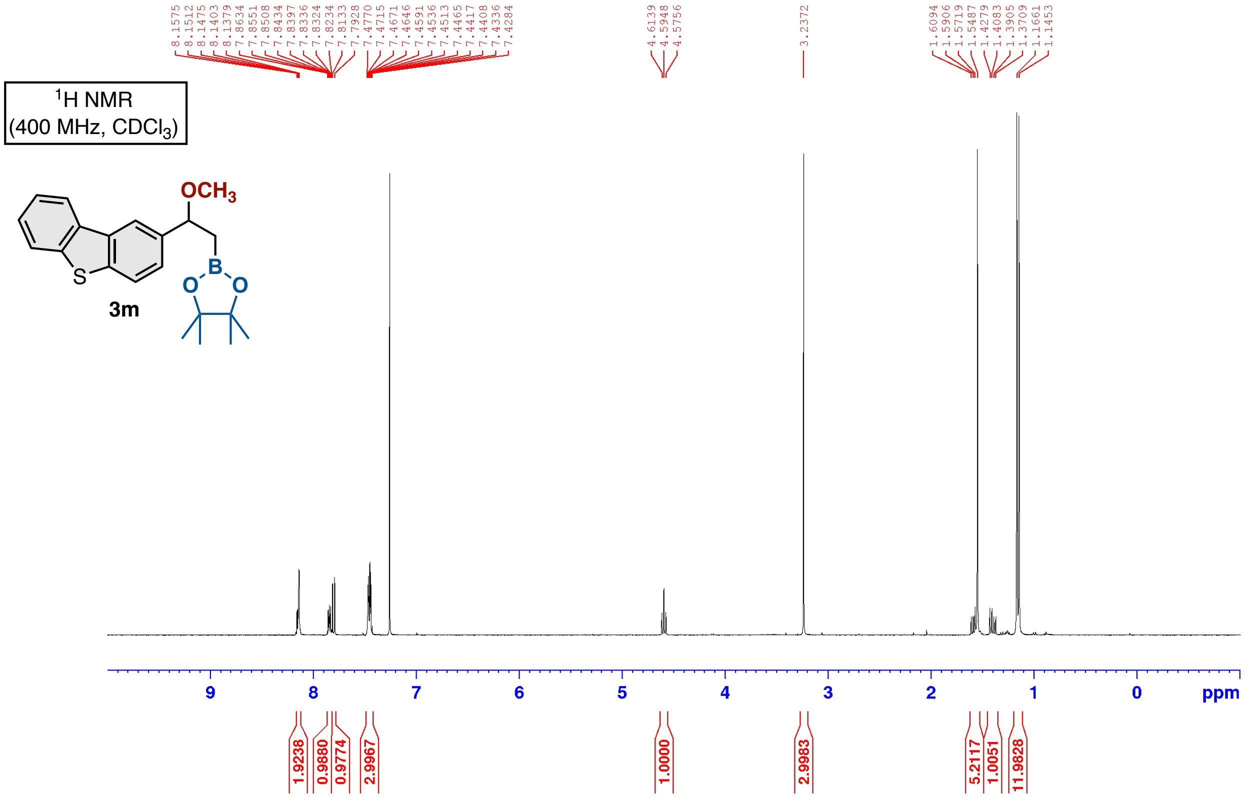

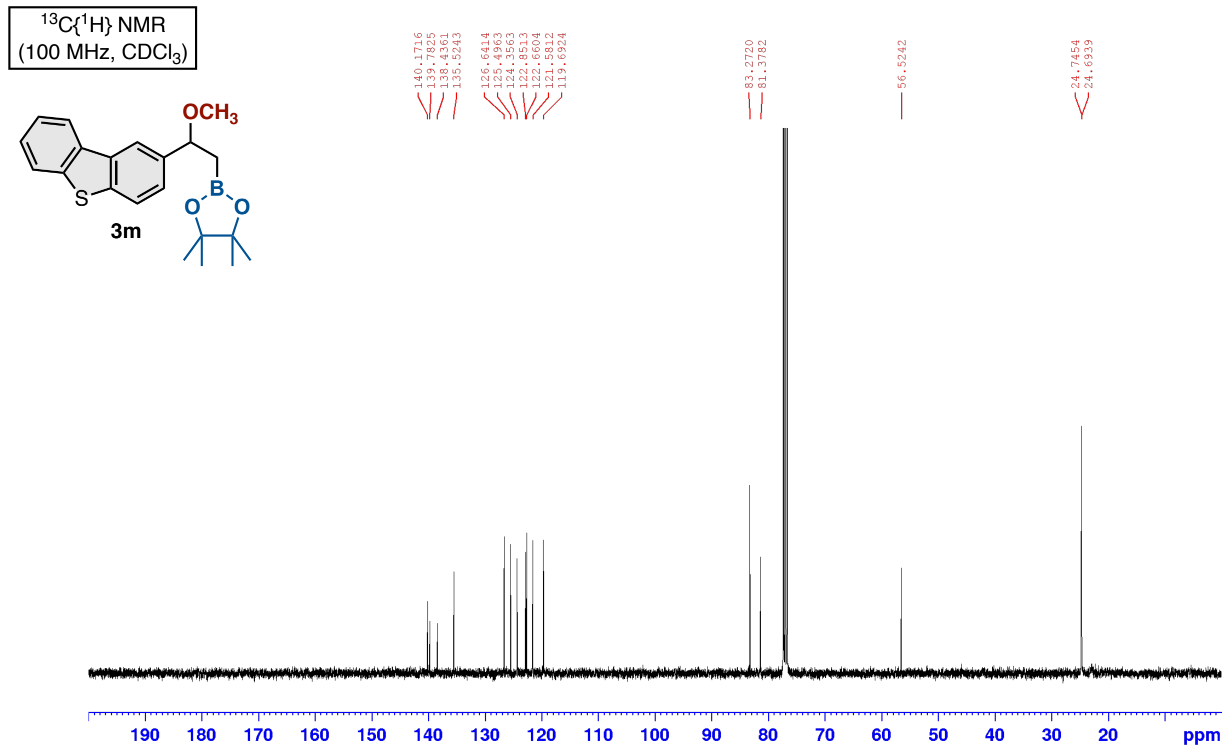

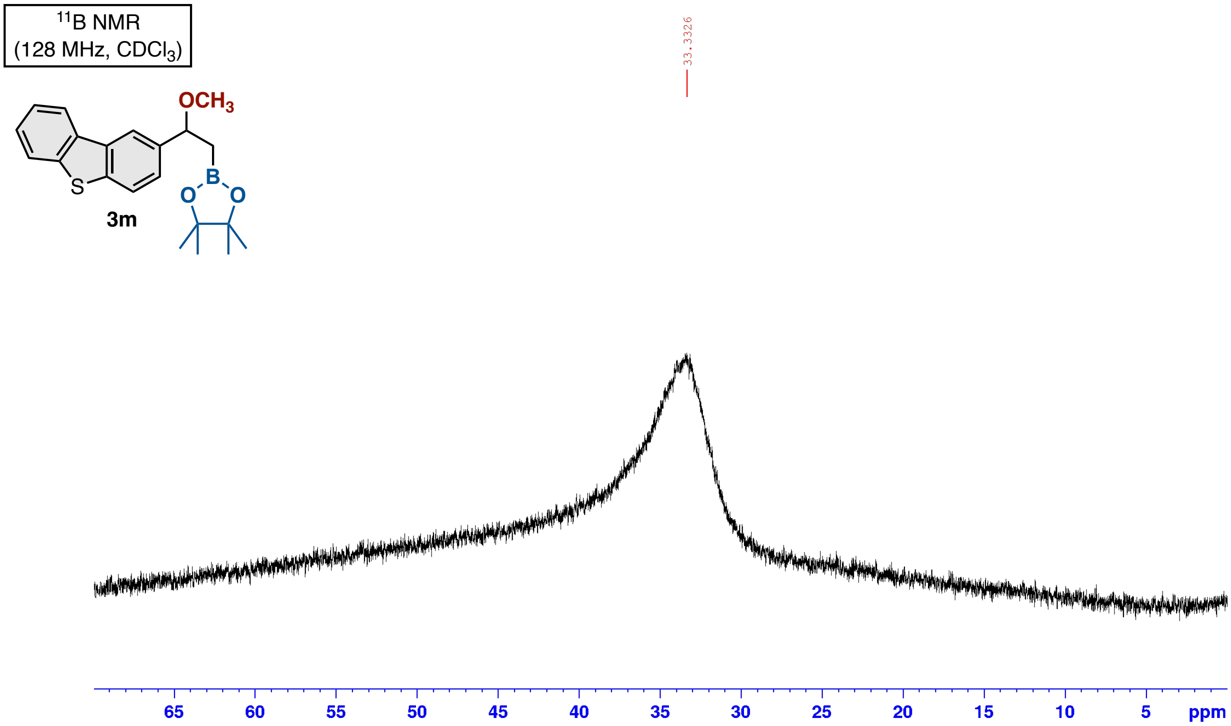

¹¹B NMR
(128 MHz, CDCl3)
OCH3
S
B
O
O
3m
33.3326
65
60
55
50
45
40
35
30
25
20
15
10
5
ppm

[^{1}H, $^{13}C\{^{1}H\}$, and ^{11}B NMR Spectra of **3n**]

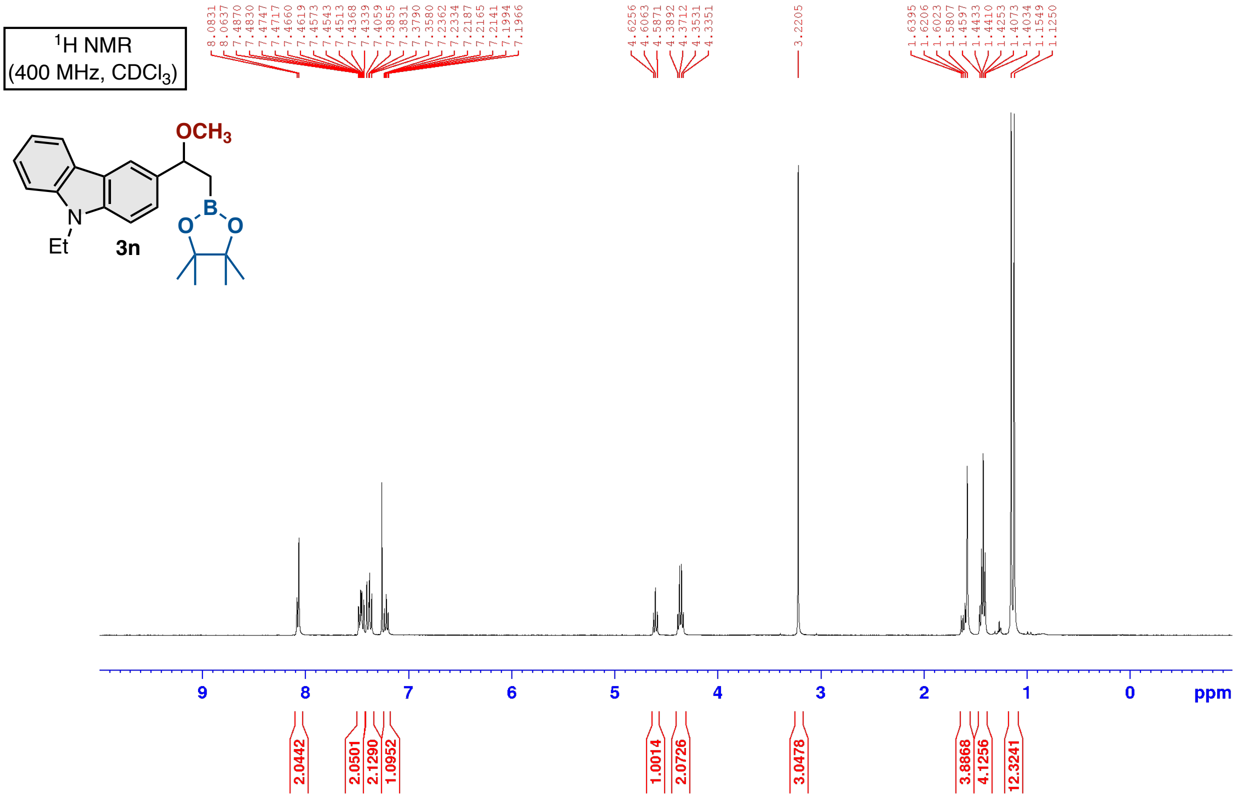

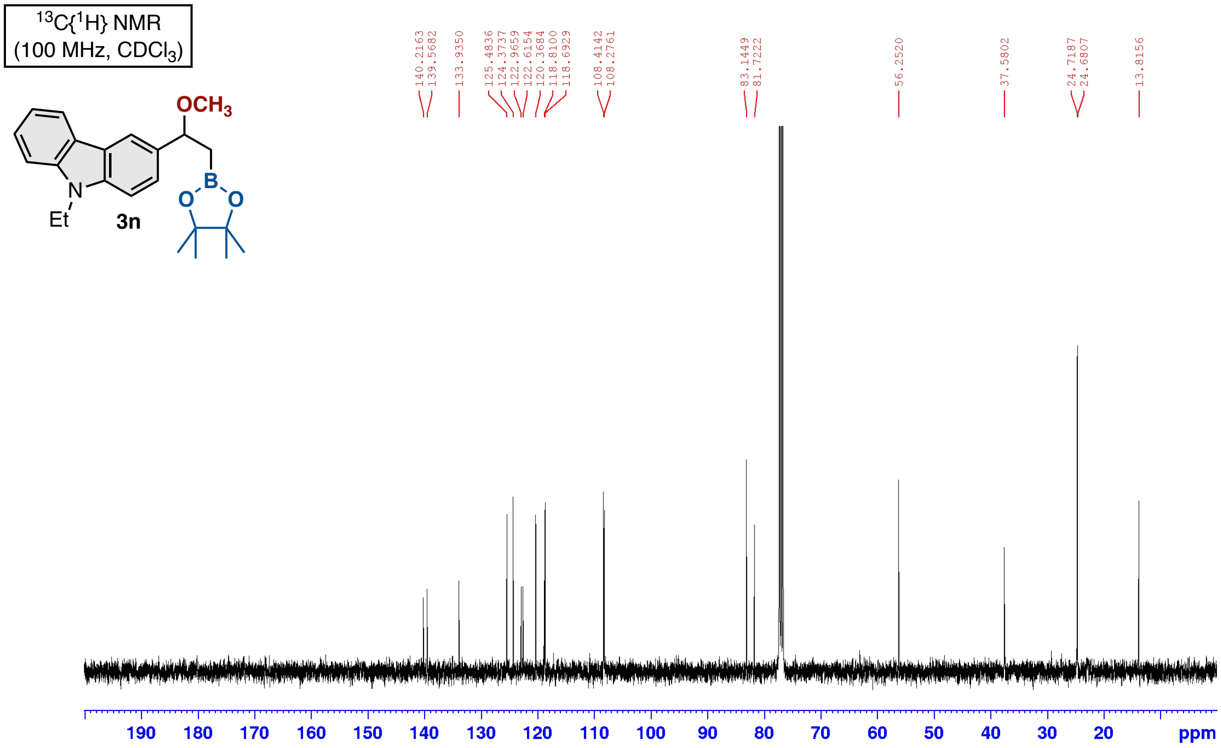

^{11}B NMR
(128 MHz, $CDCl_3$)

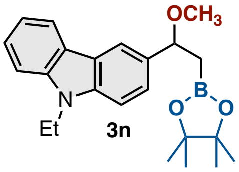

3n

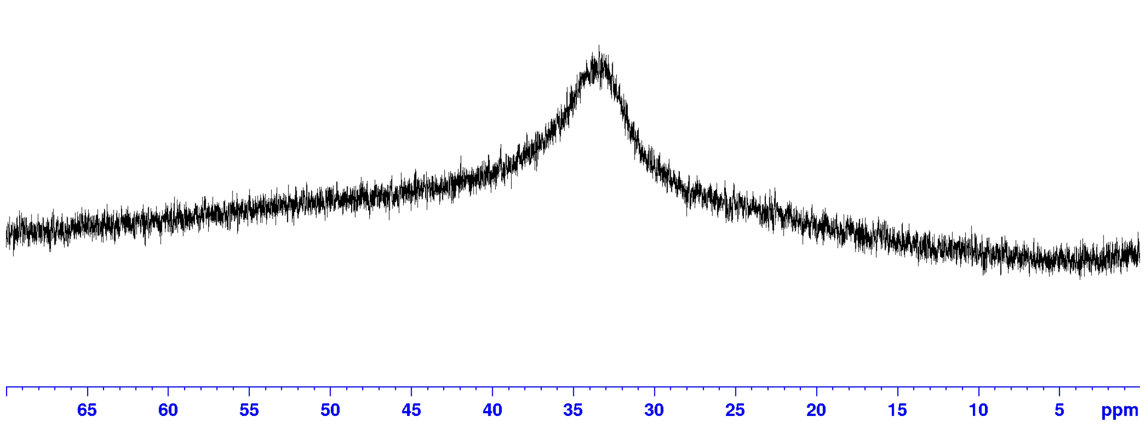

[^{1}H, $^{13}C\{^{1}H\}$, and ^{11}B NMR Spectra of **3o-Bdan**]

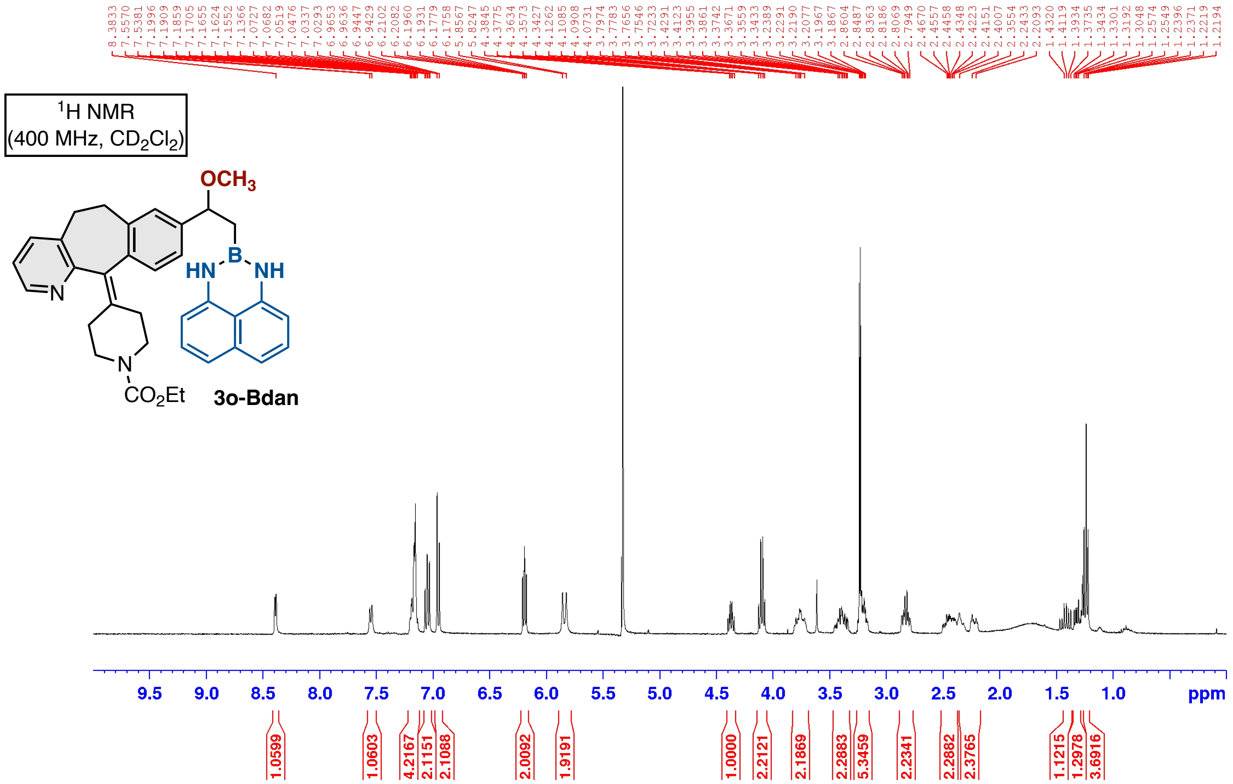

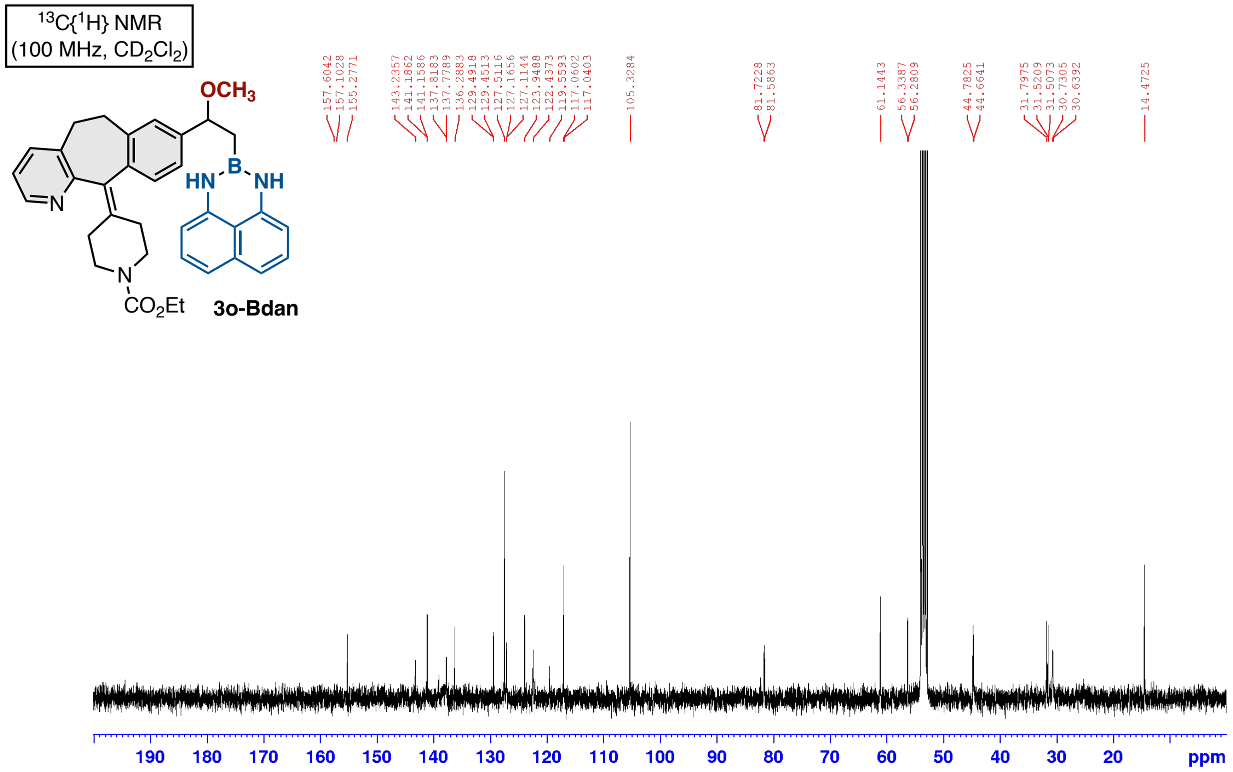

^{11}B NMR
(128 MHz, CD_2Cl_2)

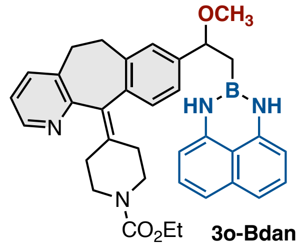

3o-Bdan

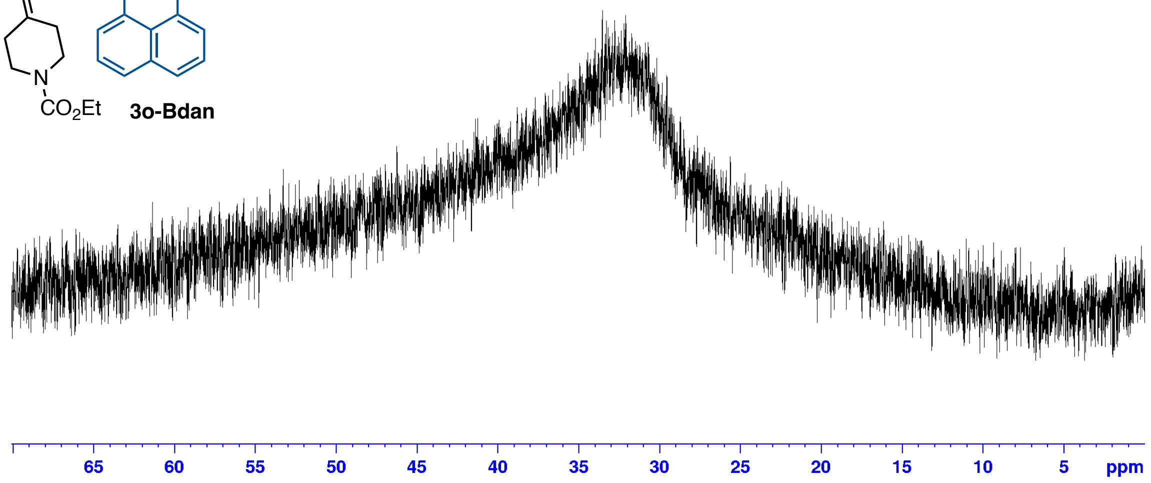

[^{1}H, ^{13}C{^{1}H}, and ^{11}B NMR Spectra of **3p-Bdan**]

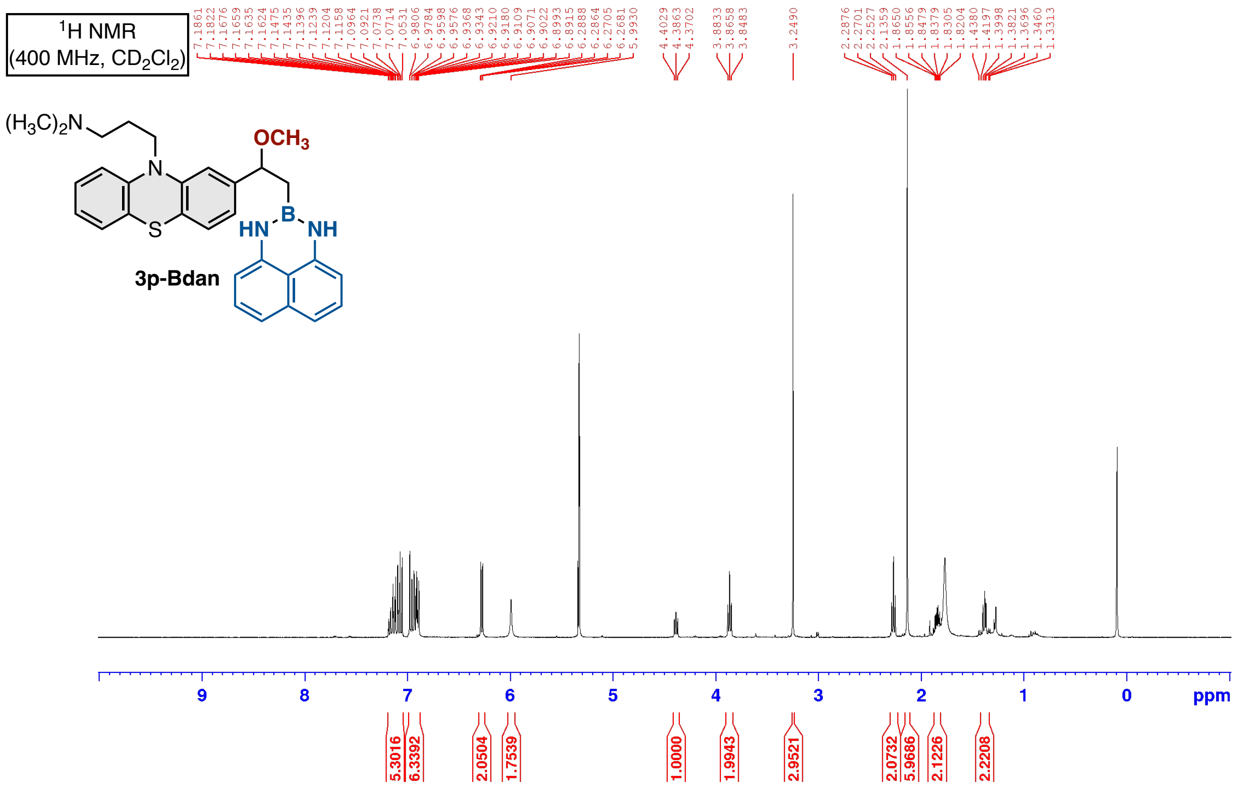

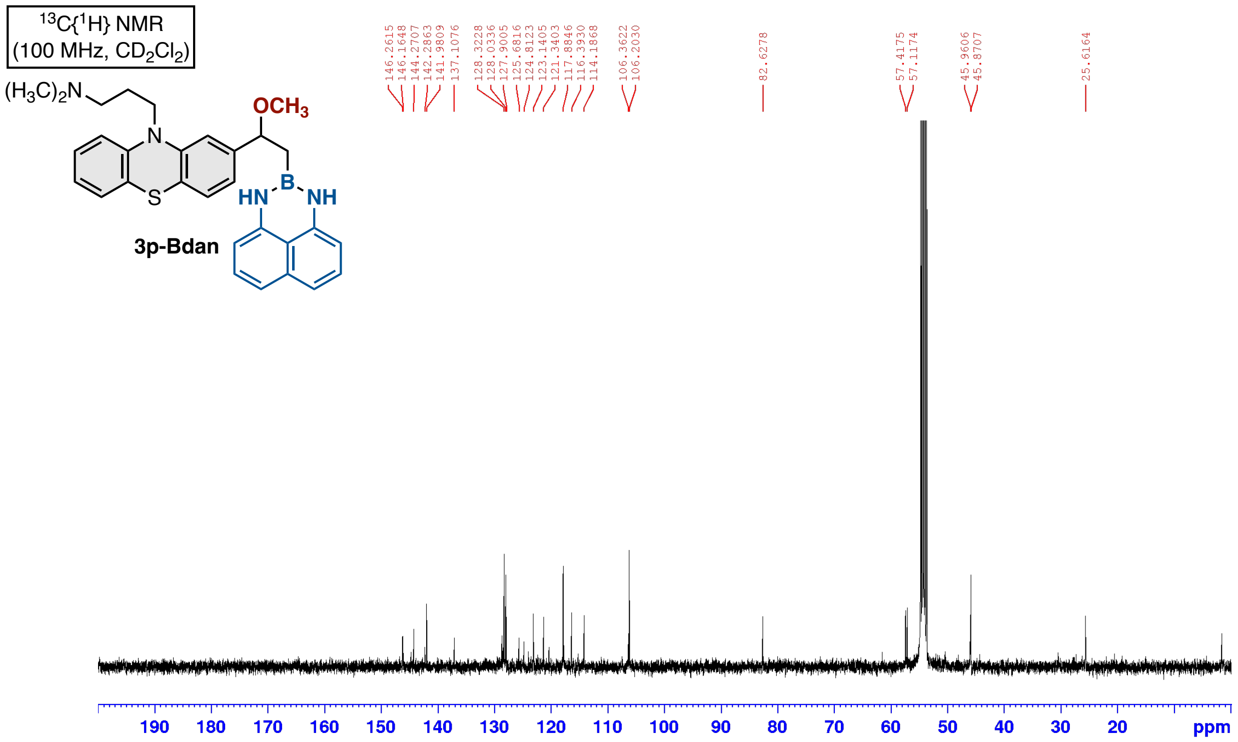

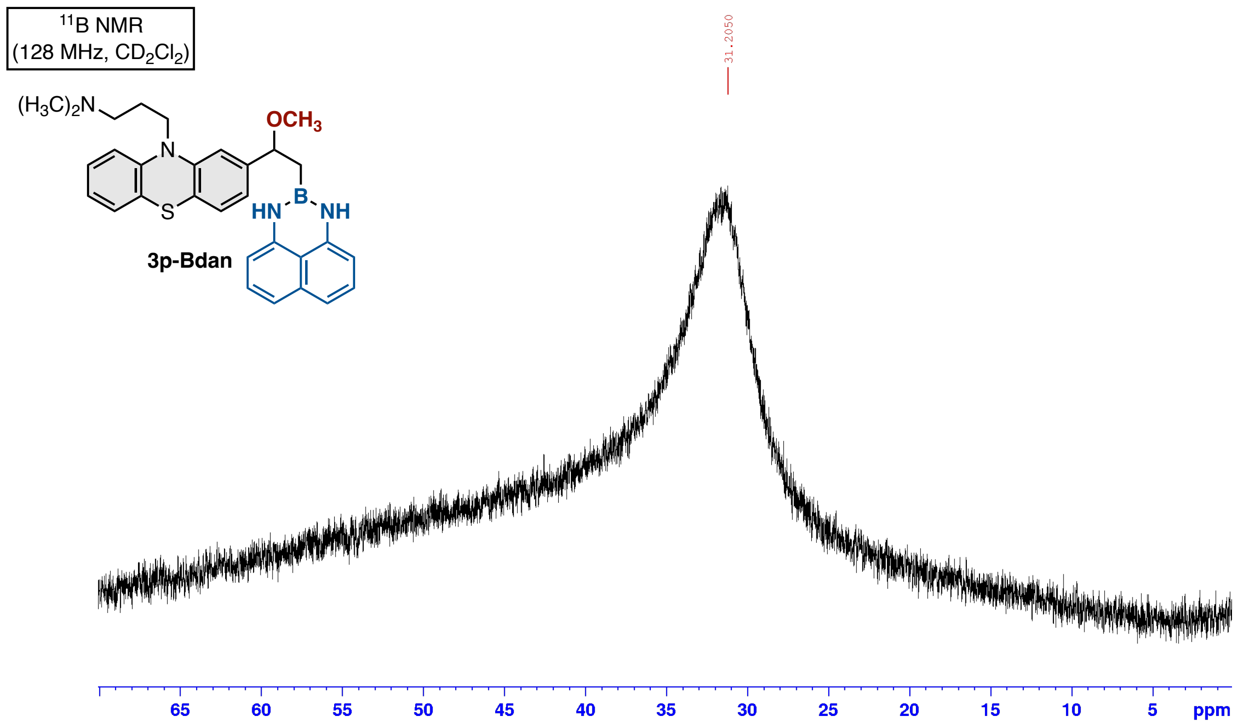

11B NMR
(128 MHz, CD2Cl2)
(H3C)2N
N
S
OCH3
B
HN
NH
3p-Bdan
31.2050
65
60
55
50
45
40
35
30
25
20
15
10
5
ppm

[^{1}H, ^{13}C{^{1}H}, and ^{11}B NMR Spectra of **3q**]

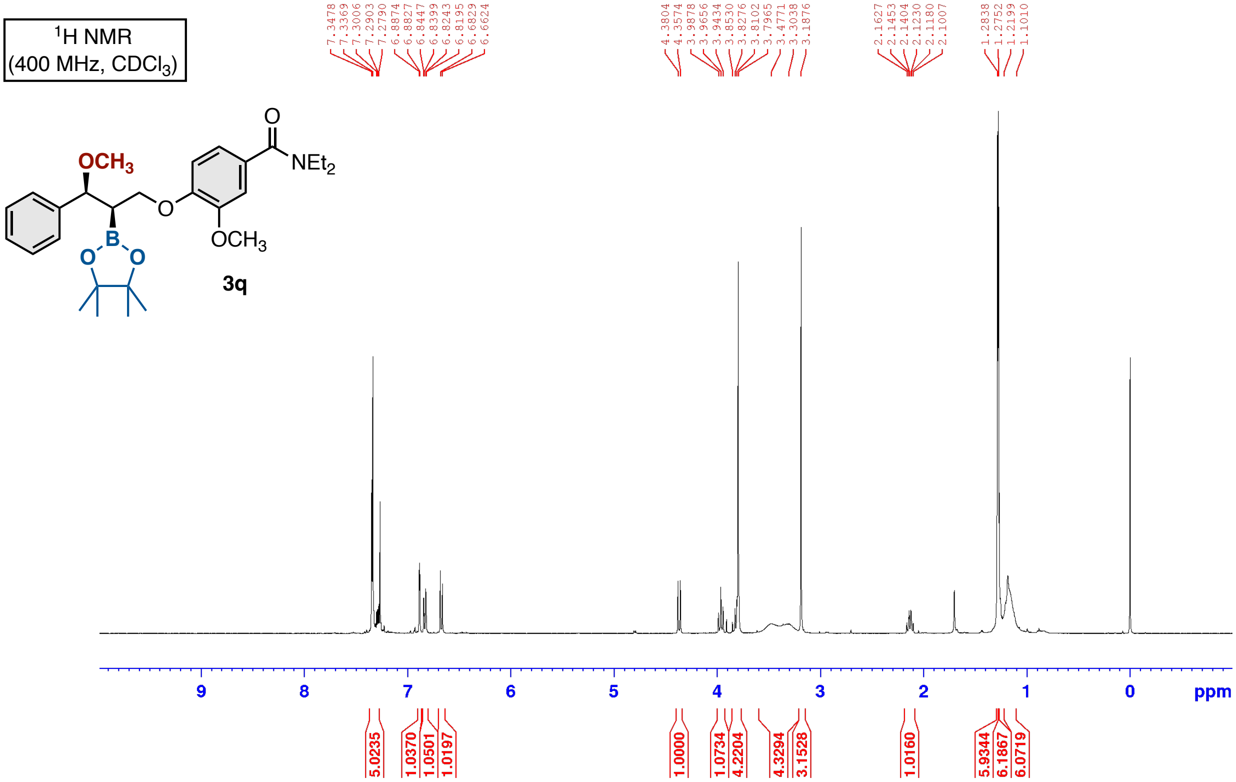

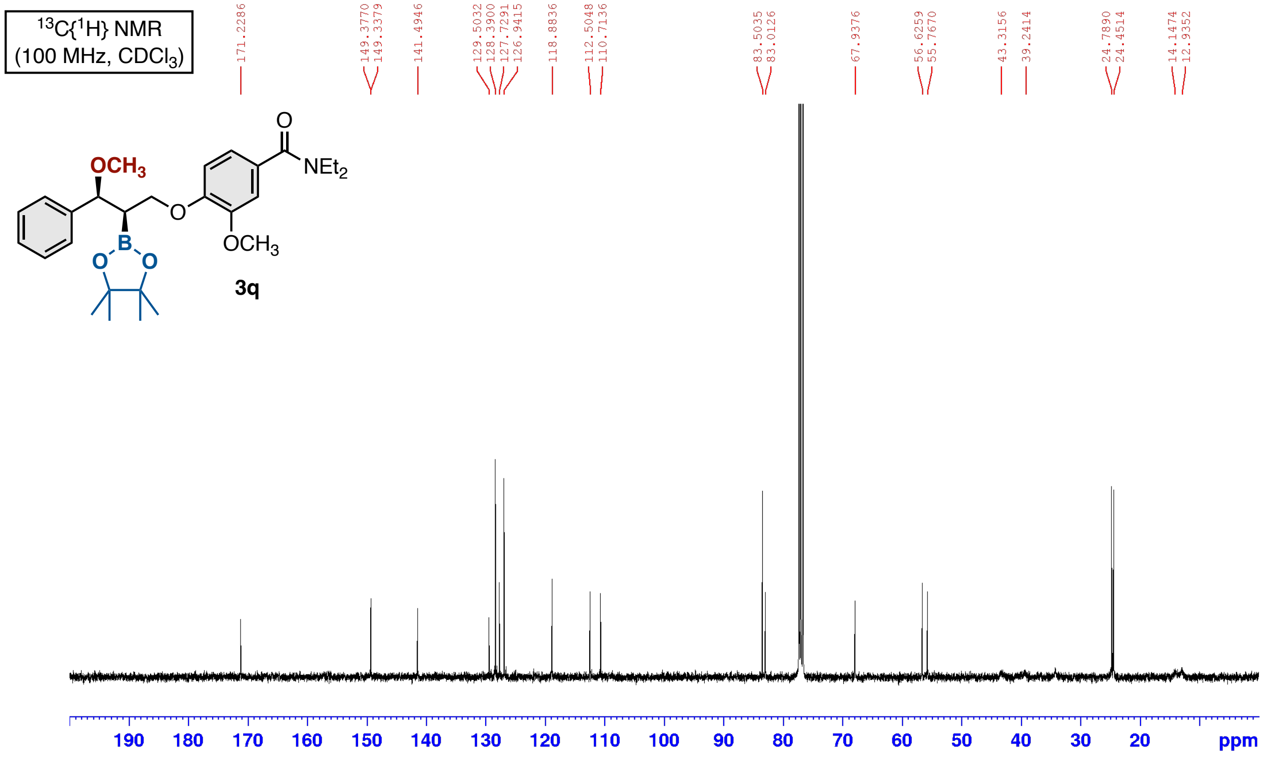

^{11}B NMR
(128 MHz, $CDCl_3$)

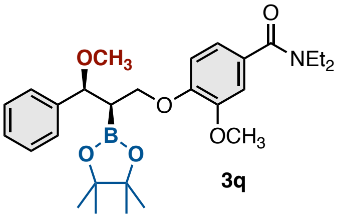

3q

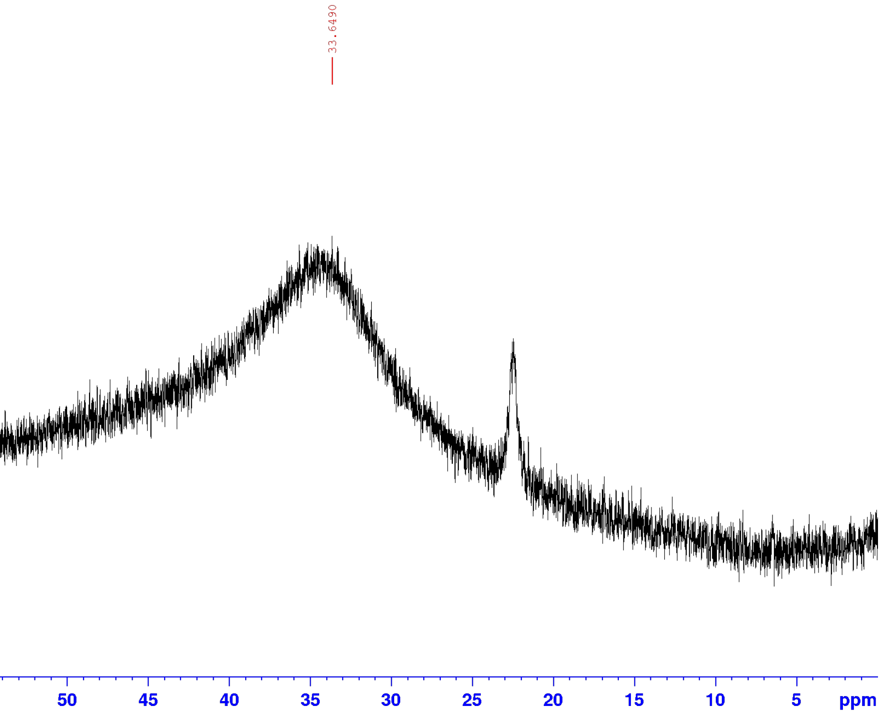

[^{1}H, ^{13}C{^{1}H}, and ^{11}B NMR Spectra of *anti*-**3a-Bneo**]

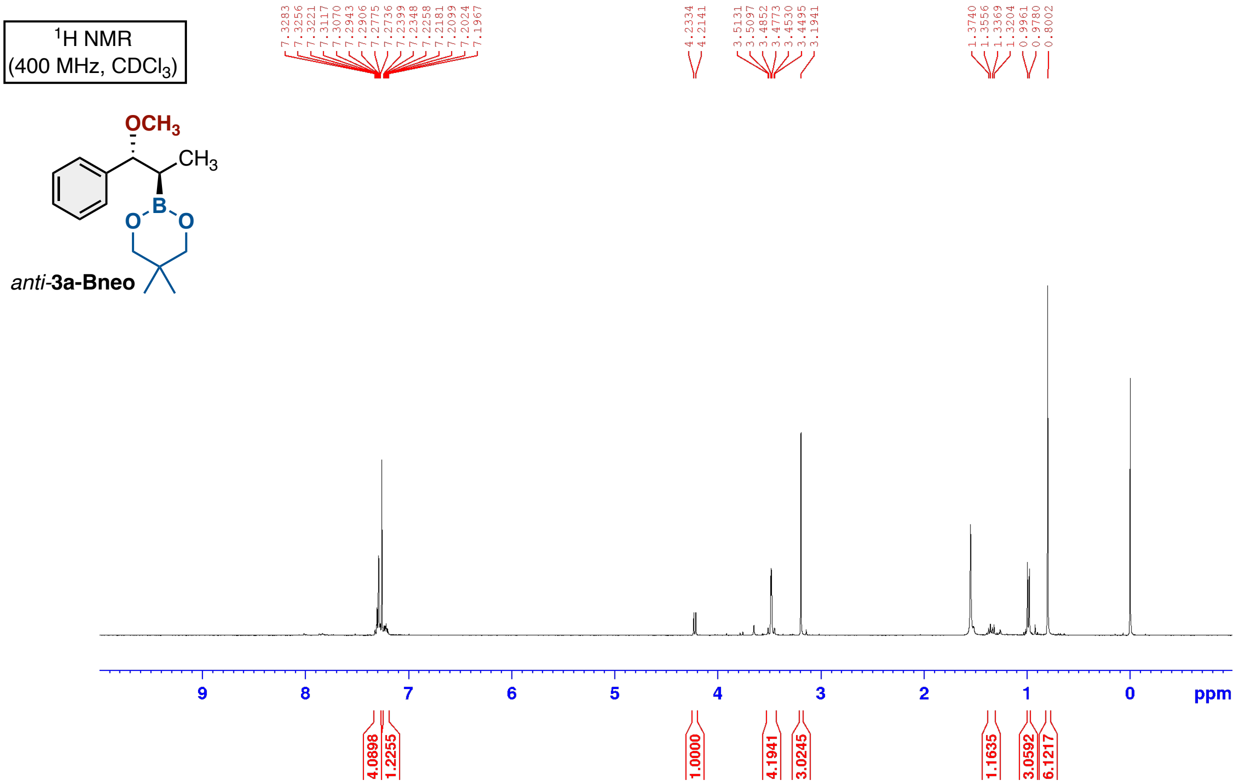

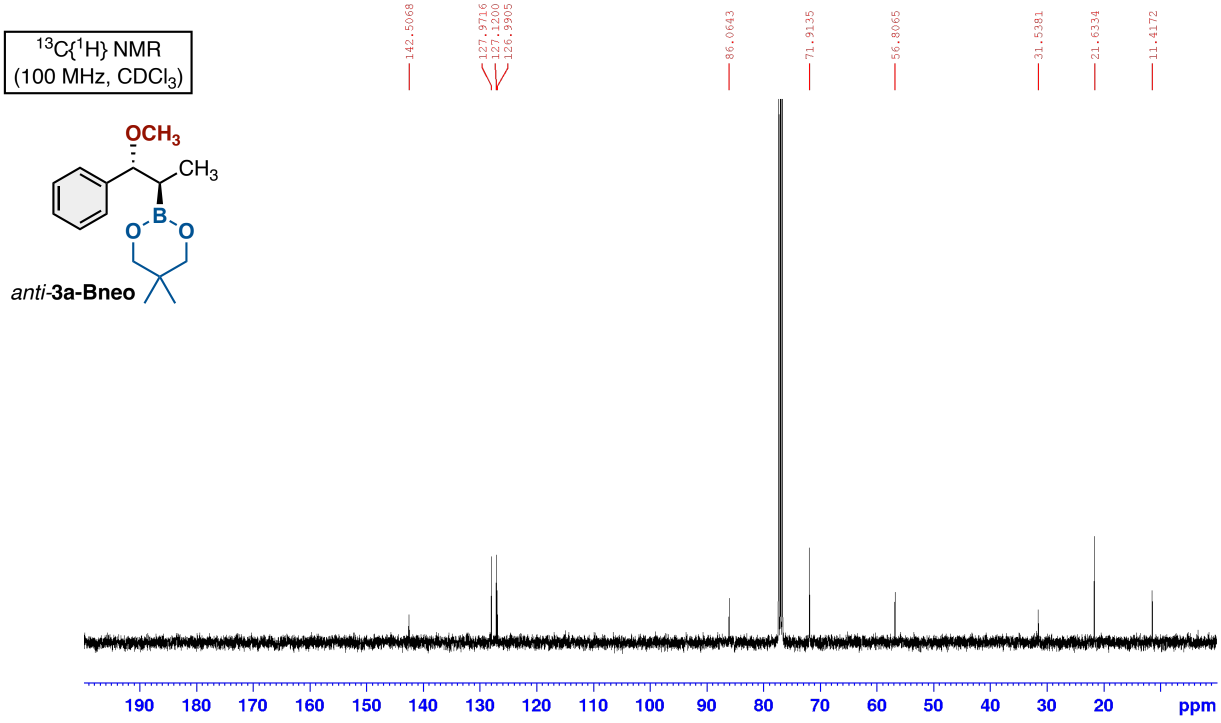

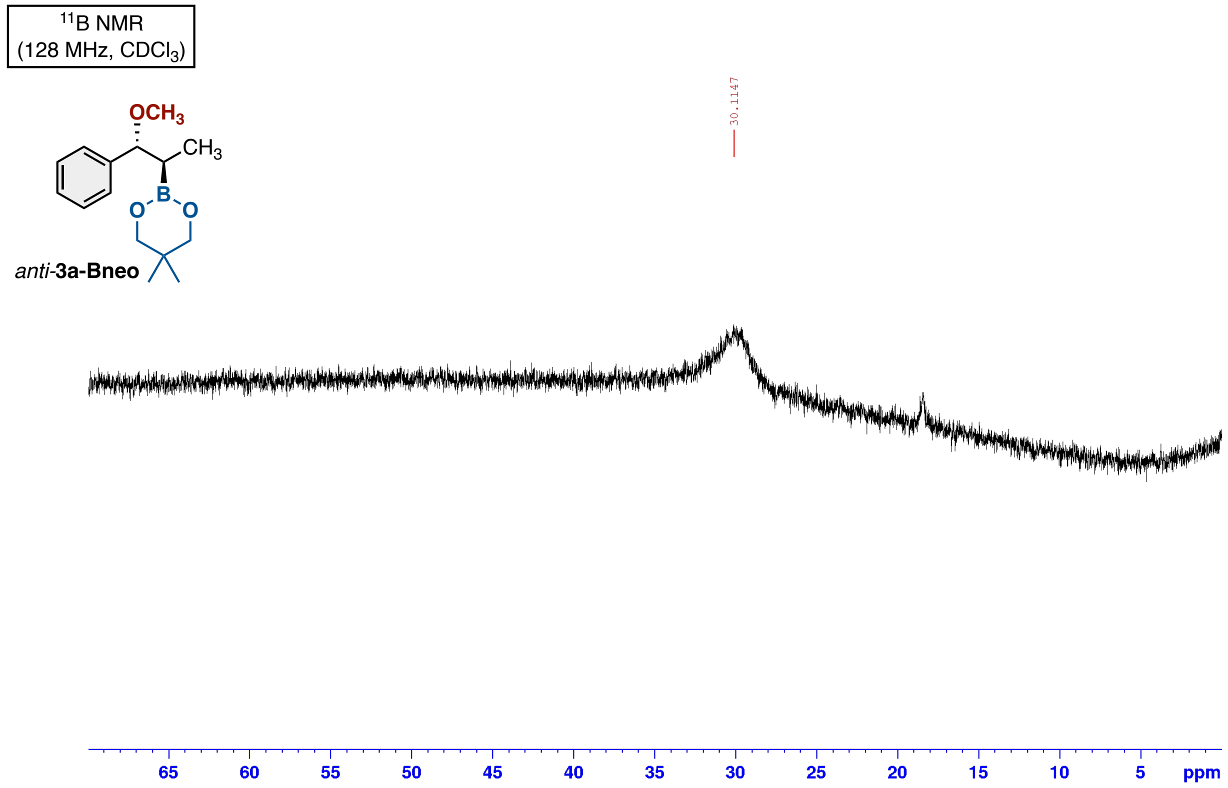

11B NMR
(128 MHz, CDCl3)
OCH3
CH3
B
O
O
anti-3a-Bneo
30.1147
65
60
55
50
45
40
35
30
25
20
15
10
5
ppm

[^{1}H, ^{13}C{^{1}H}, and ^{11}B NMR Spectra of **3r**]

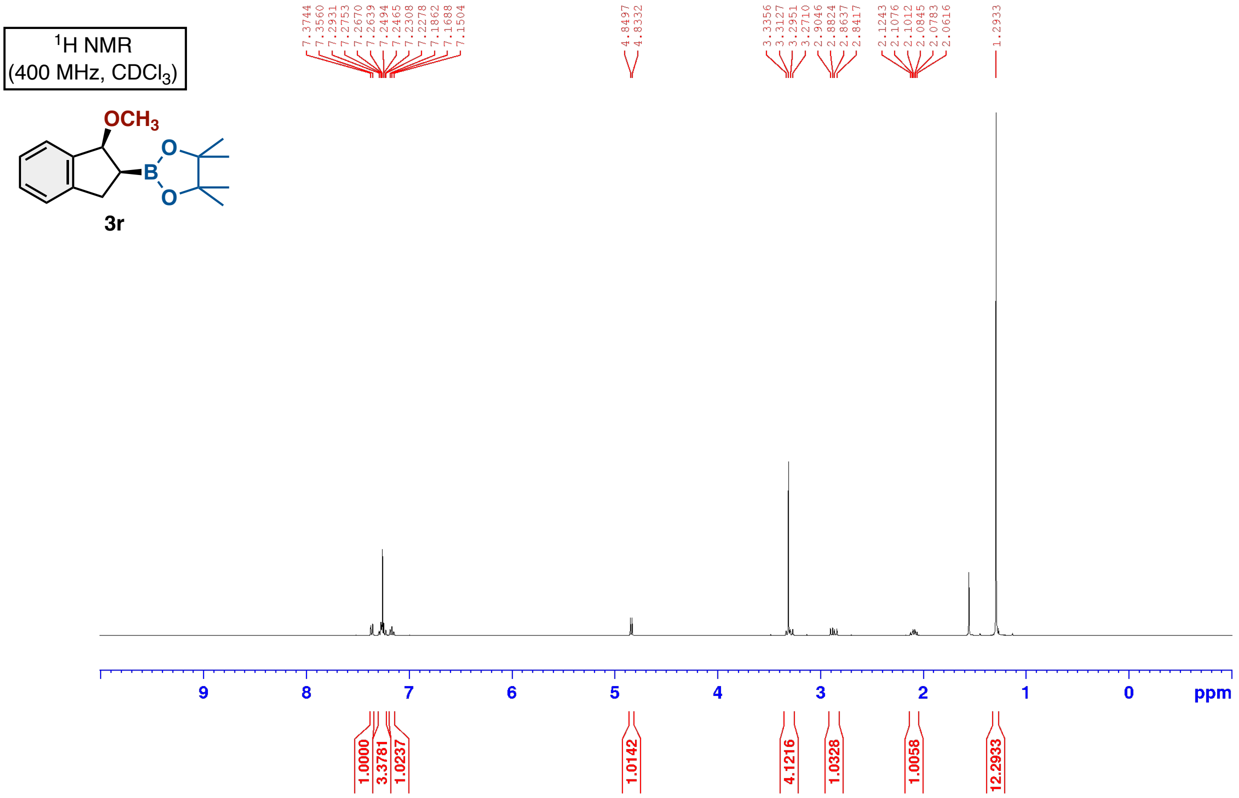

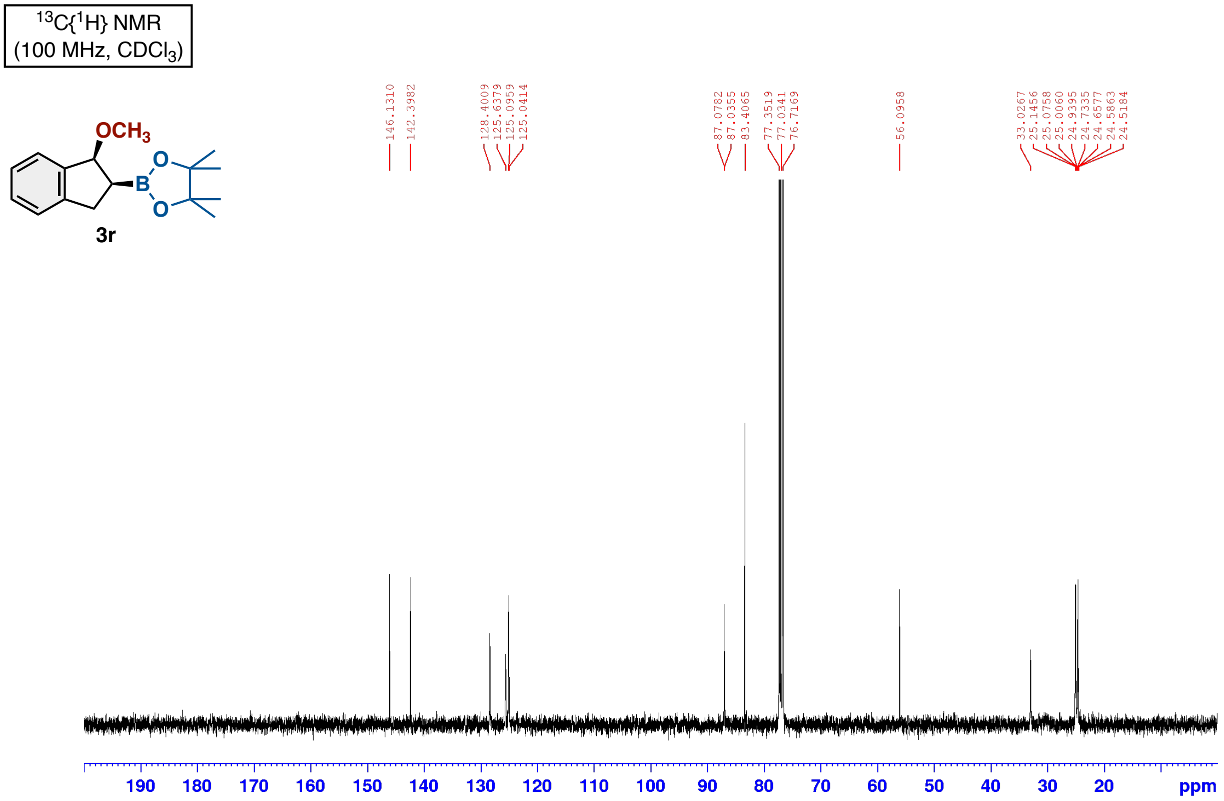

^{11}B NMR
(128 MHz, $CDCl_3$)

33.1561

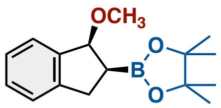

3r

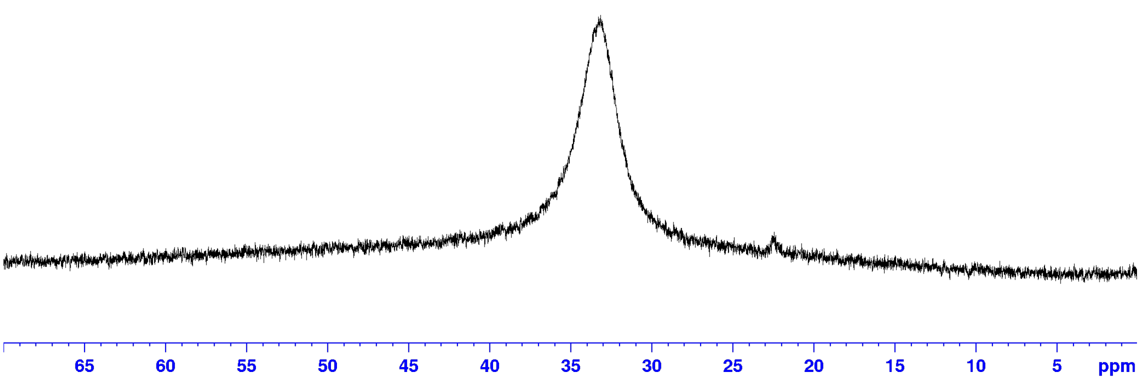

[^{1}H, ^{13}C{^{1}H}, and ^{11}B NMR Spectra of **3s**]

^{1}H NMR
(400 MHz, $CDCl_3$)

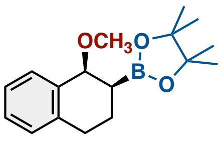

3s

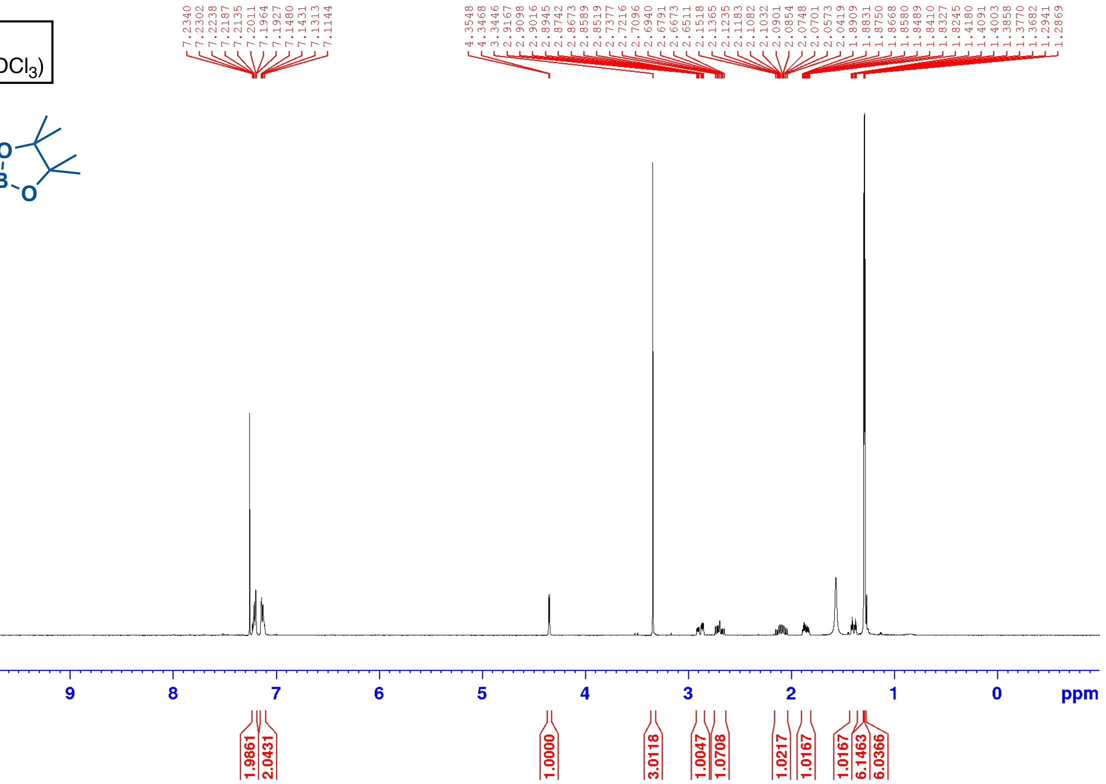

^{13}C{^{1}H} NMR
(100 MHz, $CDCl_3$)

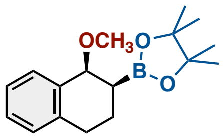

3s

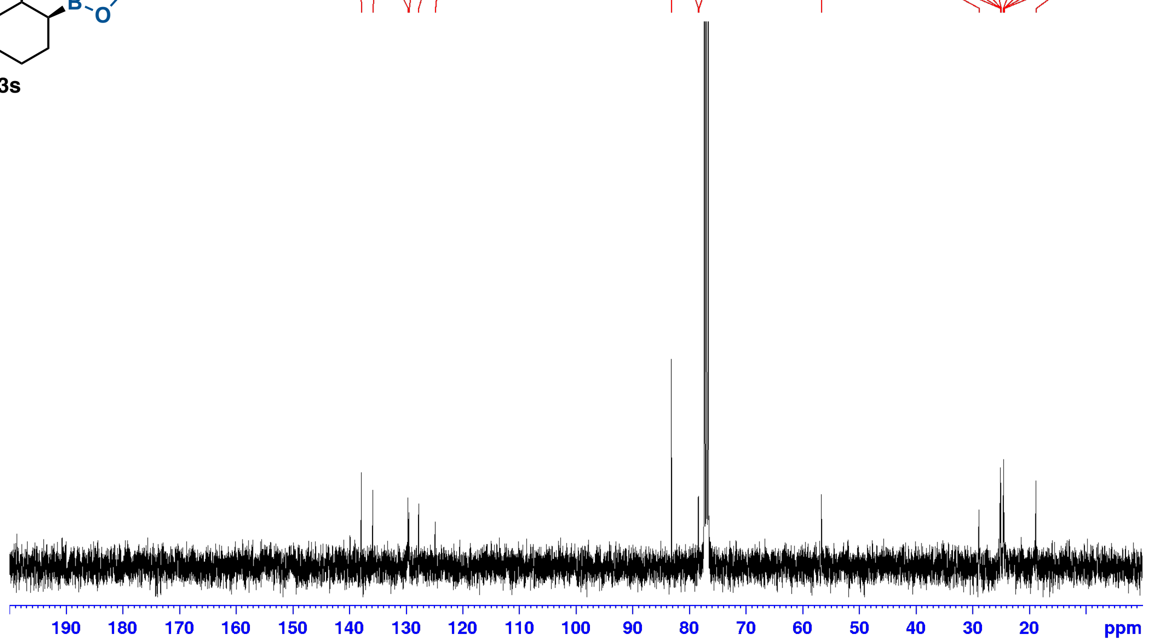

^{11}B NMR
(128 MHz, $CDCl_3$)

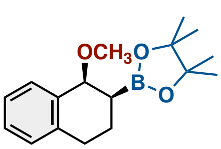

3s

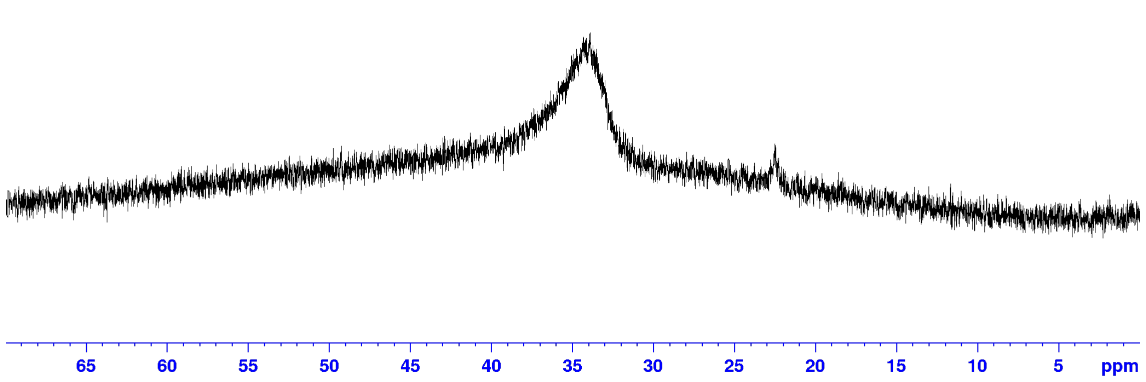

[^{1}H, $^{13}C\{^{1}H\}$, and ^{11}B NMR Spectra of **3t**]

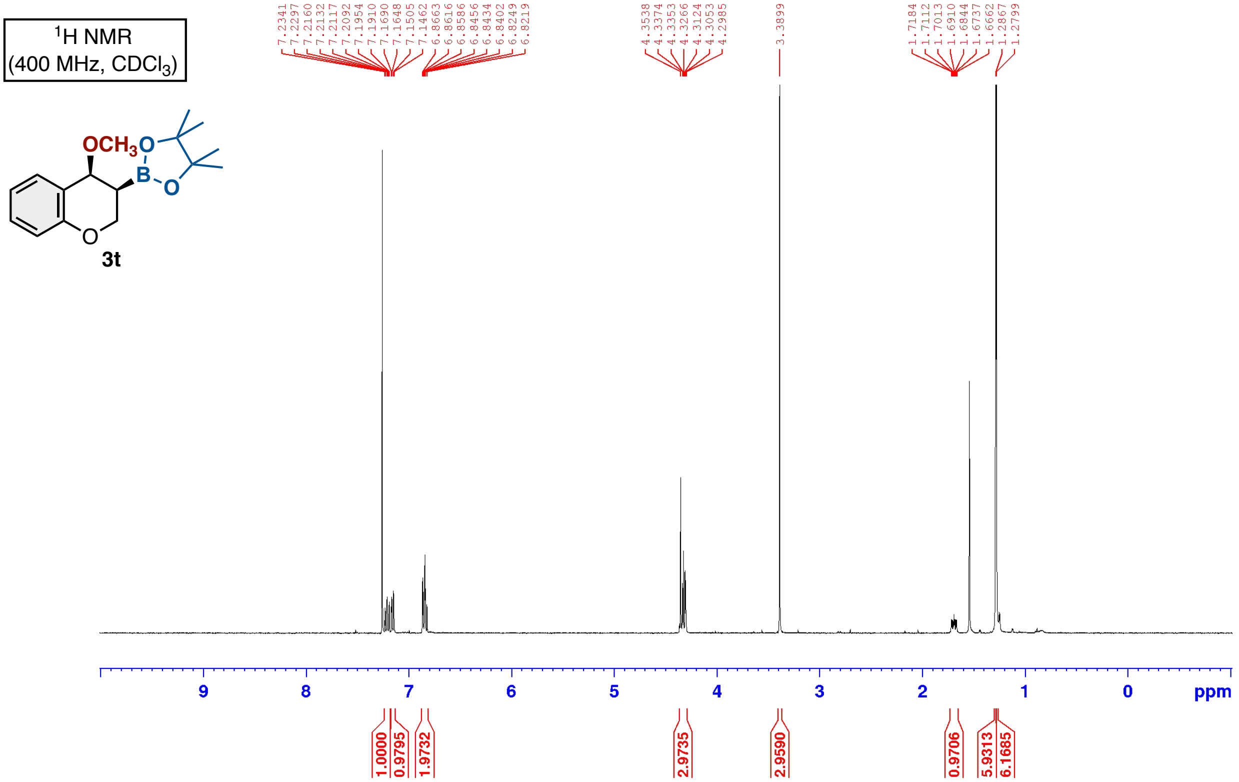

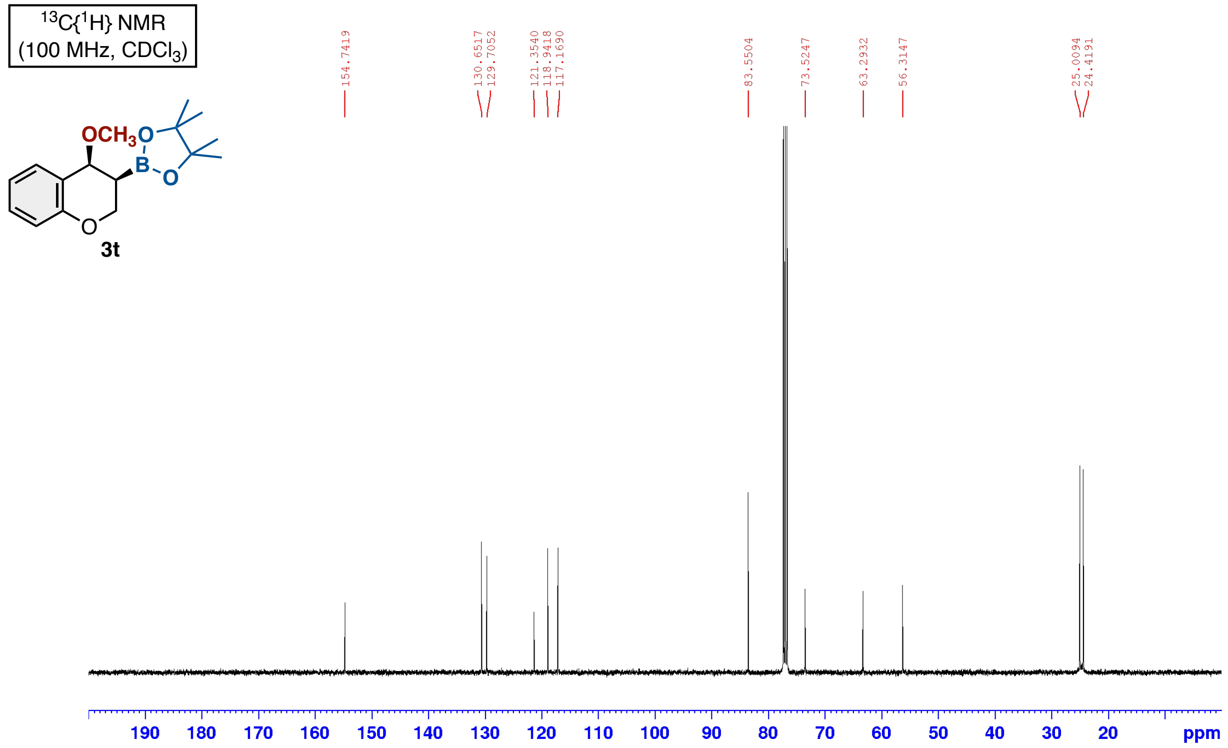

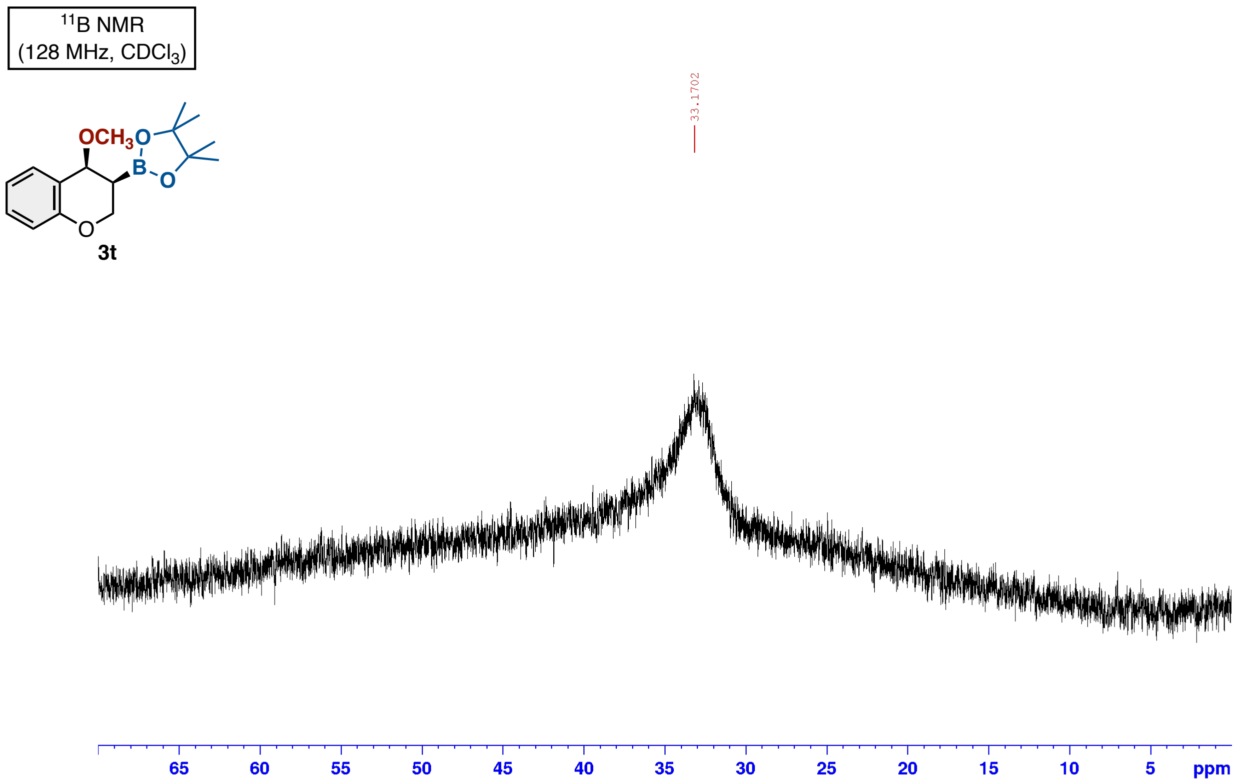

^{11}B NMR
(128 MHz, $CDCl_3$)
OCH3
O
B
O
O
3t
33.1702
65
60
55
50
45
40
35
30
25
20
15
10
5
ppm

[^{1}H, ^{13}C{^{1}H}, and ^{11}B NMR Spectra of **3u**]

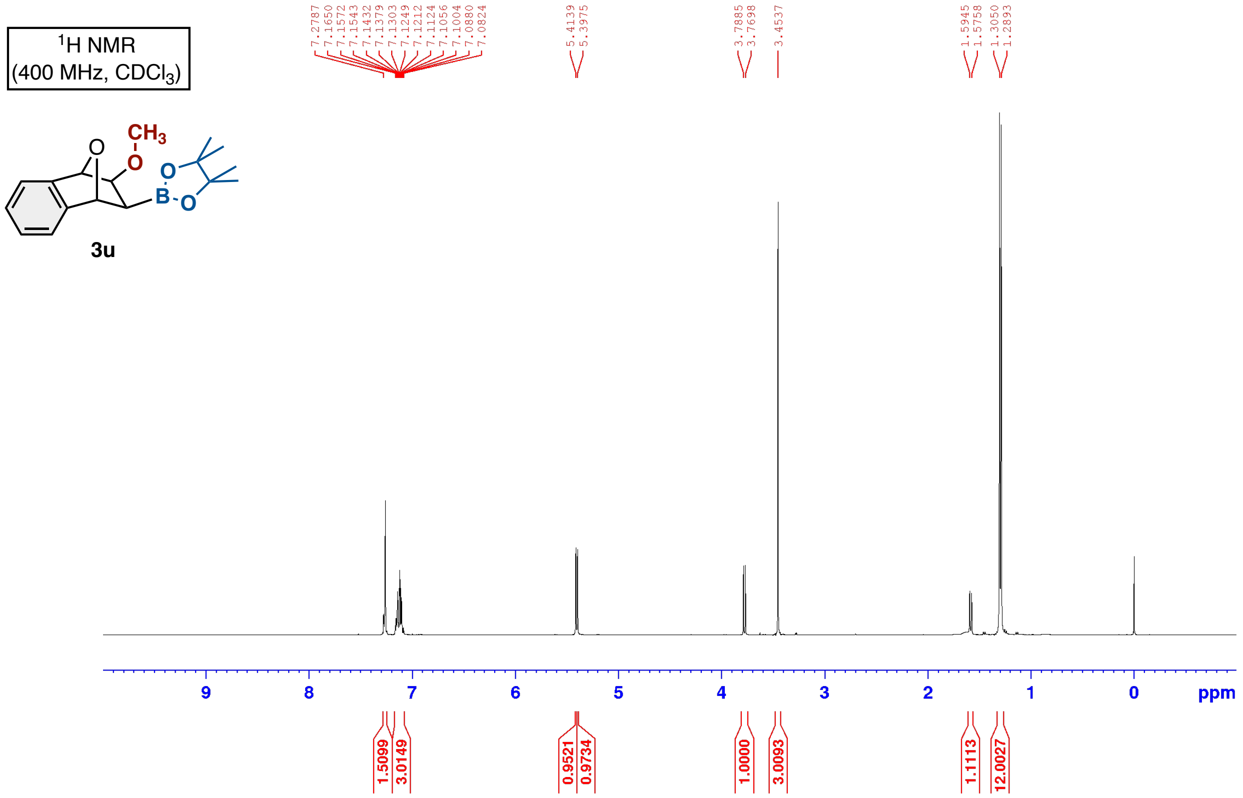

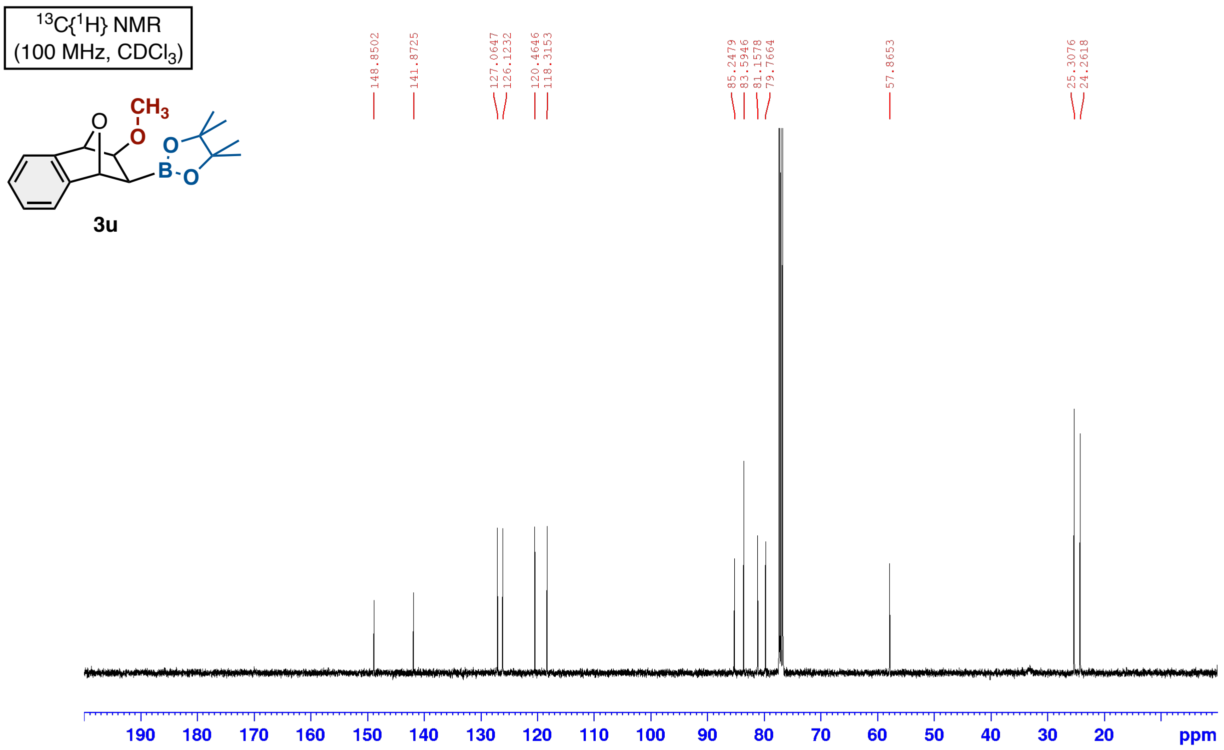

^{11}B NMR
(128 MHz, $CDCl_3$)

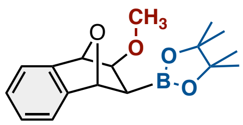

3u

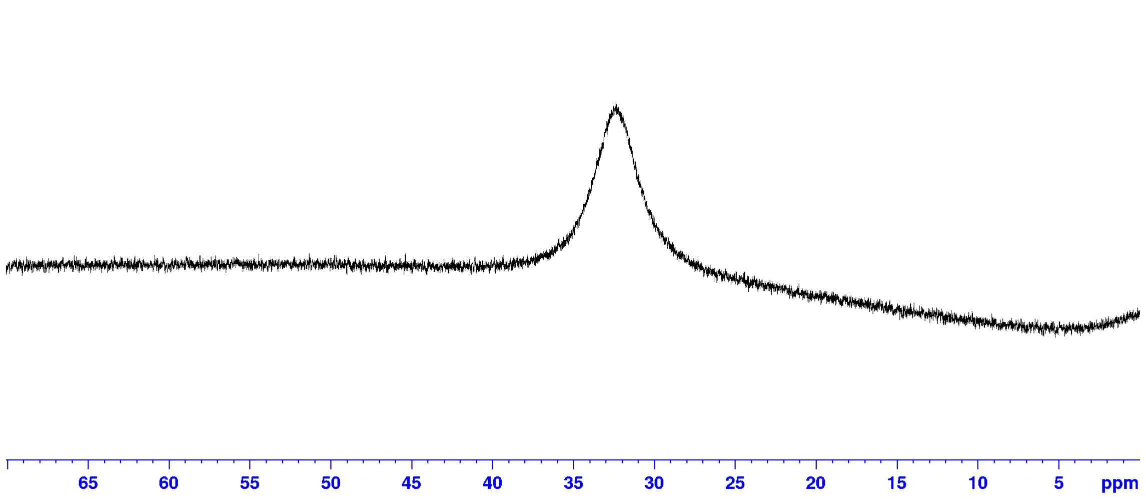

[^{1}H and ^{13}C{^{1}H} NMR Spectra of *syn*-**4**]

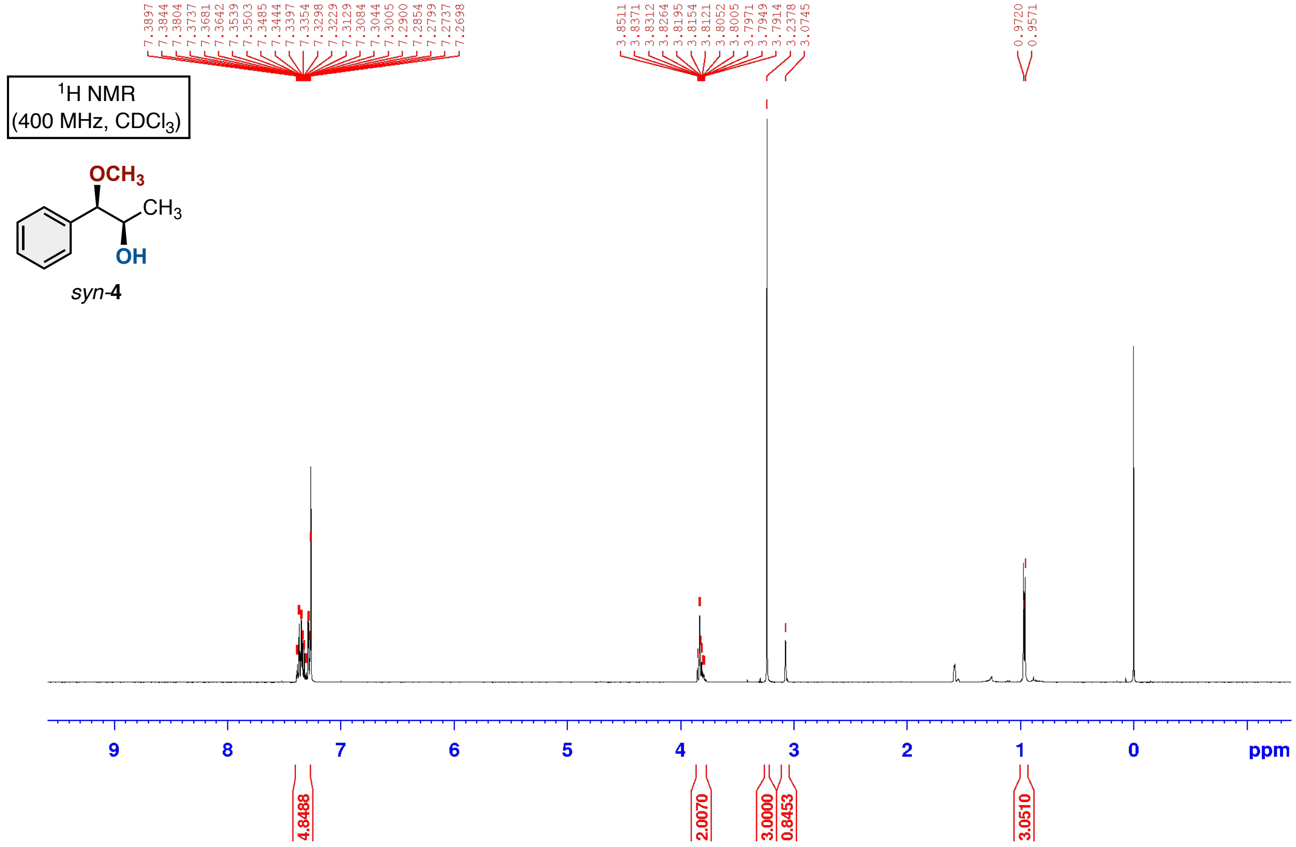

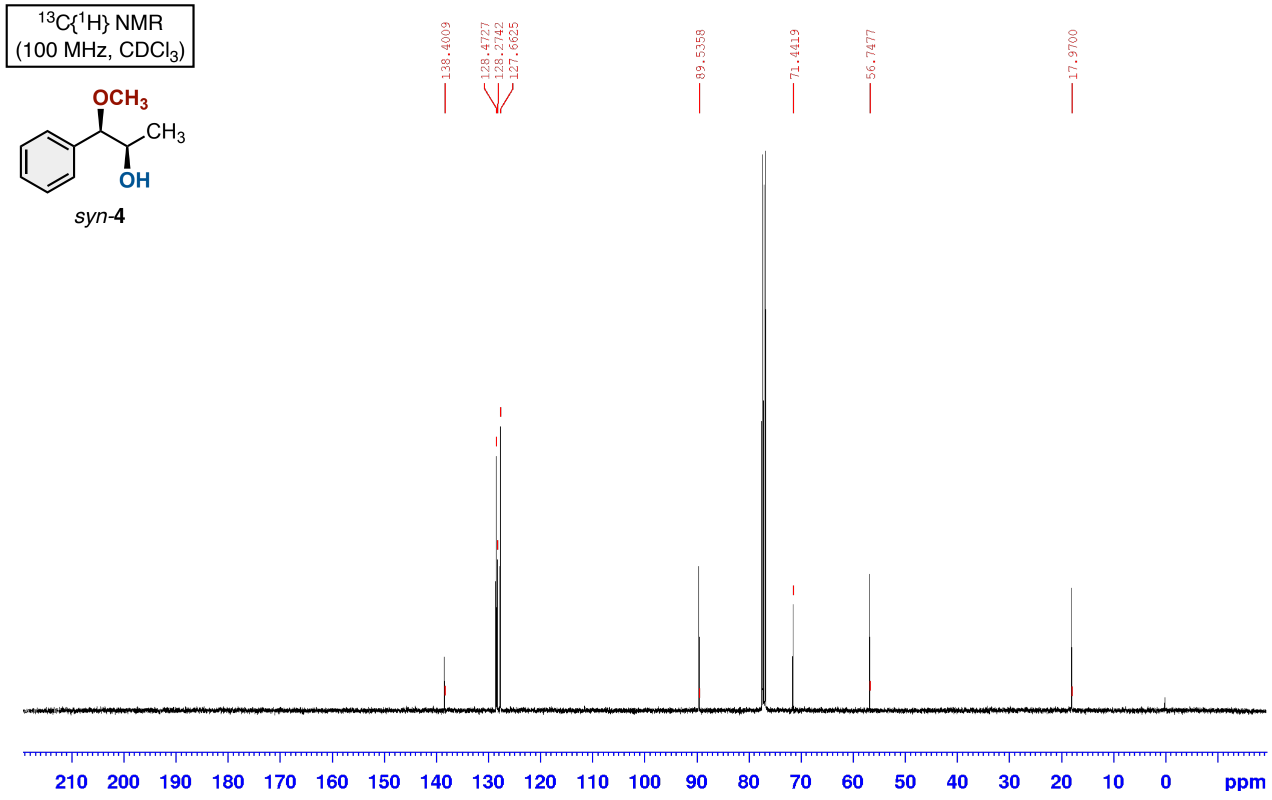

[^{1}H and ^{13}C{^{1}H} NMR Spectra of *anti*-**4**]

^{1}H NMR
(400 MHz, $CDCl_3$)

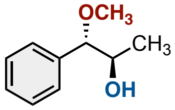

anti-**4**

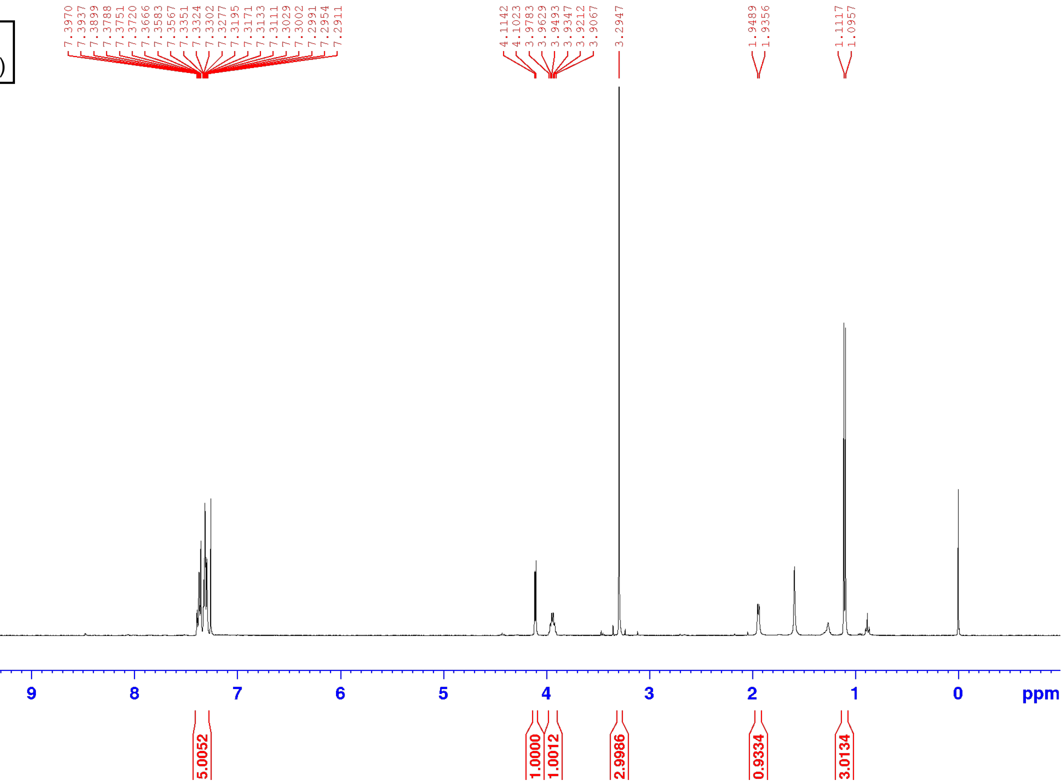

^{13}C{^{1}H} NMR
(100 MHz, $CDCl_3$)

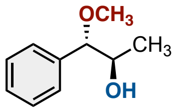

anti-**4**

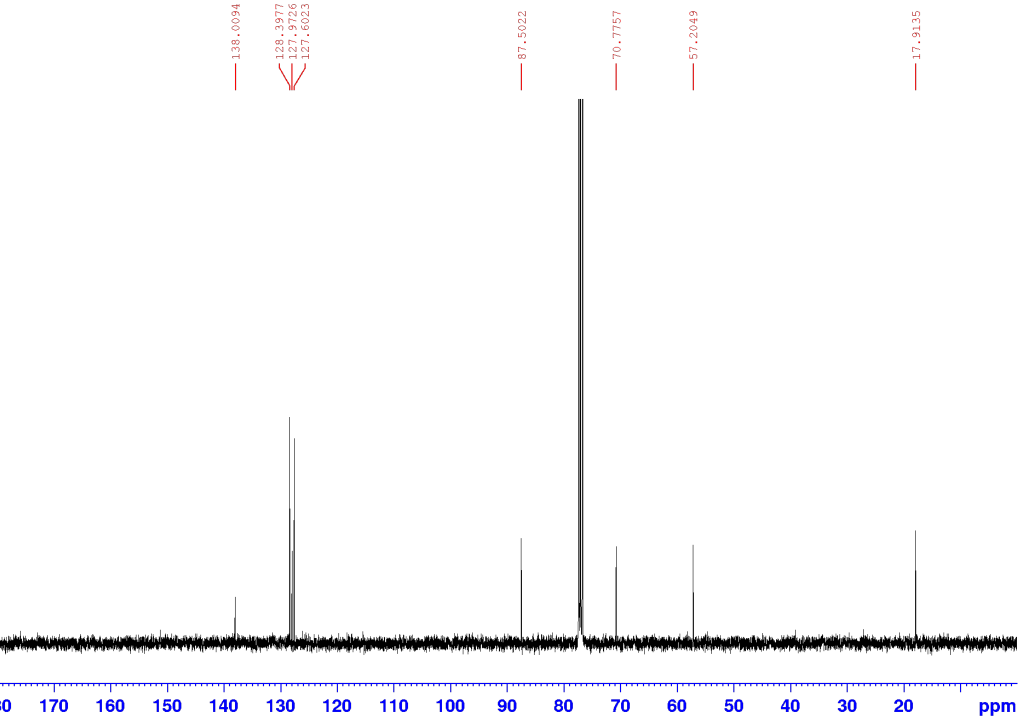

[^{1}H and ^{13}C{^{1}H} NMR Spectra of **3r-O**]

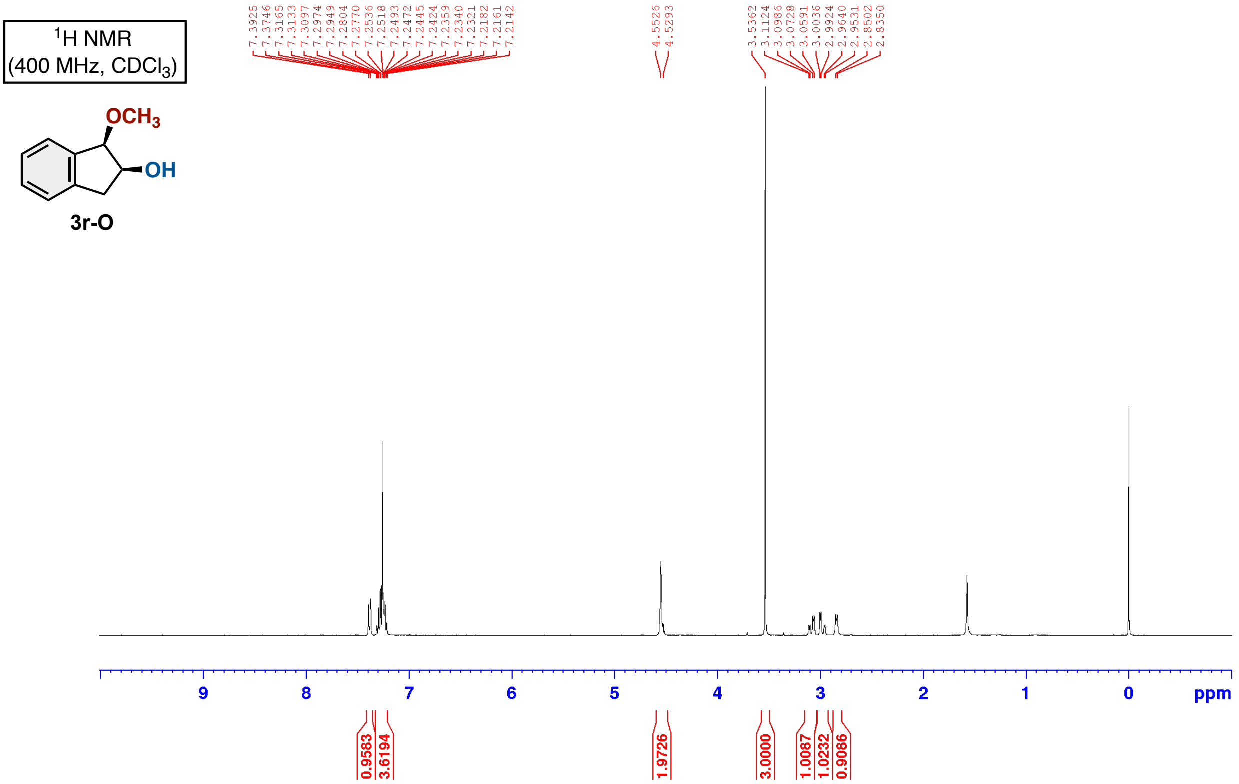

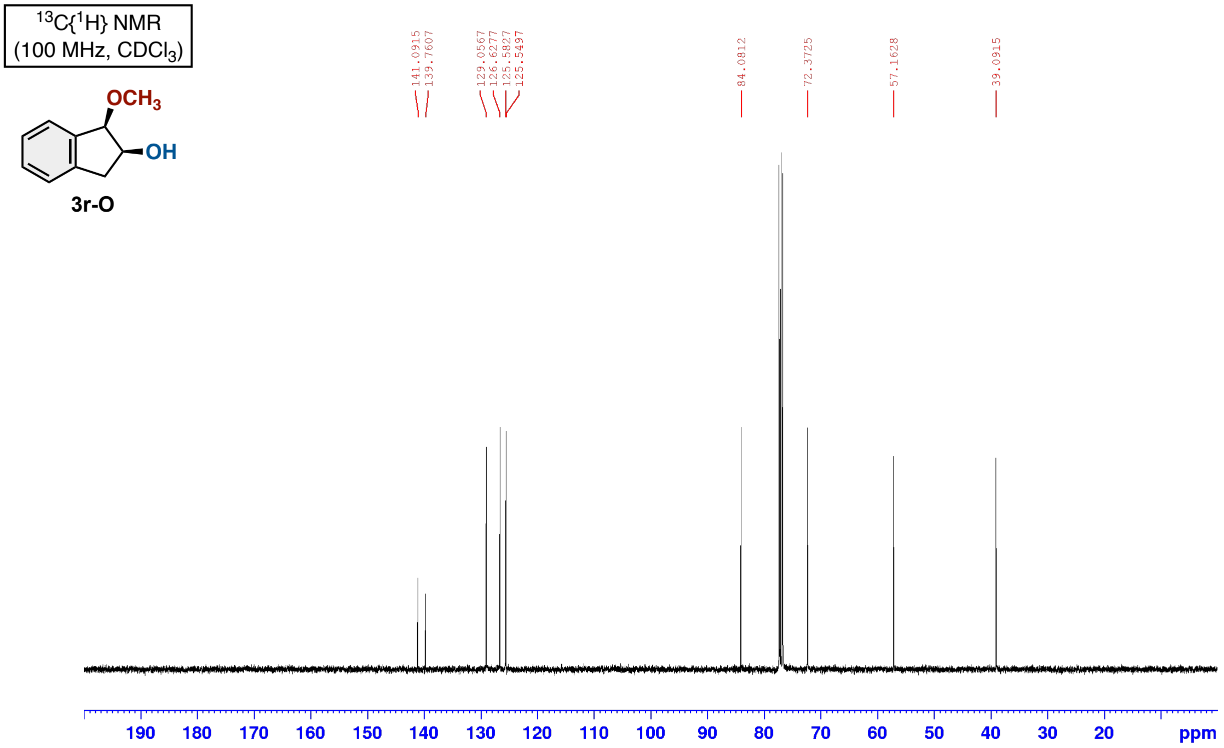

[^{1}H and ^{13}C{^{1}H}, and ^{19}F{^{1}H} NMR Spectra of **5**]

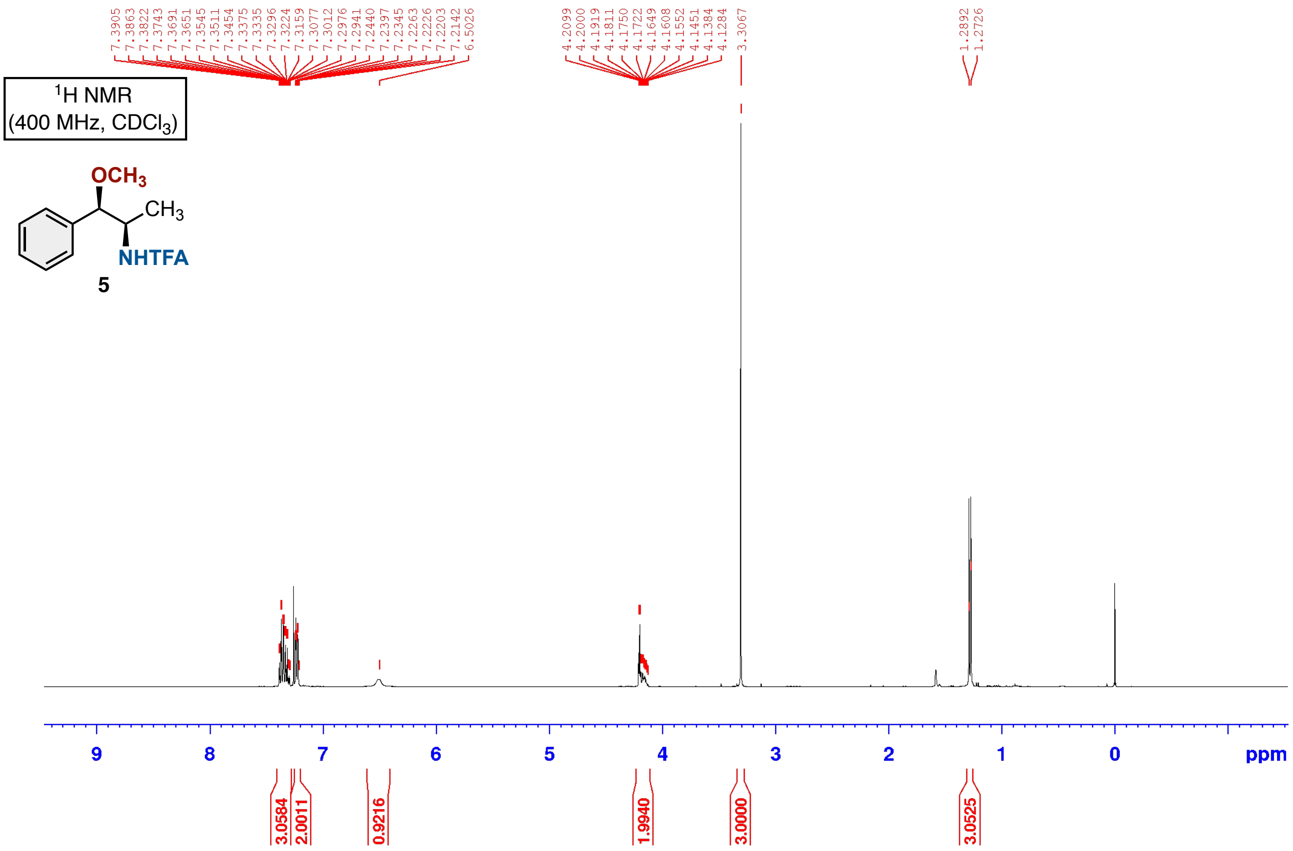

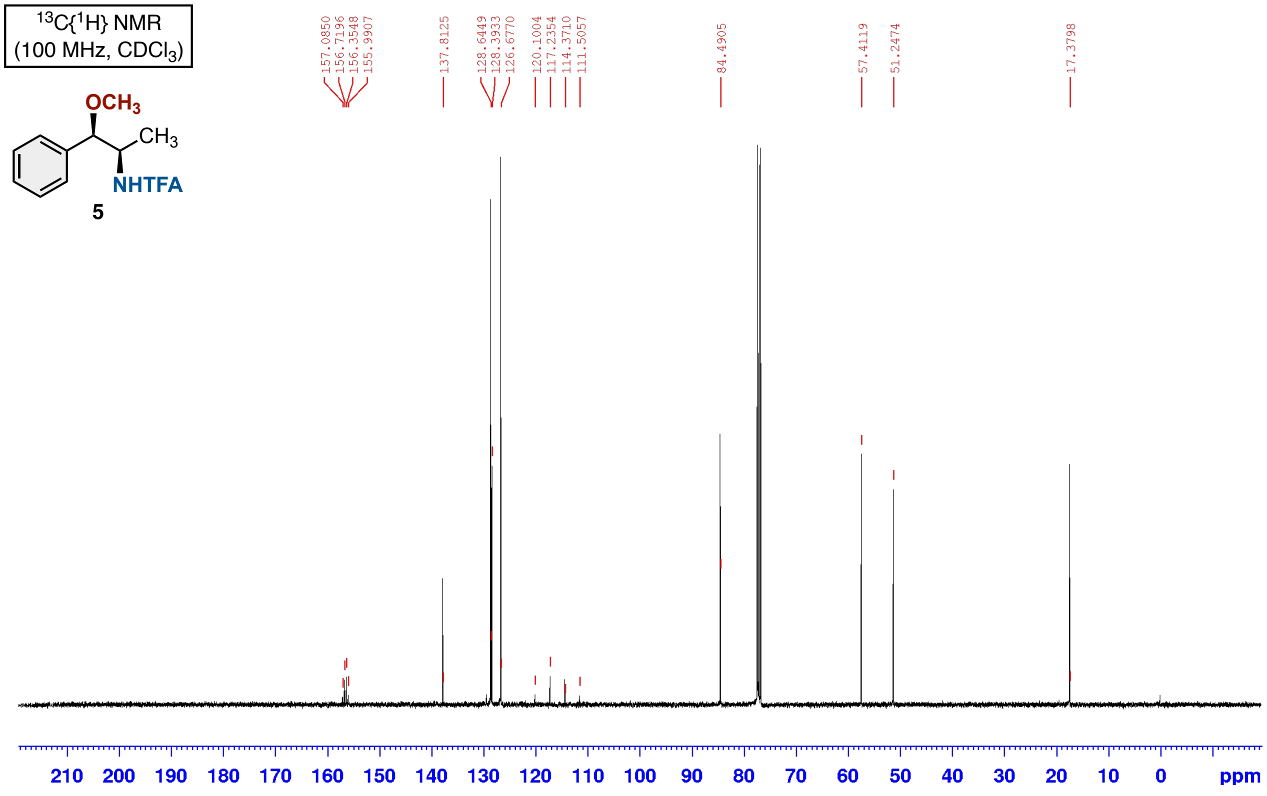

$^{19}F\{^{1}H\}$ NMR
(376 MHz, $CDCl_3$)

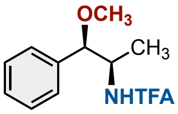

5

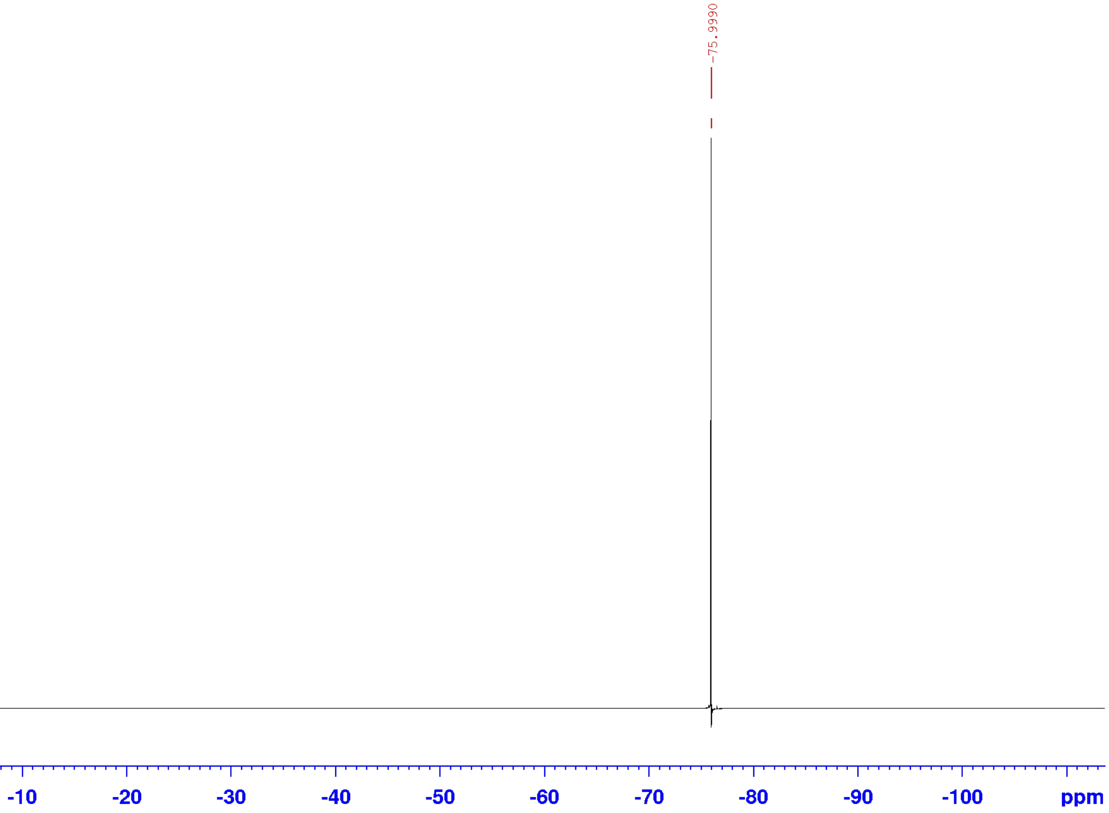

[^{1}H and ^{13}C{^{1}H} NMR Spectra of **6**]

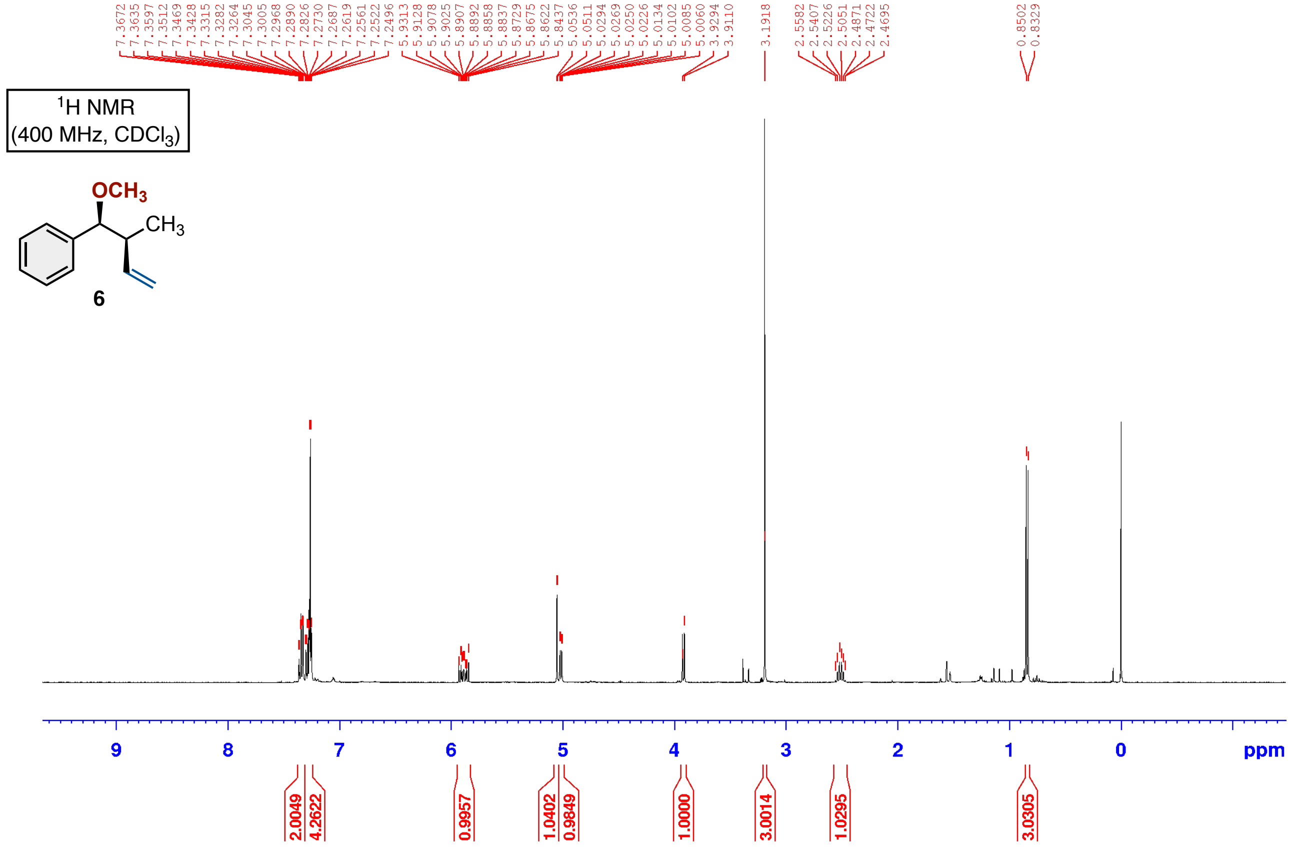

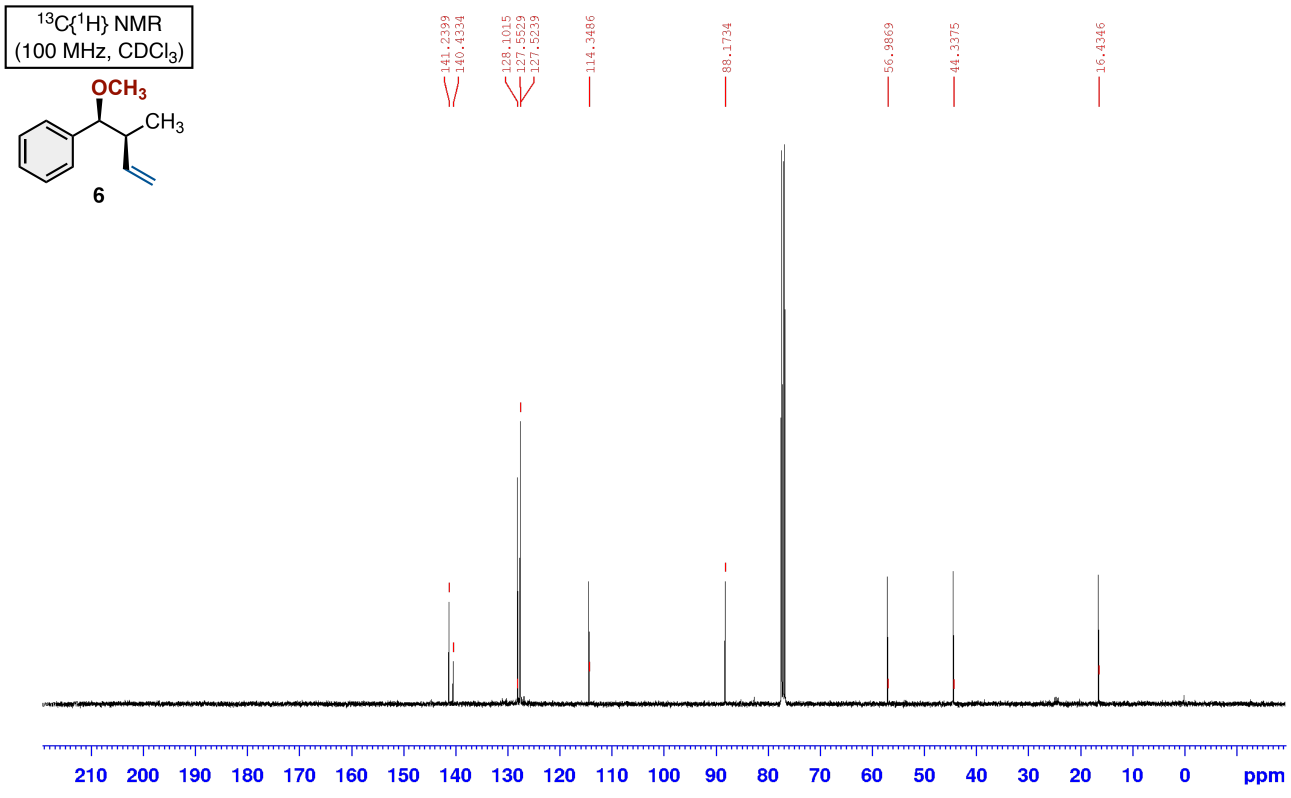

[^{1}H, ^{13}C{^{1}H}, and ^{11}B NMR Spectra of **7**]

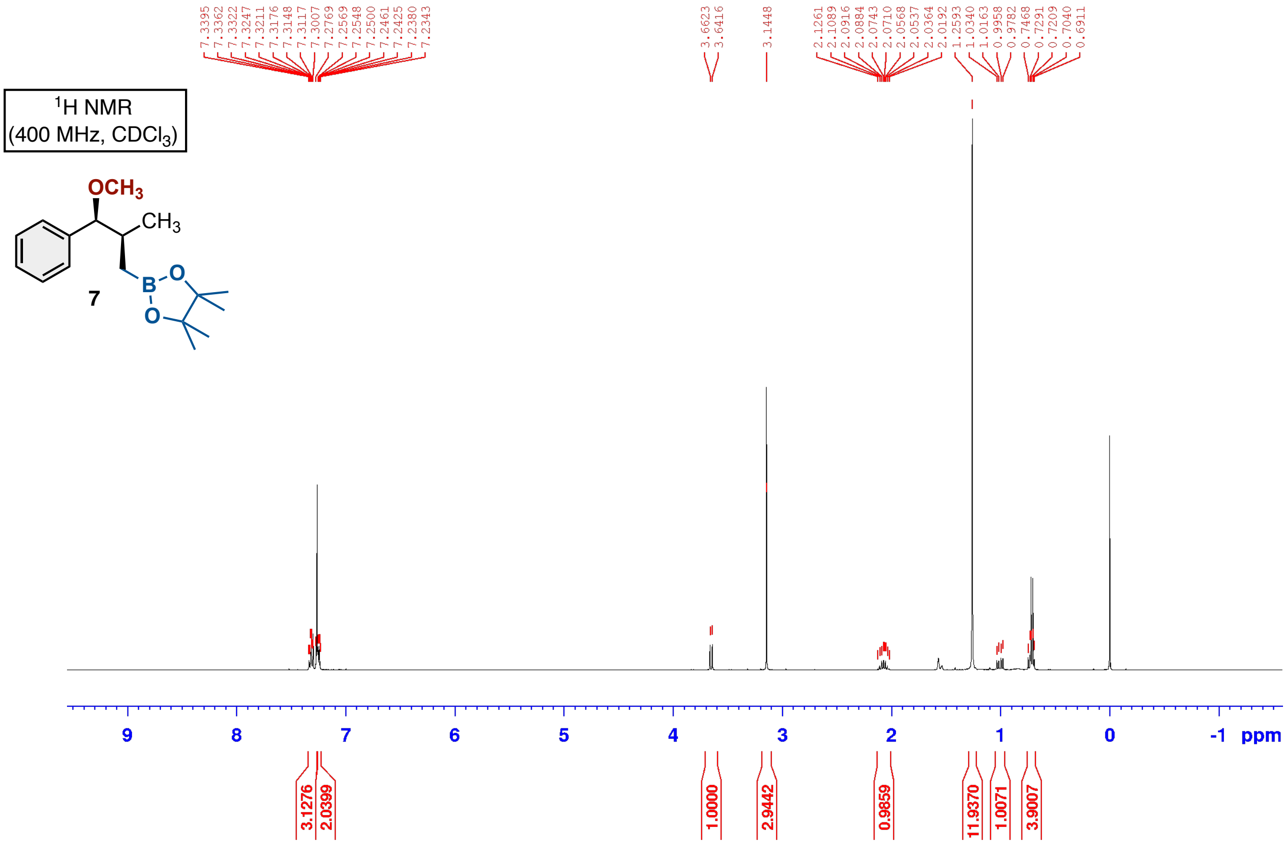

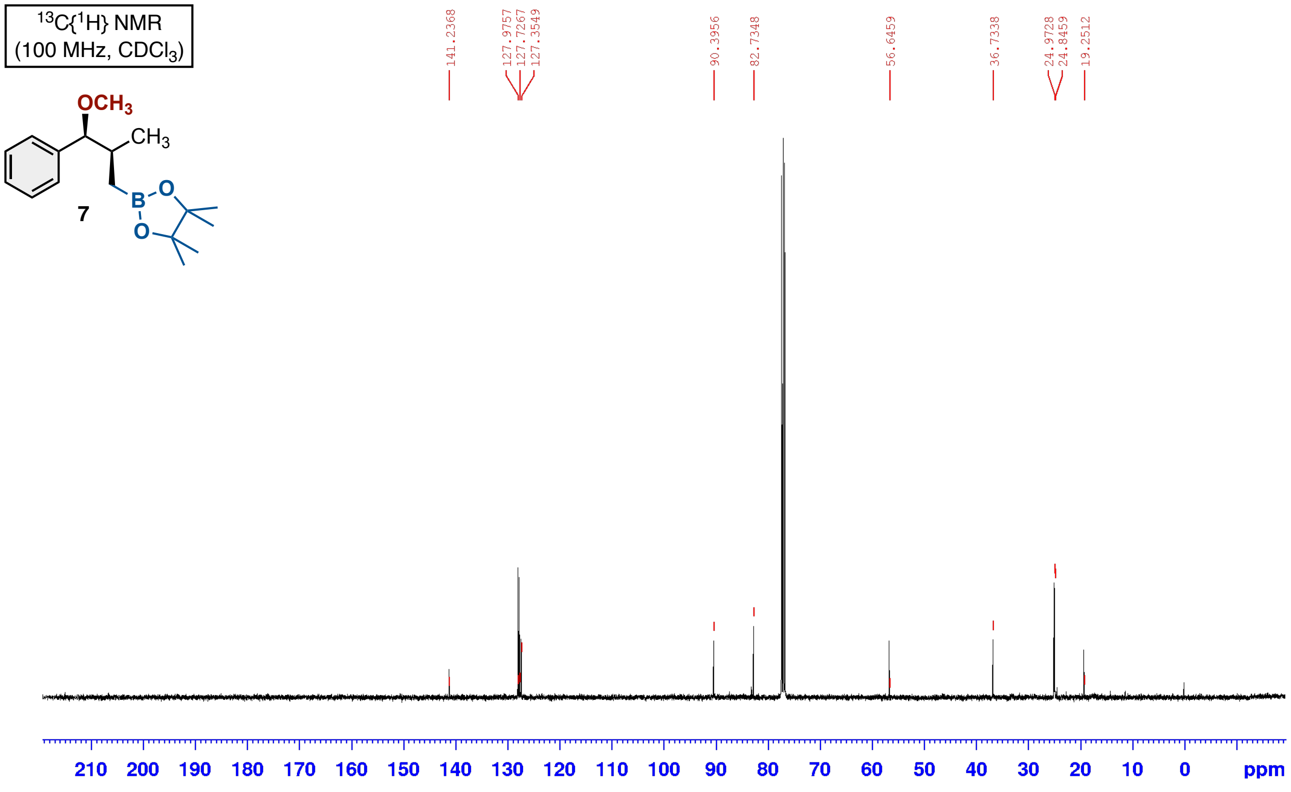

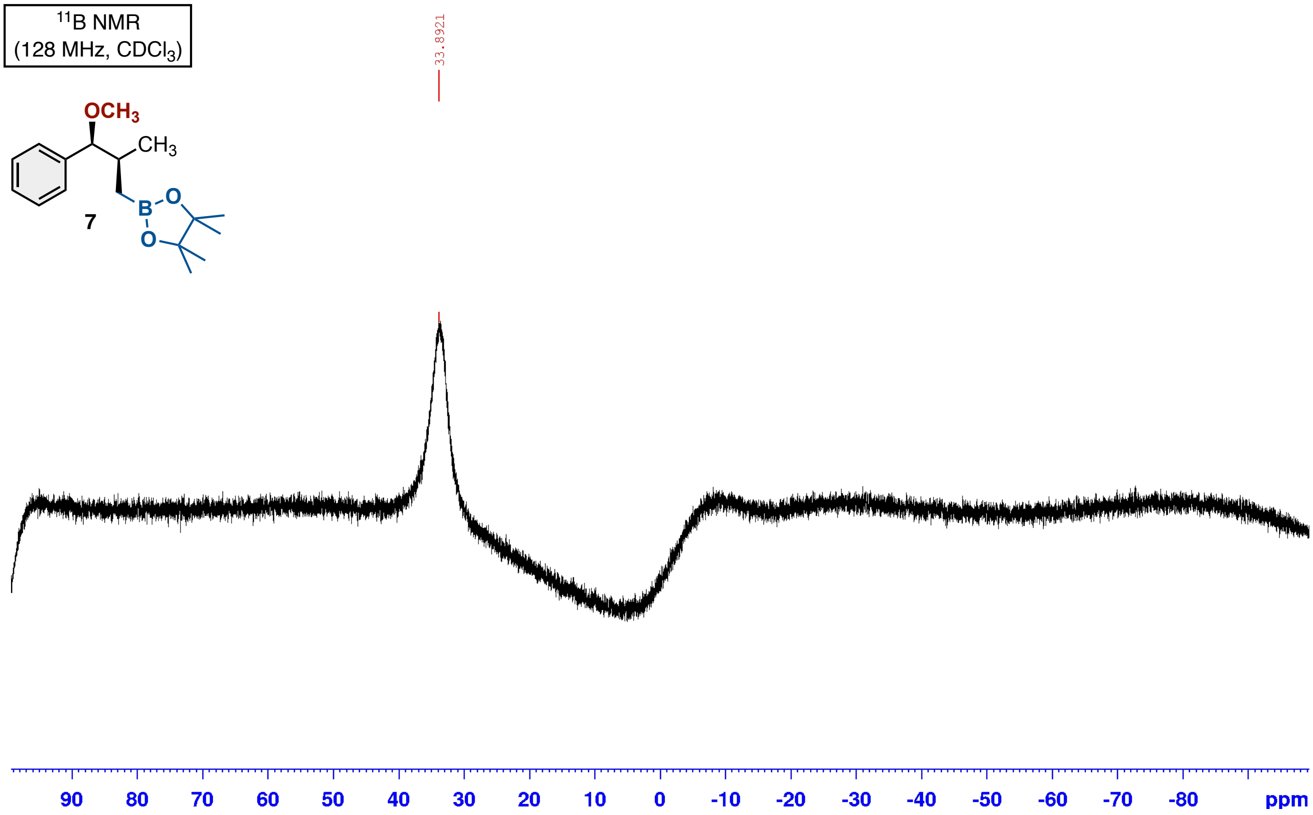

^{11}B NMR
(128 MHz, CDCl$_3$)
OCH$_3$
CH$_3$
B
O
O
7
33.8921
90
80
70
60
50
40
30
20
10
0
-10
-20
-30
-40
-50
-60
-70
-80
ppm

References

(S1) S. Igawa, M. Hashimoto, I. Kawata, M. Yashima, M. Hoshino and M. Osawa, *J. Mater. Chem. C,* 2013, **1**, 542.

(S2) G. W. Kabalka, D. Tejedor, N.-S. Li, R. R. Malladi and S. Trotman, *Tetrahedron,* 1998, **54**, 15525.

(S3) (*a*) D. Niu nad S. L. Buchwald, *J. Am. Chem. Soc.,* 2015, **137**, 9716; (*b*) Y. Dong, K. Shin, B. K. Mai, P. Liu and S. L. Buchwald, *J. Am. Chem. Soc.,* 2022, **144**, 16303.

(S4) S. Nakamura, K. Fujiwara, Y. Kojima, K. Yasui and K. Hirano, *ACS Catal.,* 2025, **15**, 8353.

(S5) X. Liu, Q. Zhu, D. Chen, L. Wang, L. Jin and C. Liu, *Angew. Chem. Int. Ed.,* 2020, **59**, 2745.

(S6) I. Shibata, T. Yoshida, T. Kawakami, A. Baba and H. Matsuda, *J. Org. Chem.,* 1992, **57**, 4049.

(S7) (*a*) G. H. Posner and D. Z. Rogers, *J. Am. Chem. Soc.,* 1977, **99**, 8214–8218; (*b*) T. Hatsui and H. Takeshita, *Bull. Chem. Soc. Jpn.,* 1980, **53**, 2655.
